# Green Synthesis of Spiro Compounds with Potential Anticancer Activity through Knoevenagel/Michael/Cyclization Multicomponent Domino Reactions Organocatalyzed by Ionic Liquid and Microwave-Assisted

**DOI:** 10.3390/molecules27228051

**Published:** 2022-11-19

**Authors:** Regina Westphal, Eclair Venturini Filho, Laiza Bruzadelle Loureiro, Cláudio Francisco Tormena, Claudia Pessoa, Celina de Jesus Guimarães, Mariana Palmeira Manso, Rodolfo Goetze Fiorot, Vinicius Rangel Campos, Jackson Antônio Lamounier Camargos Resende, Fabrizio Medici, Sandro José Greco

**Affiliations:** 1 Chemistry Department, Federal University of Espírito Santo, Fernando Ferrari Avenue 514, Vitória 29075-910, ES, Brazil; 2 Institute of Chemistry, University of Campinas, Josué de Castro Street, Campinas 13083-970, SP, Brazil; 3 Department of Physiology and Pharmacology, Faculty of Medicine, Federal University of Ceará, Fortaleza 60430-275, CE, Brazil; 4 Campus do Valonguinho, Institute of Chemistry, Fluminense Federal University, Niterói 24020-141, RJ, Brazil; 5 Exact and Earth Sciences Institute, Federal University of Mato Grosso, Campus Universitário do Araguaia, Pontal do Araguaia 78698-000, MT, Brazil; 6 Chemistry Department, University of Milan, Via Golgi 19, 20133 Milan, Italy

**Keywords:** spiro compound, domino reaction, multicomponent reaction, ionic liquid, anticancer activity

## Abstract

In this work a microwave-assisted Knoevenagel/Michael/cyclization multicomponent domino methodology, using ethanol as solvent and the ionic liquid 1-methylimidazolium chloride as catalyst was developed for the synthesis of spiro compounds. The reaction conditions considered ideal were determined from a methodological study varying solvent, catalyst, amount of catalyst, temperature, and heating mode. Finally, the generality of the methodology was evaluated by exploring the scope of the reaction, varying the starting materials (isatin, malononitrile, and barbituric acid). Overall, the twelve spiro compounds were synthesized in good yields (43–98%) and the X-ray structure of compound **1b** was obtained. In addition, the in vitro antiproliferative activities of the spirocycles against four types of human cancer cell lines including HCT116 (human colon carcinoma), PC3 (prostate carcinoma), HL60 (promyelocytic leukemia), and SNB19 (astrocytoma) were screened by MTT-based assay. It is noteworthy that spiro compound **1c** inhibited the four cell lines tested with the lowest IC_50_ values: 52.81 µM for HCT116, 74.40 µM for PC3, 101 µM for SNB19, and 49.72 µM for HL60.

## 1. Introduction

Spiro compounds are organic substances formed by at least two rings linked through only one atom, called a spiro-atom. These molecules are present in many natural products isolated from various sources [1,2,3,4,5]. Furthermore, they have a wide range of biological activities [6] and, therefore, have attracted the attention of many researchers as a primary framework for the discovery of new drugs.

However, the synthesis of spiro compounds is a big challenge for synthetic organic chemists, given their 3D structural properties, conformational rigidity, and intrinsic complexity. In this sense, domino reactions have gained prominence in the literature to obtain this class of compounds [7], because they are direct processes that generally have a simplified operating procedure, reduced reaction time, lower waste generation, and lower cost [8].

Furthermore, as in the present work, in many cases, domino synthesis requires more than two starting materials, which participate simultaneously in the reaction. Thus, these reactions are like a branch of multicomponent reactions. Therefore, the advantages of multicomponent processes also apply to domino sequences, namely: the generation of structurally diverse products, varied possibilities of synthesis, highly convergent routes, and better reaction efficiency [8].

Regarding the use of catalysts in the synthesis of spiro compounds, most examples found in the literature involve metal catalysis, organocatalysis, and synergistic methodologies [9,10,11]. It is worth mentioning that the use of ionic liquids as organocatalysts has become increasingly pungent, although still incipient, given the characteristic physicochemical properties of these substances, as well as the possibility of altering their solubility, hydrophilic/hydrophobic character, acidity/basicity, and coordination ability from the modification of their cations and anions, enabling the construction of ionic liquids with specific applications in several fields, including catalysis [12,13].

**Figure 1:**
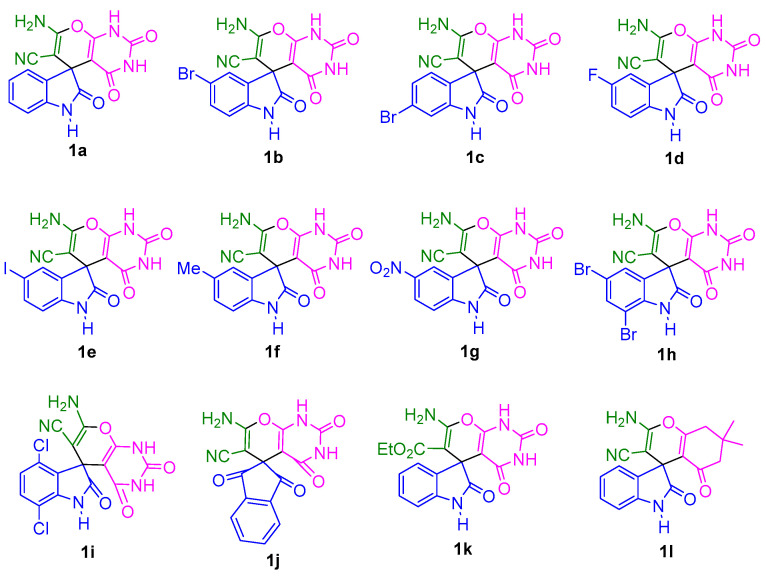
Synthesized spiro compounds.

Thus, in the present work, a Knoevenagel/Michael/cyclization multicomponent domino methodology organocatalyzed by ionic liquid and microwave-assisted was used for the synthesis of twelve spiro compounds with good yields (Figure 1).

## 2. Results and Discussion

**Scheme 1:**
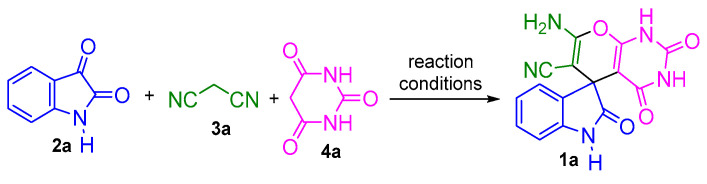
Synthesis of spiro compound **1a**.

This study started with the optimization of reaction conditions for the synthesis of spiro compounds. Therefore, the synthesis of the **1a**, using as starting materials isatin **2a**, malononitrile **3a,** and barbituric acid **4a**, in equimolar concentrations, was taken as a model reaction (Scheme 1).

Initially, the evaluation of the best solvent for the reaction was carried out under conventional heating at reflux temperature, using the ionic liquid (IL) 1-methylimidazolium chloride **5** (0.3 mmol) as catalyst. Table 1 shows the solvents used and yields observed after 24 h of reaction. It is noted that higher yields were obtained in polar solvents (Entries 1, 2, and 7), probably due to the better solubility of the starting materials in them, as well as the possible stabilization of the charged reactive species generated in the reaction medium and of the polar transition states of Knoevenagel condensation and Michael addition [14]. As result, the activation energy of the process is reduced, favoring it and, consequently, providing higher conversions. Therefore, the best result was achieved using ethanol as solvent (Entry 7). Then, the study of the catalyst was carried out to evaluate the effectiveness of the IL 1-methylimidazolium chloride, used for the first time as a catalyst in this type of reaction, against other catalysts (Entries 7–11). To our delight, the best result was obtained with the IL. It is worth mentioning that the choice of the other catalysts (Entries 8–11) was based on the literature [15,16,17,18]. Once the effectiveness of the IL was confirmed, its stoichiometry was also evaluated (Entries 7 and 12–14). Thus, it was observed that when using a stoichiometry lower than 30 mol%, the reaction yield decreased, therefore, the ideal amount of IL is the one described in entry 7. 

Finally, the effect of the reaction temperature was evaluated, as shown in Table 2. Thus, the previously optimized reaction was carried out at 0 °C, through an ice bath, and at room temperature (Entries 1 and 2). Under these conditions longer reaction times were required, and lower yields were obtained when compared to the reaction under reflux (Entry 3). Finally, to reduce the reaction time, it was decided to carry out the model reaction under microwave irradiation at 80 °C (Entry 4), conditions that were considered optimal for the reaction because they provided the formation of the product after 2 h with 91% yield.

**Scheme 2:**
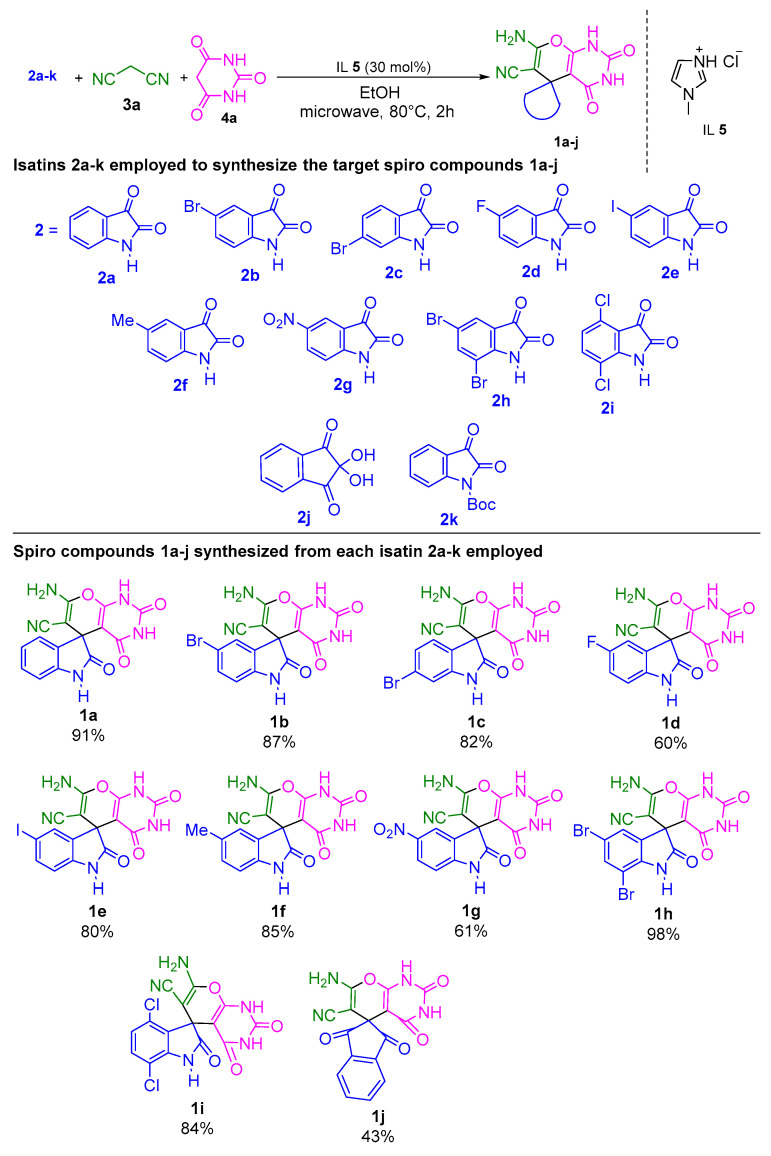
Reaction scope increase with compound isatins (**2a–k**) variation.

With the reaction conditions fully optimized, the scope of the reaction was explored. Thus, initially, compound **2** was varied, as shown in Scheme 2, obtaining spirocycles **1a**–**j** in yields from 43 to 98%. The standard reaction was also carried out with the isatin nitrogen atom protected by the di-tert-butyl dicarbonate (Boc) group, compound **2k**. However, spiro compound **1a** was formed, probably due to the acidity of the medium promoted by the protic IL **5**, resulting in nitrogen deprotection.

**Scheme 3:**
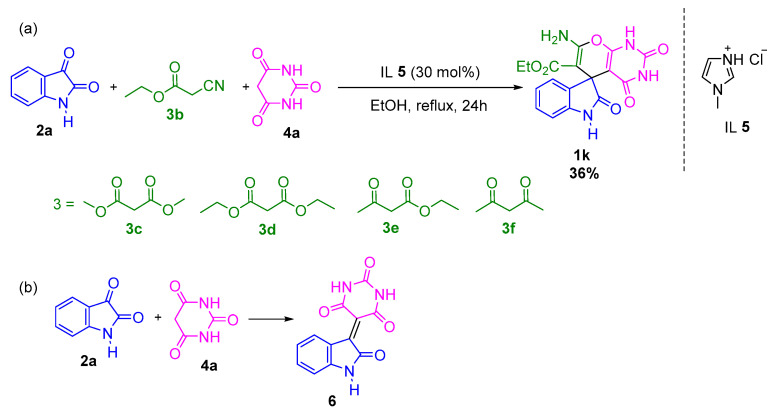
(**a**) Reaction scope increase with compound **3** variation; (**b**) Knoevenagel adduct formation.

Then, compounds **3b**–**f** (Scheme 3a) were used instead of malonitrile **3a**. By replacing malononitrile **3a** with ethyl cyanoacetate **3b**, the desired spiro compound **1k** could be obtained in trace amount. So, it was decided to repeat the reaction under conventional heating to verify if the formation of **1k** would occur under such conditions. Surprisingly, the spirocycle was obtained in 36% yield. In addition, in an unprecedented way in the literature, an attempt has been made to replace **3a** with dimethyl malonate **3c** and diethyl malonate **3d** diesters, as well as ethyl acetoacetate **3e** and acetylacetone **3f**, since such substances are normally used only in place of barbituric acid **4a** [18,19,20,21,22,23,24]. However, the respective products were not formed. The only compound isolated in all reactions was the Knoevenagel intermediate between isatin **2a** and barbituric acid **4a**.

Analyzing the pKa values of **3a**–**f** and **4a** [25], it is noted that the methylene compounds **3c**–**f** (pKa = 16.4, 16.4, 14.2, and 13.3, respectively), are less acidic than **4a** (pKa = 8.4), which justifies the non-formation of spirocycles, but of the Knoevenagel product **6** (Scheme 3b).

On the other hand, it is also observed that **3a** and **3b** (pKa = 11.0 and 13.1, respectively), are equally less acidic than **4a**; however, in these cases, the formation of spiro compounds **1a**–**j** and **1k** occurred, probably due to greater nucleophilicity of **3a** and **3b**. This is because the resonance structures of conjugate bases of **3a** and **3b** are less stabilized than those of **4a**, since the delocalization of the negative charge passes through nitrogen atoms, which are less electronegative than the oxygen atoms, through which the negative charge passes in the **4a** resonance structures.

**Scheme 4:**
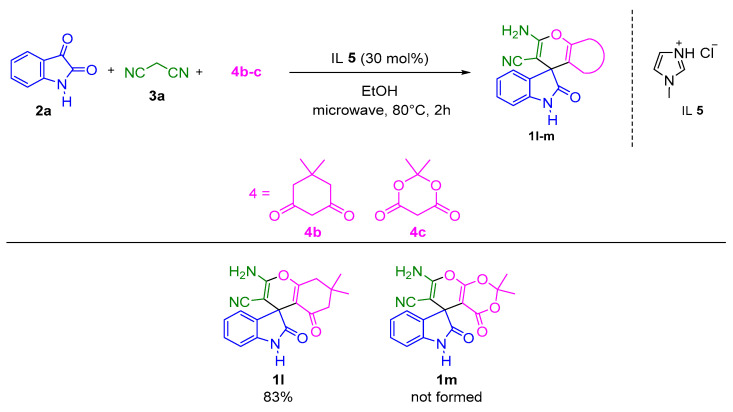
Reaction scope increase with compound **4** variation.

**Scheme 5:**
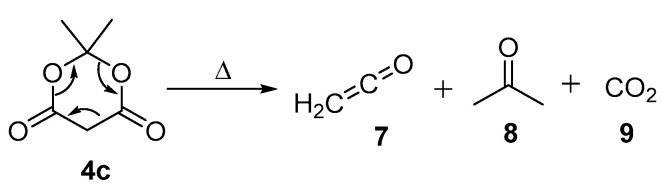
Retro-hetero Diels-Alder of Meldrum’s Acid.

Finally, compound **4** was also varied, as shown in Scheme 4. The replacement of barbituric acid **4a** by dimedone **4b** enabled the formation of spirocycle **1l** with a yield of 83%. However, a mixture of substances that was difficult to purify was obtained when Meldrum’s acid **4c** was used in place of barbituric acid **4a**. This was probably due to the thermal instability of Meldrum’s acid, which at temperatures close to 80 °C and long reaction times undergoes a retro-hetero-Diels-Alder reaction, giving rise to the highly reactive ketene **7**, acetone **8,** and carbon dioxide **9**, as shown in Scheme 5 [26,27]. Another possibility is the degradation of Meldrum’s acid into its precursor, malonic acid, as reported by Dourado (2018) [28].

**Scheme 6:**
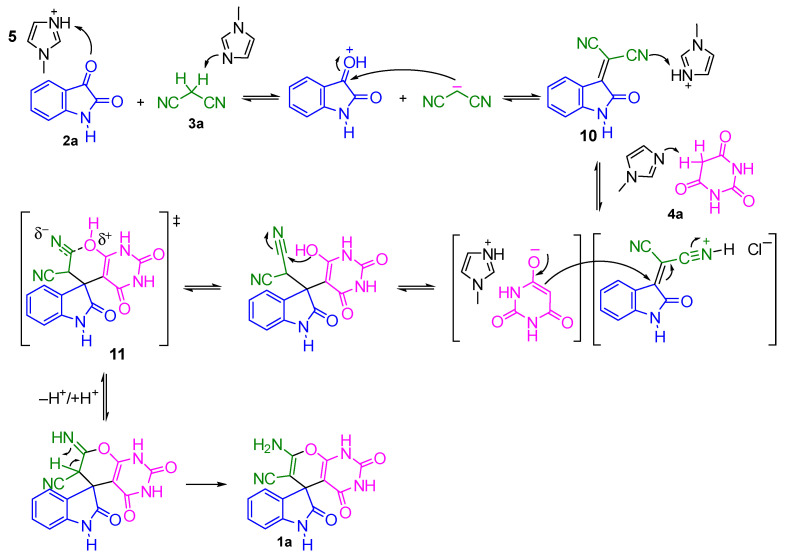
Suggested mechanism for the synthesis of spiro compounds **1a**–**j**, **1k**, and **1l**.

The proposed mechanism is illustrated for the synthesis of **1a,** as described in Scheme 6. Initially, isatylidene–malononitrile intermediate **10** from the Knoevenagel condensation between **2a** and **3a** is formed. Then, a Michael addition between **4a** and **10**, followed by cyclization and tautomerization, gives **1a**. The catalytic role of **5** is possibly due to the stabilization of the suggested polar transition state **11**, as well as the activation of species **4a** and **10** participating in the Michael addition step. It is worth mentioning that intermediate **10** could be isolated and characterized, which contributed to support this mechanistic proposal, which is also valid for the other synthesized compounds. In the infrared spectrum of **10,** it was possible to highlight a frequency in infrared spectrum at 2230 cm^−1^, referring to the nitriles present in the structure, as well as at 1619 cm^−1^, referring to the C=C bond formed due to the Knoevenagel condensation. As expected, in the ^1^H-NMR spectrum, only the signals referring to the aromatic hydrogens and the NH of the isatin were identified. Finally, in the ^13^C-NMR spectrum, the presence of signals at 112.0 ppm and 81.0 ppm referring to the nitrile carbons and the central carbon of malononitrile (added to isatin), respectively, were assigned.

**Figure 2:**
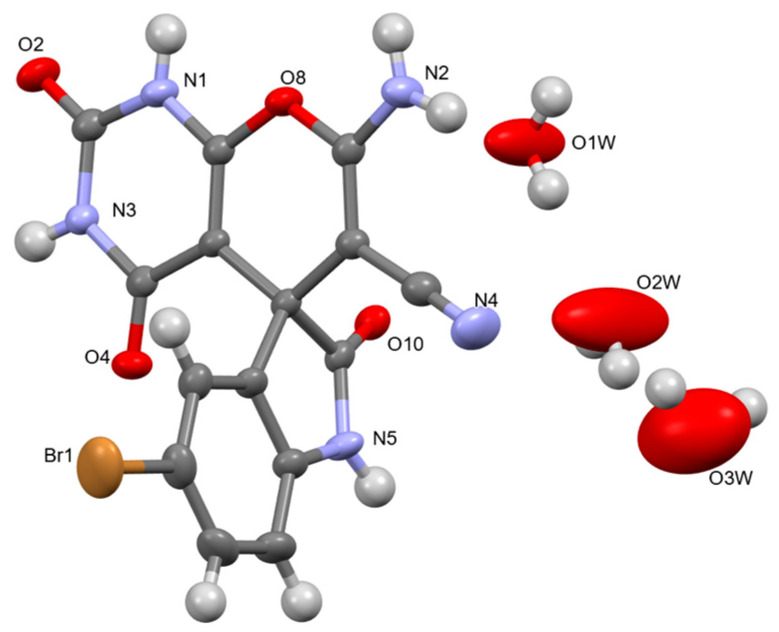
Molecular representation and atomic labeling scheme of spiro compound **1b** obtained from single crystal X-ray diffraction technique. O1W, O2W, and O3W refers to the oxygen atoms from the three water molecules co-crystallized with the spiro compound.

X-ray structure determination of compound **1b** was obtained from methanol under slow evaporation. The crystal structure has been described in the triclinic P1 space group. Three molecules of water of crystallization were observed in the asymmetric unit of the crystal. Crystalline packing is maintained by hydrogen bonding and π-π stacking. Figure 2 shows the Oak Ridge thermal ellipsoid plot (ORTEP) diagram of the compound. Further crystallographic details for the structure reported in this paper may be obtained from the Cambridge Crystallographic Data Center, on quoting the depository numbers CCDC-2213864.

The newly synthesized spiro compounds **1a–l** were screened for their *in vitro* antiproliferative activities against four types of human cancer cell lines including HCT116 (human colon carcinoma), PC3 (prostate carcinoma), HL60 (promyelocytic leukemia), and SNB19 (astrocytoma) by MTT-based assay. The results are represented as the half maximal inhibitory concentration (IC_50_-μM) values in Table 3.

**Figure 3:**
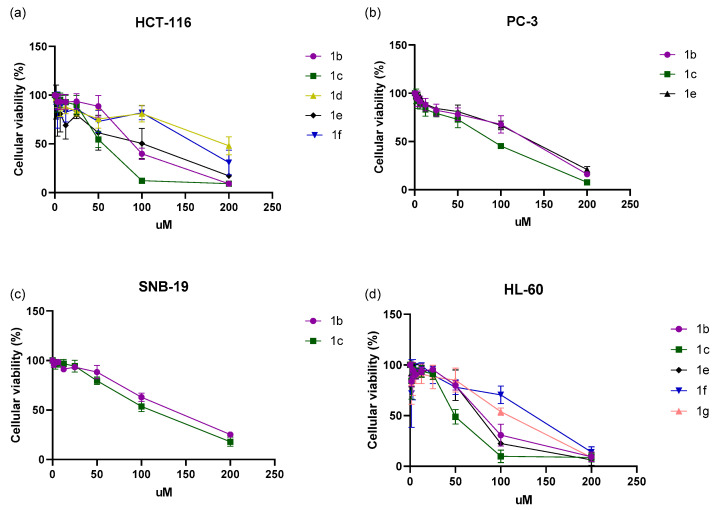
Inhibitory concentration means (IC_50_) of the synthesized compounds **1b**–**c** and **1e**–**g** against the (**a**) HCT116; (**b**) PC3; (**c**) HL60; and (**d**) SNB19 tumoral cell lines.

Graph representation of inhibitory concentration mean (IC_50_) of the compounds **1b–c** and **1e–g** against HCT116, PC3, HL60, and SNB19 tumoral cell lines are shown in the Figure 3. 

Unfortunately, the antiproliferative activity of the tested compounds was only moderate. The only spiro compounds that inhibited the four cell lines tested were **1b** and **1c**, the latter with the lowest IC_50_ values: 52.81 µM for HCT116; 74.40 µM for PC3; 101 µM for SNB19, and 49.72 µM for HL60.

## 3. Experimental Session

### 3.1. Materials and Methods

The necessary reagents and solvents were used without prior purification. The microwave reactor CEM Discover (CEM Corporation, Matthews, NC, USA) was used with a power of 150 W and temperature monitoring through an infrared monitoring system. The reactions were followed by thin-layer chromatography using aluminum coated with silica gel UV254 (250 µm, 20 × 20 cm). The determination of the decomposition/melting point of the synthesized compounds was carried out in the Fisatom 431D digital equipment (Fisatom, São Paulo, Brazil). Infrared spectra were obtained on the Agilent Cary 630 FTIR Spectrometer (Agilent, Santa Clara, CA, USA) using as parameters 16 scans and resolution of length of 4 cm^−1^, in attenuated total reflectance (ATR) mode, with horizontal zinc selenide (ZnSe) crystal. All analyses were performed in a wavelength range of 4000 to 400 cm^−1^. The ^1^H-NMR spectra of the compounds **6** and **10** were obtained in DMSO-*d*_6_ (Sigma-Aldrich, St. Louis, MO, USA) in a Varian 400 MHz spectrometer (Varian, Palo Alto, Santa Clara, CA, USA) with a 5 mm broadband 1H/X/D probe. The NMR spectra of the compounds **1a**, **1h**, and **1k** were obtained in DMSO-*d*_6_ (Sigma-Aldrich, St. Louis, MO, USA) in a Bruker Advance III 500 MHz (Bruker, Rheinstetten, Germany) equipped with a 5 mm smart BBO probe. The NMR spectra of the compounds **1b**–**g**, **1i**, and **1l** were obtained in DMSO-*d*_6_ (Sigma-Aldrich, St. Louis, MO, USA) in a Bruker Advance III 400 MHz (Bruker, Rheinstetten, Germany) equipped with a 5 mm BBI probe. Finally, the NMR spectra of the compound **1j** were obtained in D_2_O (Sigma-Aldrich, St. Louis, MO, USA) in a Bruker Advance III 600 MHz (Bruker, Rheinstetten, Germany) equipped with a 5 mm TBO probe. Chemical shifts δ were expressed in ppm relative to the TMS. Mass spectra were obtained using a high-resolution spectrometer (model 9.4 T Solarix, Bruker Daltonics, Bremen, Germany), operated in positive and negative ionization mode with ionizing electrospray, ESI(+)-FT-ICR (MS) and ESI(-)-FT-ICR (MS), respectively. The acquisition of FT-ICR MS spectra was performed with resolving power of m/Δm50% ≈ 500,000, where Δm50% is the entire peak with m/z 400 being half the maximum height and mass accuracy < 1 ppm. Infrared, NMR and mass spectra of all synthesized compounds are available in the Supplementary Material of this article.

### 3.2. General Procedure

In a 10 mL flask were added 1 mmol of **2a**–**j**, 1 mmol of **3a**–**b**, 1 mmol of **4a**–**b** and 0.3 mmol of the ionic liquid 1-methylimidazolium chloride **5** in 6 mL of ethanol. The reaction was kept under stirring and heated by microwave irradiation at 80 °C for 2 h, with a power of 150 W. The solid obtained was filtered and washed with ice-cold acetonitrile.

*7′-amino-2,2′,4′-trioxo-1′,2′,3′,4′-tetrahydrospiro[indoline-3,5′-pyrano[2,3-d]pyrimidine]-6′-carbonitrile (***1a**); mp: 262–264 °C; IR ν_max_ (cm^−1^, ATR): 3353 e 3304 (ν NH_2_), 3133 (ν NH), 2203 (ν nitrile), 1666 (ν C=O), 1336 (ν C-N), 1113 (ν C-O); ^1^H-NMR (500 MHz, DMSO-*d*_6_): δ_H_ 12.29 (1H, br s, H17), 11.10 (1H, s, H19), 10.46 (1H, s, H7), 7.35 (2H, s, H14), 7.16 (1H, dt, J = 7.7, 1.3 Hz, H2), 7.13 (1H, br d, J = 7.4 Hz, H6), 6.91 (1H, dt, J = 7.5, 0.9 Hz, H1), 6.78 (1H, br d, J = 7.7 Hz, H3); ^13^C-NMR (126 MHz, DMSO-*d*_6_): δ_C_ 177.6 (s, C8), 161.4 (s, C16), 158.2 (s, C12), 153.4 (s, C11), 149.2 (s, C18), 142.1 (s, C4), 133.5 (s, C5), 128.4 (s, C2), 123.7 (s, C6), 121.7 (s, C1), 116.9 (s, C15), 109.2 (s, C3), 86.8 (s, C10), 57.9 (s, C13), 46.7 (s, C9); ESI (–) FT-ICR MS: [C_15_H_8_N_5_O_4_]^−^ exp = 322.05812 *m/z*, calc = 322.05818 *m/z* (err = 0.19 ppm), [C_30_H_17_N_10_O_8_]^−^ exp = 645.12356 *m/z*, calc = 645.12363 *m/z* (err = 0.11 ppm), [C_45_H_26_N_15_O_12_]^−^ exp = 968.18860 *m/z*, calc = 968.18908 *m/z* (err = 0.50 ppm).

*7′-amino-5-bromo-2,2′,4′-trioxo-1′,2′,3′,4′-tetrahydrospiro[indoline-3,5′-pyrano[2,3-d]pyrimidine]-6′-carbonitrile* (**1b**); mp: 226 °C; IR ν_max_ (cm^−1^, ATR): 3142 (ν NH), 2197 (ν nitrile), 1685 (ν C=O), 1338 (ν C-N), 1116 (ν C-O); ^1^H-NMR (400 MHz, DMSO-*d*_6_): δ_H_ 12.30 (1H, br s, H17), 11.14 (1H, s, H19), 10.61 (1H, s, H7), 7.44 (1H, d, J = 2.1 Hz, H6), 7.43 (2H, s, H14), 7.34 (1H, dd, J = 8.2, 2.1 Hz, H2), 6.76 (1H, d, J = 8.2 Hz, H3); ^13^C-NMR (101 MHz, DMSO-*d*_6_): δ_C_ 177.4 (s, C8), 161.5 (s, C18), 158.4 (s, C12), 153.6 (s, C11), 149.3 (s, C16), 141.5 (s, C4), 136.0 (s, C5), 131.1 (s, C2), 126.8 (s, C6), 116.9 (s, C15), 113.5 (s, C1), 111.2 (s, C3), 86.3 (s, C10), 57.1 (s, C13), 46.9 (s, C9); ESI (-) FT-ICR MS: [C_15_H_7_BrN_5_O_4_]^−^ exp = 399.96852 *m/z*, calc = 399.967593 *m/z* (err = −2.31 ppm), [C_30_H_15_Br_2_N_10_O_8_]^−^ exp = 800.94508 *m/z*, calc = 800.943559 *m/z* (err = −1.90 ppm).

*7′-amino-6-bromo-2,2′,4′-trioxo-1′,2′,3′,4′-tetrahydrospiro[indoline-3,5′-pyrano[2,3-d]pyrimidine]-6′-carbonitrile* (**1c**); mp: 261 °C; IR ν_max_ (cm^−1^, ATR): 3148 (ν NH), 2201 (ν nitrile), 1685 (ν C=O), 1338 (ν C-N), 1124 (ν C-O); ^1^H-NMR (400 MHz, DMSO-*d*_6_): δ_H_ 12.34 (1H, br s, H17), 11.12 (1H, s, H19), 10.64 (1H, s, H7), 7.44 (2H, s, H14), 7.15 (1H, d, J = 7.9 Hz, H6), 7.10 (1H, dd, J = 7.9, 1.8 Hz, H2), 6.93 (1H, d, J = 1.8 Hz, H3); ^13^C-NMR (101 MHz, DMSO-*d*_6_): δ_C_ 177.6 (s, C8), 161.5 (s, C18), 158.4 (s, C12), 153.6 (s, C11), 149.2 (s, C16), 143.8 (s, C4), 132.9 (s, C5), 125.8 (s, C6), 124.4 (s, C1), 121.0 (s, C2), 116.8 (s, C15), 112.0 (s, C3), 86.3 (s, C10), 57.0 (s, C13), 46.5 (s, C9); ESI (-) FT-ICR MS: [C_15_H_7_BrN_5_O_4_]^−^ exp = 399.96855 *m/z*, calc = 399.967593 *m/z* (err = −2.40 ppm), [C_30_H_15_Br_2_N_10_O_8_]^−^ exp = 800.94542 *m/z*, calc = 800.943559 *m/z* (err = −2.32 ppm).

7*′-amino-5-fluoro-2,2′,4′-trioxo-1′,2′,3′,4′-tetrahydrospiro[indoline-3,5′-pyrano[2,3-d]pyrimidine]-6′-carbonitrile* (**1d**); mp: 210 °C (with decomposition); IR ν_max_ (cm^−1^, ATR): 3135 (ν NH), 2199 (ν nitrile), 1685 (ν C=O), 1325 (ν C-N), 1182 (ν C-O); ^1^H-NMR (400 MHz, DMSO-*d*_6_): δ_H_ 12.31 (1H, s, H17), 11.14 (1H, s, H19), 10.49 (1H, s, H7), 7.42 (2H, s, H14), 7.16 (1H, dd, J = 8.2, 2.7 Hz, H6), 6.98 (1H, ddd, J = 9.6, 8.5, 2.7 Hz, H2), 6.77 (1H, dd, J = 8.5, 4.3 Hz, H3); ^13^C-NMR (101 MHz, DMSO-*d*_6_): δ_C_ 177.7 (s, C8), 161.5 (s, C18), 158.4 (s, C12), 158.3 (d, J = 236.7 Hz, C1), 153.5 (s, C11), 149.3 (s, C16), 138.3 (d, J = 1.6 Hz, C4), 135.3 (d, J = 7.7 Hz, C5), 116.8 (s, C15), 114.6 (d, J = 23.4 Hz, C2), 111.7 (d, J = 24.9 Hz, C6), 109.9 (d, J = 8.0 Hz, C3), 86.4 (s, C10), 57.3 (s, C13), 47.2 (d, J = 1.6 Hz, C9); ESI (-) FT-ICR MS: [C_19_H_7_FN_5_O_4_]^−^ exp = 340.04864 *m/z*, calc = 340.04876 *m/z* (err = 0.34 ppm), [C_30_H_15_F_2_N_10_O_8_]^−^ exp = 681.10497 *m/z*, calc = 681.10479 *m/z* (err = −0.26 ppm).

*7′-amino-5-iodo-2,2′,4′-trioxo-1′,2′,3′,4′-tetrahydrospiro[indoline-3,5′-pyrano[2,3-d]pyrimidine]-6′-carbonitrile* (**1e**); mp: 236 °C; IR ν_max_ (cm^−1^, ATR): 3122 (NH), 2201 (ν nitrile), 1688 (ν C=O), 1340 (ν C-N), 1118 (ν C-O); ^1^H-NMR (400 MHz, DMSO-*d*_6_): δ_H_ 12.30 (1H, s, H17), 11.14 (1H, s, H19), 10.59 (1H, s, H7), 7.43 (2H, s, H14), 7.55 (1H, d, J = 1.72 Hz, H6), 7.50 (1H, dd, J = 1.80, 8.08 Hz, H2), 6.64 (1H, d, J = 8.08, H3); ^13^C-NMR (101 MHz, DMSO-*d*_6_): δ_C_ 177.1 (s, C8), 161.5 (s, C18), 158.4 (s, C12), 153.6 (s, C11), 149.3 (s, C16), 142.0 (s, C4), 137.0 (s, C5), 136.2 (s, C2), 132.2 (s, C6), 116.9 (s, C15), 111.7 (s, C3), 86.3 (s, C10), 84.7 (s, C1), 57.2 (s, C13), 46.7 (s, C9); ESI (-) FT-ICR MS: [C_19_H_7_IN_5_O_4_]^−^ exp = 447.95455 *m/z*, calc = 447.95482 *m/z* (err = 0.59 ppm); [C_30_H_15_I_2_N_10_O_8_]^−^ exp = 896.91744 *m/z*, calc = 896.91692 *m/z* (err = −0.58 ppm).

*7′-amino-5-methyl-2,2′,4′-trioxo-1′,2′,3′,4′-tetrahydrospiro[indoline-3,5′-pyrano[2,3-d]pyrimidine]-6′-carbonitrile* (**1f*)***; mp: 210–212 °C; IR ν_max_ (cm^−1^, ATR): 3261 (ν NH), 2199 (ν nitrile), 1688 (ν C=O), 1390 (ν C-N), 1111 (ν C-O); ^1^H-NMR (400 MHz, DMSO-*d*_6_): δ_H_ 12.27 (1H, s, H17), 11.10 (1H, s, H19), 10.36 (1H, s, H7), 7.33 (2H, s, H14), 6.94–6.98 (2H, m, H2, H6), 6.67 (1H, d, J = 7.92 Hz, H3), 2.21 (3H, s, Me); ^13^C-NMR (101 MHz, DMSO-*d*_6_): δ_C_ 177.6 (s, C8), 161.4 (s, C18), 158.2 (s, C12), 153.3 (s, C11), 149.3 (s, C16), 139.7 (s, C4), 133.6 (s, C5), 130.6 (s, C1), 128.7 (s, C6), 124.3 (s, C2), 117.0 (s, C15), 109.0 (s, C3), 87.0 (s, C10), 58.1 (s, C13), 46.7 (s, C9), 20.6 (s, Me); ESI (-) FT-ICR MS: [C_16_H_10_N_5_O_4_]^−^ exp = 336.07381 *m/z*, calc = 336.07383 *m/z* (err = 0.06 ppm); [C_32_H_20_N_10_O_8_Na]^−^ exp = 695.13716 *m/z*, calc = 695.13688 *m/z* (err = −0.40 ppm).

*7′-amino-5-nitro-2,2′,4′-trioxo-1′,2′,3′,4′-tetrahydrospiro[indoline-3,5′-pyrano[2,3-d]pyrimidine]-6′-carbonitrile* (**1g**); mp: 230 °C (with decomposition); IR ν_max_ (cm^−1^, ATR): 3122 (ν NH), 2199 (ν nitrile), 1683 (ν C=O), 1390 (ν C-N), 1124 (ν C-O); ^1^H-NMR (400 MHz, DMSO-*d*_6_): δ_H_ 12.42 (1H, s, H17), 11.22 (1H, s, H19), 11.18 (1H, s, H7), 8.25 (1H, d, J = 2.32 Hz, H6), 8.17 (1H, dd, J = 2.40, 8.60 Hz, H2), 7.56 (2H, s, H14), 7.02 (1H, d, J = 8.60 Hz, H3); ^13^C-NMR (101 MHz, DMSO-*d*_6_): δ_C_ 178.4 (s, C8), 161.7 (s, C18), 158.7 (s, C12), 154.0 (s, C11), 149.3 (s, C16), 148.6 (s, C1), 142.6 (s, C4), 134.6 (s, C5), 126.0 (s, C2), 120.0 (s, C6), 116.8 (s, C15), 109.4 (s, C3), 85.9 (s, C10), 56.2 (s, C13), 46.9 (s, C9); ESI (-) FT-ICR MS: [C_15_H_7_N_6_O_6_]^−^ exp = 367.04311 *m/z*, calc = 367.04326 *m/z* (err = 0.39 ppm); [C_30_H_15_N_12_O_12_]^−^ exp = 735.09388 *m/z*, calc = 735.09379 *m/z* (err = −0.12 ppm).

*7′-amino-5,7-dibromo-2,2′,4′-trioxo-1′,2′,3′,4′-tetrahydrospiro[indoline-3,5′-pyrano[2,3-d]pyrimidine]-6′-carbonitrile* (**1h**); mp: 180 °C (with decomposition); IR ν_max_ (cm^−1^, ATR): 3166 (ν NH), 2199 (ν nitrile), 1685 (ν C=O), 1342 (ν C-N), 1148 (ν C-O); ^1^H-NMR (500 MHz, DMSO-*d*_6_): δ_H_ 12.39 (1H, br s, H17), 11.20 (1H, s, H19), 10.46 (1H, s, H7), 7.61 (1H, d, J = 1.8 Hz, H2), 7.53 (3H, d, J = 1.8 Hz, H6 e H14); ^13^C-NMR (126 MHz, DMSO-*d*_6_): δ_C_ 177.39 (s, C8), 161.57 (s, C18), 158.47 (s, C12), 153.72 (s, C11), 149.26 (s, C16), 141.17 (s, C4), 137.11 (s, C5), 133.09 (s, C2), 126.20 (s, C6), 116.75 (s, C15), 114.01 (s, C1), 102.38 (s, C3), 85.99 (s, C10), 56.59 (s, C13), 48.04 (s, C9); ESI (-) FT-ICR MS: [C_15_H_6_Br_2_N_5_O_4_]^−^ exp = 477.87893 *m/z*, calc = 477.87920 *m/z* (err = 0.56 ppm).

*7′-amino-4,7-dichloro-2,2′,4′-trioxo-1′,2′,3′,4′-tetrahydrospiro[indoline-3,5′-pyrano[2,3-d]pyrimidine]-6′-carbonitrile* (**1i**); mp: >300 °C; IR ν_max_ (cm^−1^, ATR): 3161 (ν NH), 2203 (ν nitrile), 1694 (ν C=O), 1340 (ν C-N), 1118 (ν C-O); ^1^H-NMR (400 MHz, DMSO-*d*_6_): δ_H_ 12.53 (1H, s, H17), 11.30 (1H, s, H19), 11.24 (1H. s, H7), 7.63 (2H, s, H14), 6.99 (1H, d, J = 8.72 Hz, H1), 7.34 (1H, d, J = 8.76 Hz, H2); ^13^C-NMR (101 MHz, DMSO-*d*_6_): δ_C_ 176.8 (s, C8), 161.4 (s, C18), 159.4 (s, C12), 154.0 (s, C11), 149.1 (s, C16), 141.7 (s, C4), 130.3 (s, C2), 129.4 (s, C5), 128.0 (s, C6), 123.4 (s, C1), 116,5 (s, C15), 112.9 (s, C3), 84.8 (s, C10), 54.2 (s, C13), 48.2 (s, C9); ESI (-) FT-ICR MS: [C_15_H_6_Cl_2_N_5_O_4_]^−^ exp = 389.98002 *m/z*, calc = 389.98023 *m/z* (err = 0.53 ppm).

*7′-amino-1,2′,3,4′-tetraoxo-1,1′,2′,3,3′,4′-hexahydrospiro[indene-2,5′-pyrano[2,3-d]pyrimidine]-6′-carbonitrile* (**1j**); mp: 244 °C; IR ν_max_ (cm^−1^, ATR): 3163 (ν NH), 2210 (ν nitrile), 1677 (ν C=O), 1329 (ν C-N), 1256 (ν C-O); ^1^H-NMR (600 MHz, D_2_O): δ_H_ 8.64 (1H, s, H17), 7.99–7.95 (2H, m, H3, H6), 7.91–7.87 (2H, m, H1, H2), 7.43 (1H, d, J = 1.5 Hz, H19), 3.91 (2H, s, H14); ^13^C-NMR (151 MHz, D_2_O): δ_C_ 205.2 (s, C7or C9), 201.0 (s, C9 or C7), 170.2 (s, C16 or C18), 169.5 (s, C18 or C16), 155.6 (s, C11), 144.8 (s, C12), 138.6 (s, C3, C6), 138.0 (s, C4, C5), 126.3 (s, C1, C2), 126.0 (s, C10), 122.5 (s, C15), 62.1 (s, C13), 38.5 (s, C8); ESI (-) FT-ICR MS: [C_16_H_7_N_4_O_5_]^−^ exp = 335.04216 *m/z*, calc = 335.04219 *m/z* (err = 0.09 ppm), [C_32_H_15_N_8_O_10_]^−^ exp = 671.09176 *m/z*, calc = 671.09166 *m/z* (err = −0.15 ppm).

*Ethyl 7′-amino-2,2′,4′-trioxo-1′,2′,3′,4′-tetrahydrospiro[indoline-3,5′-pyrano[2,3-d]pyrimidine]-6′-carboxylate* (**1k**); mp: 208–210 °C; IV ν_max_ (cm^−1^, ATR): 3192 (ν NH), 1672 (ν C=O), 1327 (ν C-N), 1116 (ν C-O); ^1^H-NMR (500 MHz, DMSO-*d*_6_): δ_H_ 12.15 (1H, br s, H17), 10.95 (1H, s, H19), 10.22 (1H, s, H7), 7.93 (2H, s, H14), 7.06 (1H, dt, J = 7.6, 1.3 Hz, H2), 6.94 (1H, br d, J = 7.2 Hz, H6), 6.78 (1H, dt, J = 7.4, 0.9 Hz, H1), 6.67 (1H, br d, J = 7.6 Hz, H3), 3.78 (2H, m, H20 e H20′), 0.78 (3H, t, J = 7.1 Hz, H21); ^13^C-NMR (126 MHz, DMSO-*d*_6_): δ_C_ 179.3 (s, C8), 167.4 (s, C5), 161.2 (s, C16), 158.6 (s, C12), 152.2 (s, C11), 149.1 (s, C18), 144.0 (s, C4), 135.3 (s, C5), 127.3 (s, C2), 122.7 (s, C6), 120.6 (s, C1), 108.2 (s, C3), 89.2 (s, C10), 76.3 (s, C13), 59.1 (s, C20), 46.2 (s, C9), 13.0 (s, C21); ESI (-) FT-ICR MS: [C_17_H_13_N_4_O_6_]^−^ exp = 369.08404 *m/z*, calc = 369.08406 *m/z* (err = 0.05 ppm).

*2-amino-7,7-dimethyl-2′,5-dioxo-5,6,7,8-tetrahydrospiro[chromene-4,3′-indoline]-3-carbonitrile* (**1l**); mp: 300 °C; IR ν_max_ (cm^−1^, ATR): 3306 (ν NH), 2960 (ν CH_2_), 2192 (ν nitrile), 1653 (ν C=O), 1346 (ν C-N), 1221 (ν C-O); ^1^H-NMR (400 MHz, DMSO-*d*_6_): δ_H_ 10.39 (1H, s, H7), 7.21 (2H, s, H14), 7.14 (1H, dt, J = 7.6, 1.3 Hz, H2), 6.97 (1H, br d, J = 7.4 Hz, H6), 6.89 (1H, dt, J = 7.5, 0.9 Hz, H1), 6.79 (1H, br d, J = 7.6 Hz, H3), 2.58 (1H, d, J = 17.5 Hz, H19), 2.53 (1, d, J = 17.5 Hz, H19′), 2.17 (1H, d, J = 16.0 Hz, H17), 2.09 (1H, d, J = 16.0 Hz, H17′), 1.03 (3H, s, 20), 1.00 (3H, s, 21); ^13^C-NMR (101 MHz, DMSO-*d*_6_): δ_C_ 194.8 (s, C16), 178.0 (s, C8), 164.1 (s, C11), 158.8 (s, C12), 142.0 (s, C4), 134.4 (s, C5), 128.1 (s, C2), 123.0 (s, C6), 121.7 (s, C1), 117.3 (s, C15), 110.8 (s, C10), 109.2 (s, C3), 57.5 (s, C13), 50.0 (s, C17), 46.8 (s, C9), 40.0 (s, C19), 31.9 (s, C18), 27.6 (s, C20), 27.0 (s, C21); ESI (+) FT-ICR MS: [C_19_H_17_N_3_O_3_Na]^+^ exp = 358.11626 *m/z*, calc = 358.11621 *m/z* (err = −0.12 ppm), [C_38_H_34_N_6_O_6_Na]^+^ exp = 693.24332 *m/z*, calc = 693.24320 *m/z* (err = −0.17 ppm), [C_57_H_51_N_9_O_9_Na]^+^ exp = 1028.37086 *m/z*, calc = 1028.37020 *m/z* (err = −0.65 ppm).

*5-(2-oxoindolin-3-ylidene)pyrimidine-2,4,6(1H,3H,5H)-trione* (**6**); mp: 220 °C (with decomposition); IR ν_max_ (cm^−1^, ATR): 3260 (ν NH), 1681 (ν C=O), 1618 (ν C=C conjugated), 1331 (ν C-N); ^1^H-NMR (400 MHz, DMSO-*d*_6_): δ_H_ 11.17 (2H, s, NH), 10.55 (1H, s, NH), 7.17–7.09 (2H, m, H aromatic), 6.89 (1H, t, J = 7.6 Hz, H aromatic), 6.67 (1H, d, J = 7.6 Hz, H aromatic).

*2-(2-oxoindolin-3-ylidene)malononitrile* (**10**); mp: 124–126 °C; IV ν_max_ (cm^−1^, ATR): 3219 (ν NH), 2230 (ν nitrile), 1711(ν C=O), 1619 (ν C=C conjugated), 1327 (ν C-N); ^1^H-NMR (400 MHz, DMSO-*d*_6_): δ_H_ 11.17 (1H, s, NH), 7.85 (1H, d, J = 7.8 Hz, H aromatic), 7.54 (1H, td, J = 7.8, 0.9 Hz, H aromatic), 7.10 (1H, td, J = 7.8, 0.9 Hz, H aromatic), 6.91 (1H, d, J = 7.8 Hz, H aromatic).

### 3.3. X-ray Diffraction Analysis

Colorless single crystals were successfully grown from methanol by the slow solvent-evaporation method at room temperature. Single-crystal X-ray data for **1b** were collected on a Bruker D8 Venture diffractometer (Bruker AXS GmbH, Karlsruhe, Germany) using graphite–monochromate Mo Kα radiation (λ = 0.71073 Å) at 293K. Data collection, cell refinement, and data reduction were performed with Bruker Instrument Service v6.2.6 (Bruker AXS GmbH, Karlsruhe, Germany), APEX4^1^, and SAINT^2^, respectively. Absorption correction using equivalent reflections was performed with the SADABS program.^3^ The structure solutions and full-matrix least-squares refinements based on *F*^2^ were performed with the SHELX package^4,5^ and were refined with fixed individual displacement parameters (Uiso\(H) = 1.2 Ueq (Csp^2^ and C_ar_) or 1.5 U_eq_ (Csp^3^)) using a riding model. All nonhydrogen atoms were refined anisotropically. Crystallographic tables were constructed using Olex2.^6^ X-ray crystallographic data in the cif format available at CCDC 2213864 can be obtained free of charge via www.ccdc.cam.ac.uk/data_request/cif, accessed on 26 October 2022.

Crystallographic Data*:* C_15_H_14_BrN_5_O_7_ (M = 456.22 g/mol): triclinic, space group P-1, a = 8.8166(4) Å, b = 9.7965(5) Å, c = 11.7129(5) Å, α = 85.978(2)°, β = 89.864(2)°, γ = 63.810(2)°, V = 905.11(7) Å^3^, Z = 2, T = 273.15 K, μ(Mo Kα) = 2.322 mm^−1^, F (000) = 460.0, crystal size = 0.32 × 0.212 × 0.116 mm^3^, ρ_calc_ = 1.674 g/cm^3^; of the 59,143 reflections measured (4.648° ≤ 2Θ ≤ 51.358°), 3425 were unique (R_int_ = 0.0760, R_sigma_ = 0.0281) and were used in all calculations. The final R_1_ was 0.0417 (I > 2σ(I)), and wR_2_ was 0.1061 (all data).

### 3.4. In Vitro Cytotoxicity Assays

Antiproliferative assays were performed against tumor lines, SNB-19 (astrocytoma), HCT-116 (colon carcinoma—human), PC3 (prostate carcinoma), and HL60 (promyelocytic leukemia), provided by the National Cancer Institute (USA), having been grown in RPMI 1640 medium, supplemented with 10% fetal bovine serum and 1% antibiotics, kept in an oven at 37 °C and an atmosphere containing 5% of CO_2_. The molecular hybrid samples were weighed and diluted in DMSO to final stock concentrations of 40 mM. Cytotoxicity analysis was performed using the MTT method, cells were plated at concentrations of 0.7 × 10^5^ cells/mL (HCT-116), 0.1 × 10^6^ cells/mL (SNB19 and PC3), and 0.3 × 10^6^ cells/ml (HL60). The samples were tested after serial dilution in concentrations from 0.20 to 200 µM, in duplicate in three different experiments. From these solutions, serial dilutions were performed until obtaining a minimum concentration of 0.20 µM for the evaluation of the inhibitory concentration mean (IC_50_). The plates were incubated for 72 h in an oven at 5% CO_2_ at 37 °C. At the end of this, the plates were centrifuged, and the supernatant was removed. Then, 100 µL of the MTT solution (tetrazolium salt) was added, and the plates were incubated for 3 h. The absorbance was read after dissolving the precipitate with 100 µL of pure DMSO in a plate spectrophotometer, at a wavelength of 595 nm. The experiments were analyzed by linear regression using the GraphPad Prism program, version 6.01.

## 4. Conclusions

Spiro compounds **1a**–**j**, **1k,** and **1l** were synthesized with good yields (43–98%) through a microwave-assisted Knoevenagel/Michael/cyclization multicomponent domino methodology, using ethanol as solvent and 1-methylimidazolium chloride ionic liquid as organocatalyst. The reaction conditions considered ideal were determined from a methodological study varying solvent, catalyst, amount of catalyst, temperature, and heating mode. X-ray structure of compounds **1b** was obtained. Unfortunately, the antiproliferative activity of the tested compounds was only moderate and spiro compound **1c** inhibited the four cell lines tested with the lowest IC_50_ values: 52.81 µM for HCT116; 74.40 µM for PC3; 101 µM for SNB19, and 49.72 µM for HL60.

## Figures and Tables

**Table 1:** Results of solvent and catalyst evaluation for the synthesis of **1a**.

Entry	Solvent	Catalyst	Amount of Catalyst (mmol)	Yield (%)
1	CH_3_CN	IL	0.3	81
2	H_2_O	IL	0.3	71
3	Acetone	IL	0.3	28
4	CH_2_Cl_2_	IL	0.3	51
5	Et_2_O	IL	03	39
6	Toluene	IL	0.3	23
7	EtOH	IL	0.3	85
8	EtOH	*p*-TsOH	0.3	80
9	EtOH	Glucose	0.3	39
10	EtOH	Citric acid	0.3	67
11	EtOH	TFA	0.3	80
12	EtOH	IL	0.2	55
13	EtOH	IL	0.1	65
14	EtOH	IL	0.05	58

*p*-TsOH: *p*-toluenesulfonic acid; TFA: trifluoroacetic acid; IL: ionic liquid.

**Table 2:** Results of the evaluation of temperature and heating mode for the synthesis of **1a**.

Entry	Solvent	Heating	Temperature	Time (h)	Yield (%)
1	EtOH	-	0 °C	34	36
2	EtOH	-	r.t.	72	71
3	EtOH	Conventional	Reflux	24	85
4	EtOH	Microwave	80 °C	2	91

**Table 3:** Anti-proliferative activities of selected compounds against HCT116, PC3, HL60, and SNB19 cell lines.

	Cell Lines—IC_50_ (μM)			
Compounds	HCT116	PC3	SNB19	HL60
**1a**	>200	>200	>200	>200
**1b**	89.45	110.7	124.9	77.38
**1c**	52.81	74.40	101.9	49.72
**1d**	>200	>200	>200	>200
**1e**	67.29	117.6	>200	72.26
**1f**	165.8	>200	>200	117.2
**1g**	>200	>200	>200	99.07
**1h**	>200	>200	>200	>200
**1i**	>200	>200	>200	>200
**1j**	>200	>200	>200	>200
**1k**	>200	>200	>200	>200
**1l**	>200	>200	>200	>200

## Data Availability

Not applicable.

## Supplementary Materials

The characterization data for all products can be downloaded at: https://www.mdpi.com/article/10.3390/molecules27228051/s1. These data include: Figure S1. Infrared spectrum of compound **1a**; Figure S2. ^1^H NMR spectrum of compound **1a**; Figure S3. ^13^C NMR spectrum of compound **1a**; Figure S4. ^1^H-^1^H COSY NMR spectrum of compound **1a**; Figure S5. ^1^H-^13^C HSQC NMR spectrum of compound **1a**; Figure S6. ^1^H-^13^C HMBC NMR spectrum of compound **1a** (cnst13 = 3 Hz); Figure S7. ^1^H-^13^C HMBC NMR spectrum of compound **1a** (cnst13 = 8 Hz); Figure S8. Infrared spectrum of compound **1b**; Figure S9. ^1^H NMR spectrum of compound **1b**; Figure S10. ^13^C NMR spectrum of compound **1b**; Figure S11. ^1^H-^1^H COSY NMR spectrum of compound **1b**; Figure S12. ^1^H-^13^C HSQC NMR spectrum of compound **1b**; Figure S13. ^1^H-^13^C HMBC NMR spectrum of compound **1b** (cnst13 = 3 Hz); Figure S14. ^1^H-^13^C HMBC NMR spectrum of compound **1b** (cnst13 = 8 Hz); Figure S15. Mass spectrum of compound **1b**; Figure S16. Infrared spectrum of compound **1c**; Figure S17. ^1^H NMR spectrum of compound **1c**; Figure S18. ^13^C NMR spectrum of compound **1c**; Figure S19. ^1^H-^1^H COSY NMR spectrum of compound **1c**; Figure S20. ^1^H-^13^C HSQC NMR spectrum of compound **1c**; Figure S21. ^1^H-^13^C HMBC NMR spectrum of compound **1c** (cnst13 = 3 Hz); Figure S22. ^1^H-^13^C HMBC NMR spectrum of compound **1c** (cnst13 = 8 Hz); Figure S23. Mass spectrum of compound **1c**; Figure S24. Infrared spectrum of compound **1d**; Figure S25. ^1^H NMR spectrum of compound **1d**; Figure S26. ^13^C NMR spectrum of compound **1d**; Figure S27. ^1^H-^1^H COSY NMR spectrum of compound **1d**; Figure S28. ^1^H-^13^C HSQC NMR spectrum of compound **1d**; Figure S29. ^1^H-^13^C HMBC NMR spectrum of compound **1d** (cnst13 = 3 Hz); Figure S30. ^1^H-^13^C HMBC NMR spectrum of compound **1d** (cnst13 = 8 Hz); Figure S31. Mass spectrum of compound **1d**; Figure S32. Infrared spectrum of compound **1e**; Figure S33. ^1^H NMR spectrum of compound **1e**; Figure S34. ^13^C NMR spectrum of compound **1e**; Figure S35. ^1^H-^1^H COSY NMR spectrum of compound **1e**; Figure S36. ^1^H-^13^C HSQC NMR spectrum of compound **1e**; Figure S37. ^1^H-^13^C HMBC NMR spectrum of compound **1e** (cnst13 = 3 Hz); Figure S38. ^1^H-^13^C HMBC NMR spectrum of compound **1e** (cnst13 = 8 Hz); Figure S39. Mass spectrum of compound **1e**; Figure S40. Infrared spectrum of compound **1f**; Figure S41. ^1^H NMR spectrum of compound **1f**; Figure S42. ^13^C NMR spectrum of compound **1f**; Figure S43. ^1^H-^1^H COSY NMR spectrum of compound **1f**; Figure S44. ^1^H-^13^C HSQC NMR spectrum of compound **1f**; Figure S45. ^1^H-^13^C HMBC NMR spectrum of compound **1f** (cnst13 = 3 Hz); Figure S46. ^1^H-^13^C HMBC NMR spectrum of compound **1f** (cnst13 = 8 Hz); Figure S47. Mass spectrum of compound **1f**; Figure S48. Infrared spectrum of compound **1g**; Figure S49. ^1^H NMR spectrum of compound **1g**; Figure S50. ^13^C NMR spectrum of compound **1g**; Figure S51. ^1^H-^1^H COSY NMR spectrum of compound **1g**; Figure S52. ^1^H-^13^C HSQC NMR spectrum of compound **1g**; Figure S53. ^1^H-^13^C HMBC NMR spectrum of compound **1g** (cnst13 = 3 Hz); Figure S54. ^1^H-^13^C HMBC NMR spectrum of compound **1g** (cnst13 = 8 Hz); Figure S55. Mass spectrum of compound **1g**; Figure S56. Infrared spectrum of compound **1h**; Figure S57. ^1^H NMR spectrum of compound **1h**; Figure S58. ^13^C NMR spectrum of compound **1h**; Figure S59. ^1^H-^1^H COSY NMR spectrum of compound **1h**; Figure S60. ^1^H-^13^C HSQC NMR spectrum of compound **1h**; Figure S61. ^1^H-^13^C HMBC NMR spectrum of compound **1h** (cnst13 = 3 Hz); Figure S62. ^1^H-^13^C HMBC NMR spectrum of compound **1h** (cnst13 = 8 Hz); Figure S63. Mass spectrum of compound **1h**; Figure S64. Infrared spectrum of compound **1i**; Figure S65. ^1^H NMR spectrum of compound **1i**; Figure S66. ^13^C NMR spectrum of compound **1i**; Figure S67. ^1^H-^1^H COSY NMR spectrum of compound **1i**; Figure S68. ^1^H-^13^C HSQC NMR spectrum of compound **1i**; Figure S69. ^1^H-^13^C HMBC NMR spectrum of compound **1i** (cnst13 = 3 Hz); Figure S70. ^1^H-^13^C HMBC NMR spectrum of compound **1i** (cnst13 = 8 Hz); Figure S71. Mass spectrum of compound **1i**; Figure S72. Infrared spectrum of compound **1j**; Figure S73. ^1^H NMR spectrum of compound **1j**; Figure S74. ^13^C NMR spectrum of compound **1j**; Figure S75. ^1^H-^1^H COSY NMR spectrum of compound **1j**; Figure S76. ^1^H-^13^C HSQC NMR spectrum of compound **1j**; Figure S77. ^1^H-^13^C HMBC NMR spectrum of compound **1j** (cnst13 = 3 Hz); Figure S78. ^1^H-^13^C HMBC NMR spectrum of compound **1j** (cnst13 = 8 Hz); Figure S79. Mass spectrum of compound **1j**; Figure S80. Infrared spectrum of compound **1k**; Figure S81. ^1^H NMR spectrum of compound **1k**; Figure S82. ^13^C NMR spectrum of compound **1k**; Figure S83. ^1^H-^1^H COSY NMR spectrum of compound **1k**; Figure S84. ^1^H-^13^C HSQC NMR spectrum of compound **1k**; Figure S85. ^1^H-^13^C HMBC NMR spectrum of compound **1k** (cnst13 = 3 Hz); Figure S86. ^1^H-^13^C HMBC NMR spectrum of compound **1k** (cnst13 = 8 Hz); Figure S87. Mass spectrum of compound **1k**; Figure S88. Infrared spectrum of compound **1l**; Figure S89. ^1^H NMR spectrum of compound **1l**; Figure S90. ^13^C NMR spectrum of compound **1l**; Figure S91. ^1^H-^1^H COSY NMR spectrum of compound **1l**; Figure S92. ^1^H-^13^C HSQC NMR spectrum of compound **1l**; Figure S93. ^1^H-^13^C HMBC NMR spectrum of compound **1l** (cnst13 = 3 Hz); Figure S94. ^1^H-^13^C HMBC NMR spectrum of compound **1l** (cnst13 = 8 Hz); Figure S95. Mass spectrum of compound **1l**; Figure S96. Infrared spectrum of compound **6**; Figure S97. ^1^H NMR spectrum of compound **6**; Figure S98. Infrared spectrum of compound **10**; Figure S99. ^1^H NMR spectrum of compound **10**.
