# Supplementary material for: Green Synthesis of Spiro Compounds with Potential Anticancer Activity through Knoevenagel/Michael/Cyclization Multicomponent Domino Reactions Organocatalyzed by Ionic Liquid and Microwave-Assisted
Source: Molecules. 2022 Nov 19;27(22):8051. doi: 10.3390/molecules27228051 (PMC9697156; doi:10.3390/molecules27228051)
Supplement: Supplementary file 1 [file molecules-27-08051-s001.zip › molecules-2025146-supplementary.pdf]

Supplementary Material

Green synthesis of spiro compounds through Knoevenagel/Michael/cyclization multicomponent domino reactions organocatalyzed by ionic liquid and micro-wave-assisted

Regina Westphal [1], Eclair Venturini Filho [1], Laiza Bruzadelle Loureiro [2], Fabrizio Medici [3], Maurizio Benaglia [3], Cláudio Francisco Tormena [2] e Sandro José Greco [1,*]

[1] Chemistry Department, Federal University of Espírito Santo, Fernando Ferrari Avenue 514, Vitória, ES, Brazil

[2] Institute of Chemistry, University of Campinas, Josué de Castro Street, Campinas, SP, Brazil

[3] Chemistry Department, University of Milan, Via Golgi 19, Milan, Italy

* Correspondence: grecosj@outlook.com

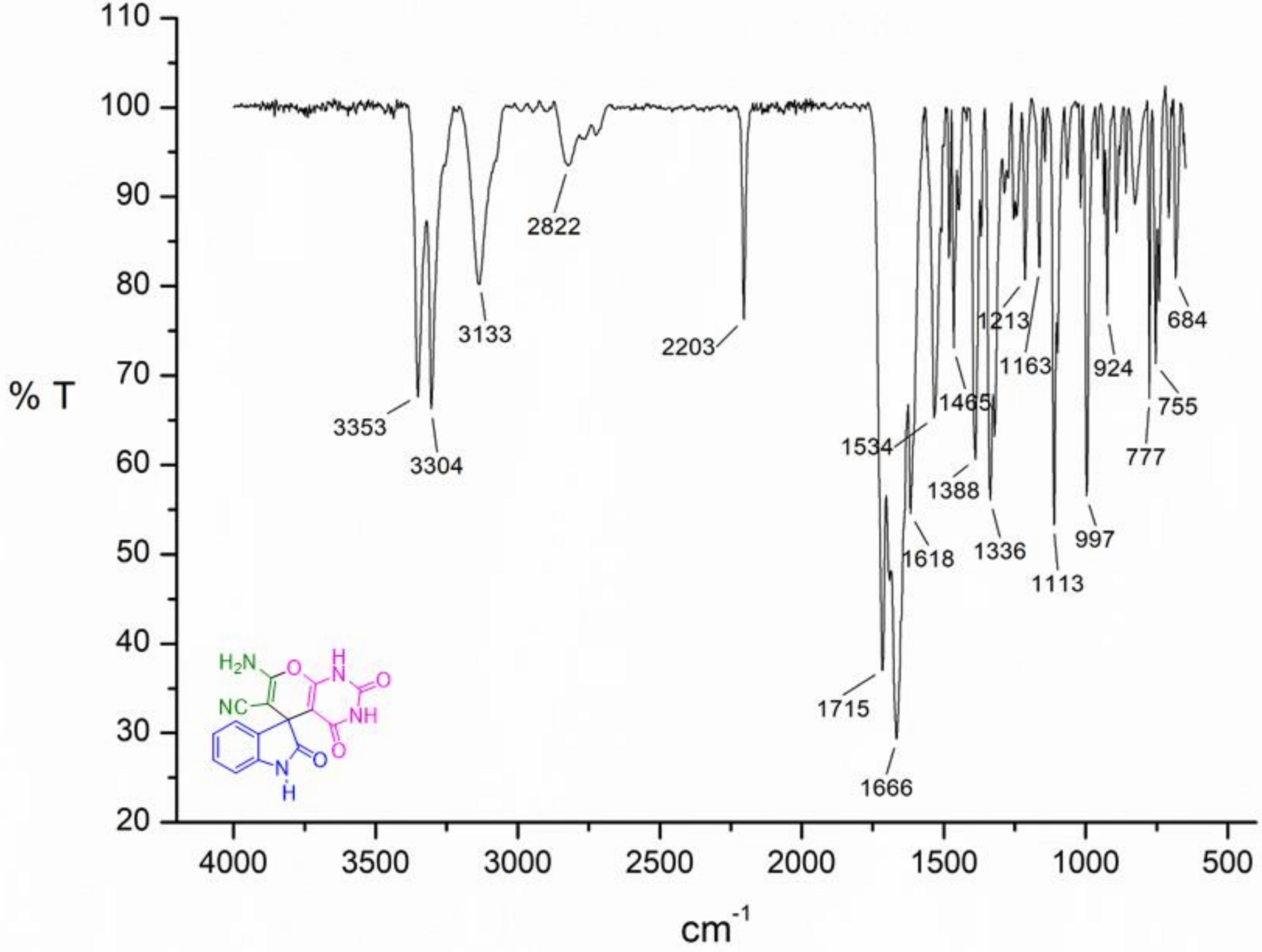

Figure S1. Infrared spectrum of compound **1a**.

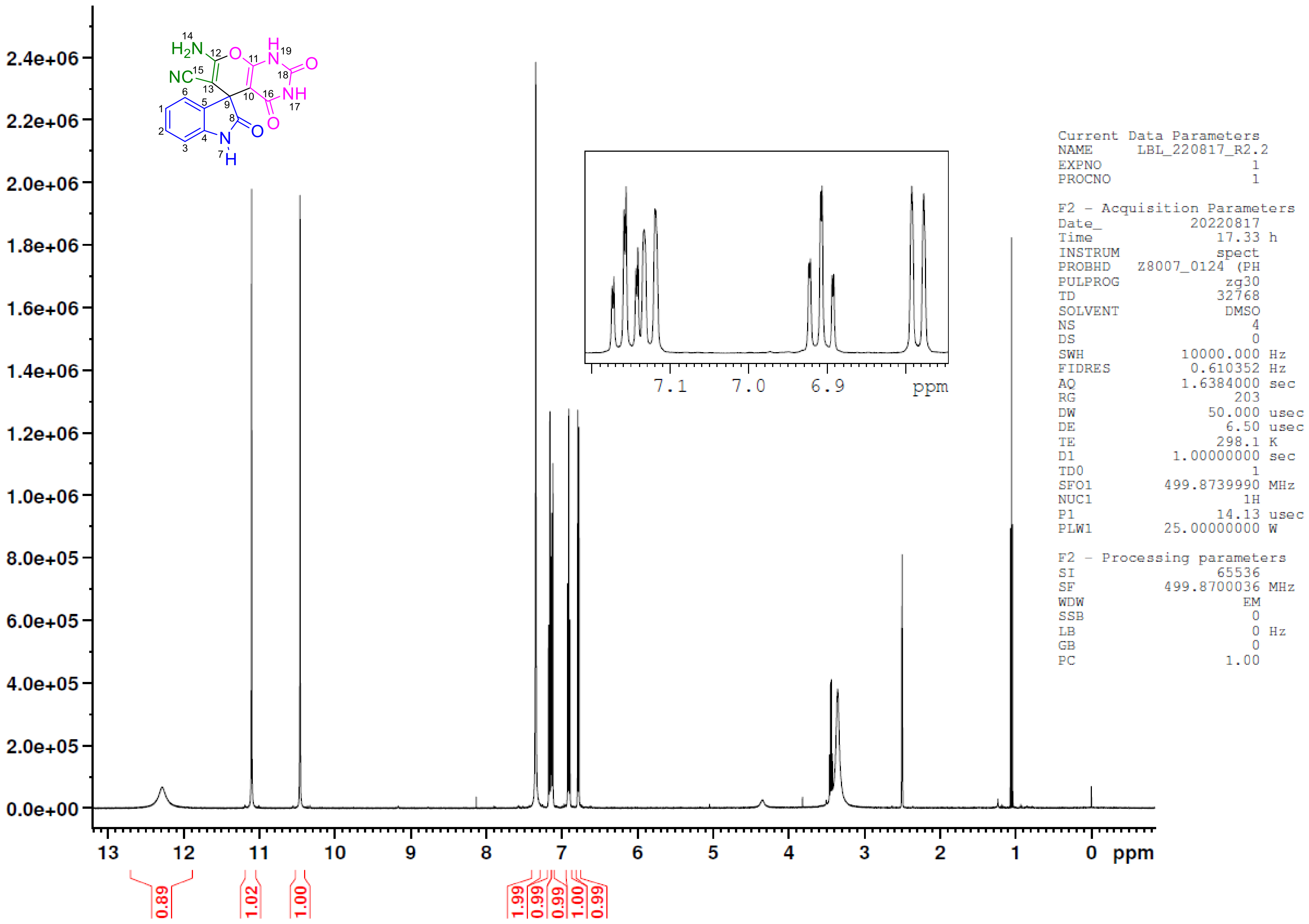

Figure S2. ^{1}H NMR spectrum of compound **1a**.

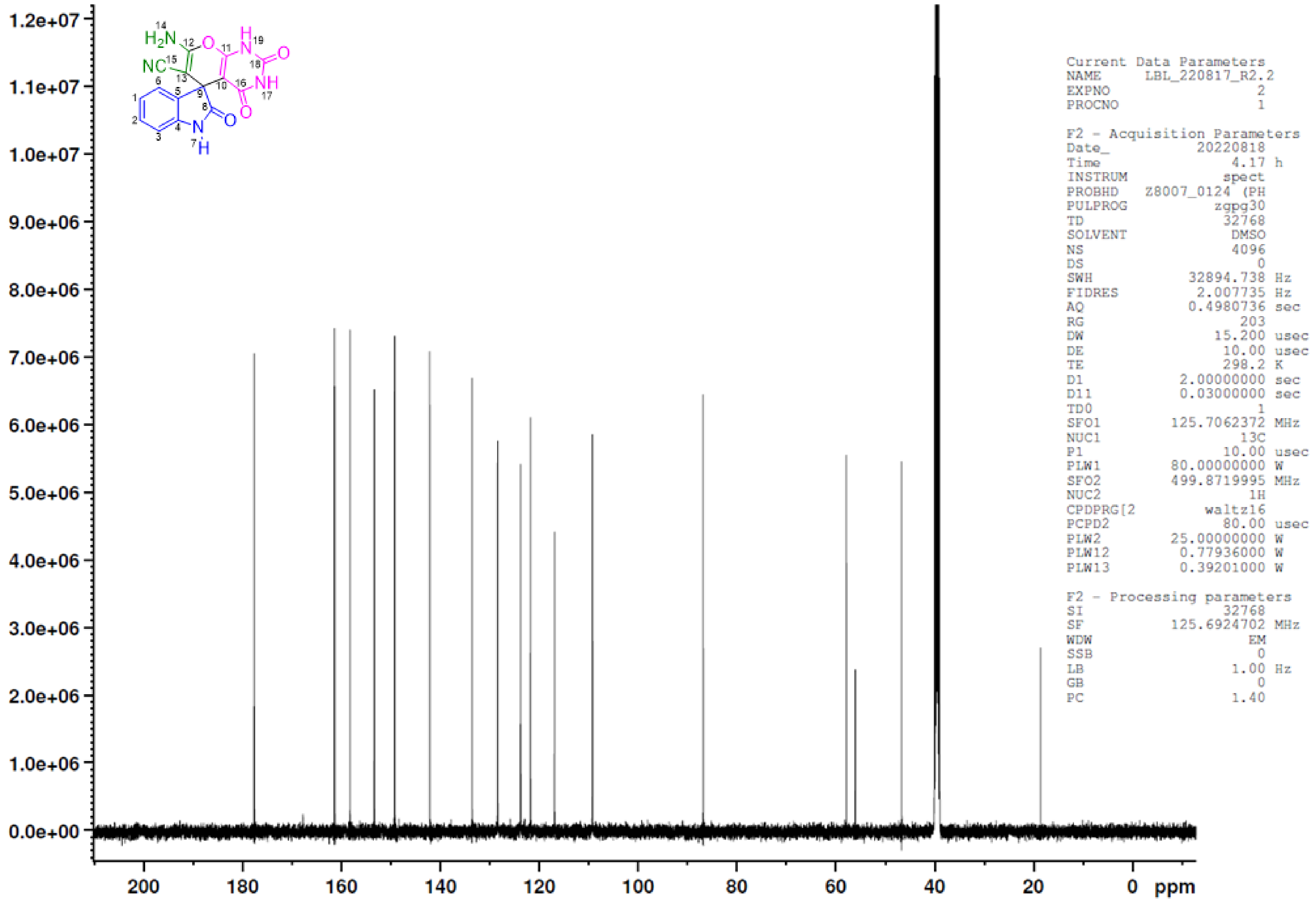

Figure S3. ^{13}C NMR spectrum of compound **1a**.

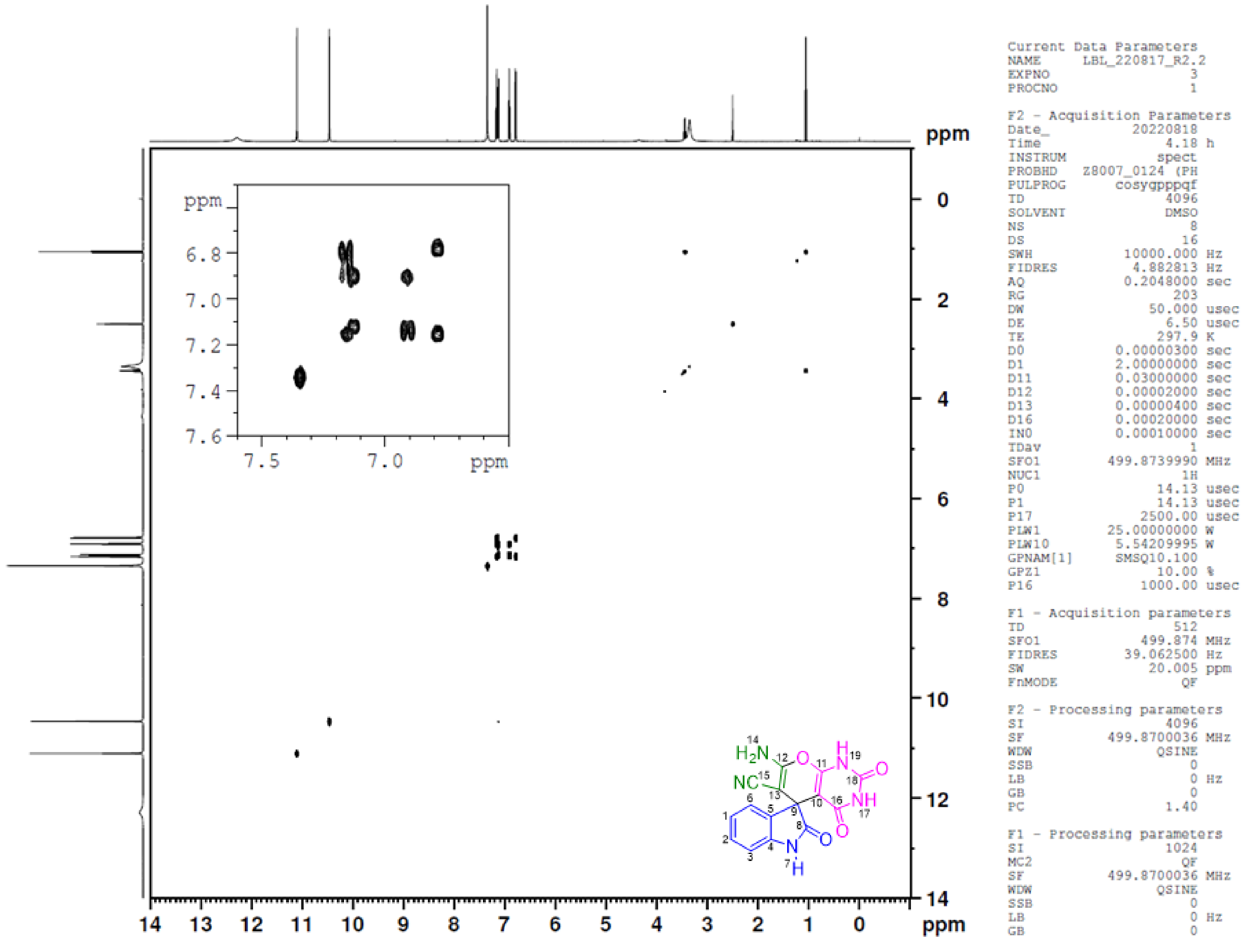

Figure S4. ^1H-^1H COSY NMR spectrum of compound **1a**.

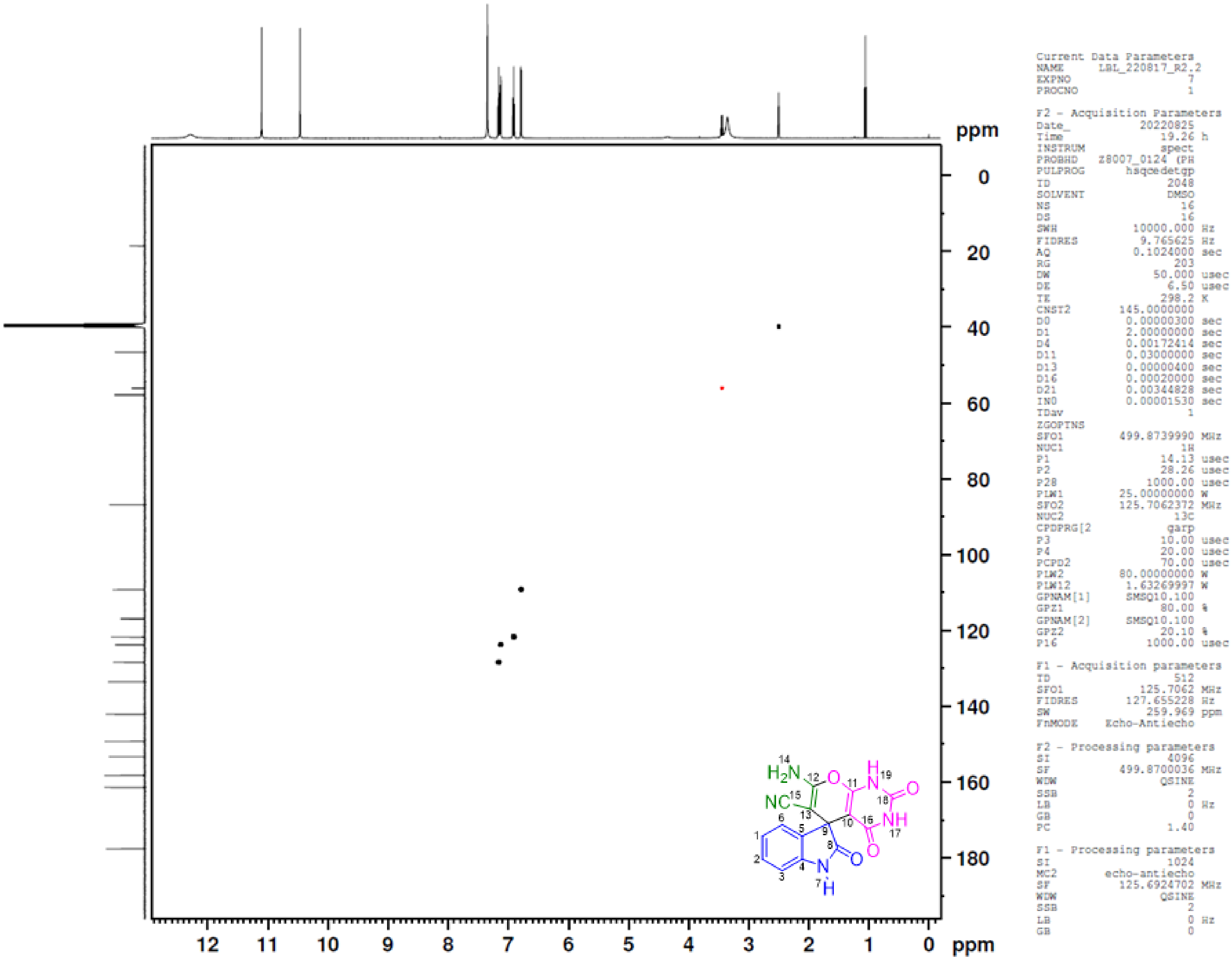

Figure S5. ^{1}H-^{13}C HSQC NMR spectrum of compound **1a**.

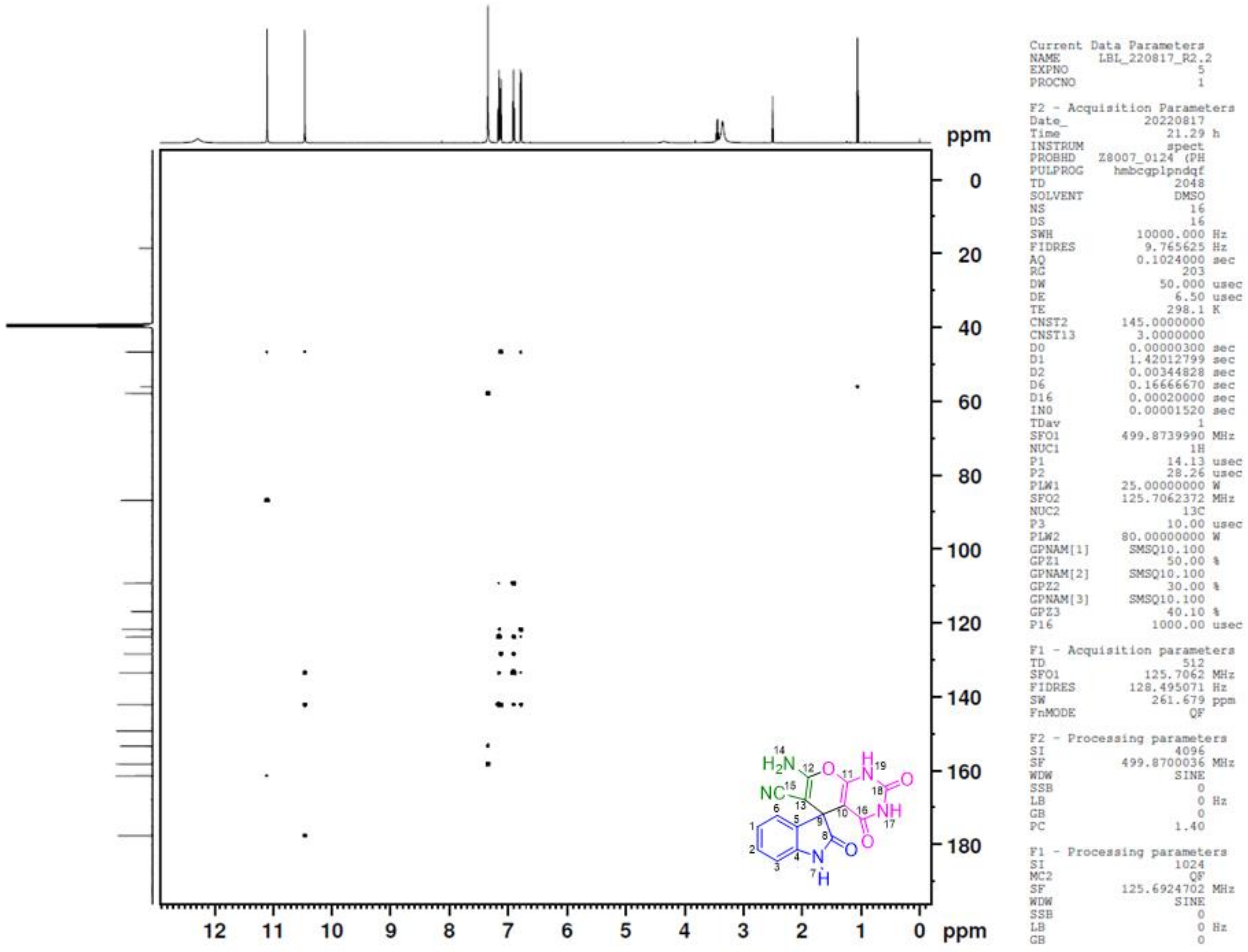

Figure S6. ^{1}H-^{13}C HMBC NMR spectrum of compound **1a** (cnst13 = 3 Hz).

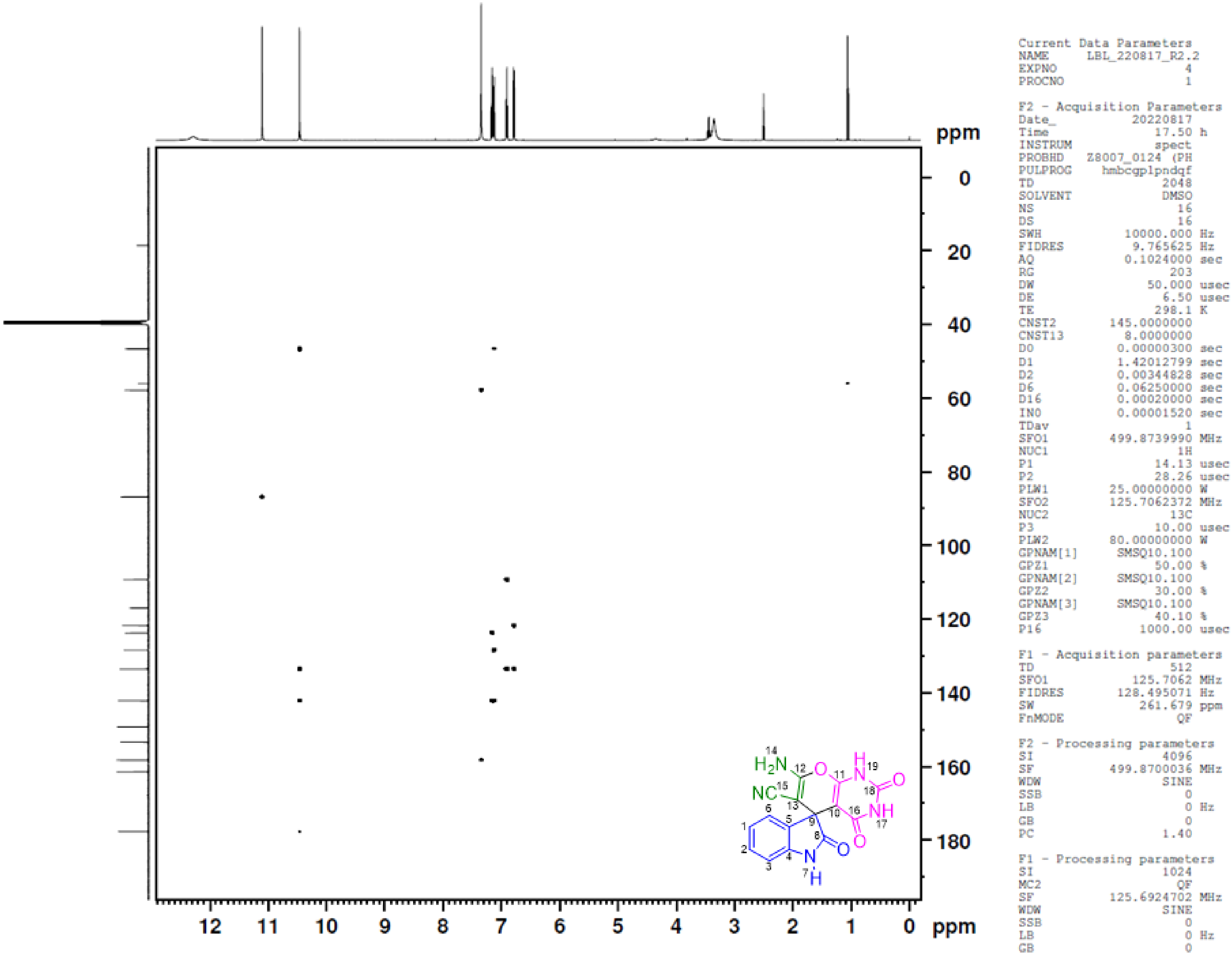

Figure S7. ^{1}H-^{13}C HMBC NMR spectrum of compound **1a** (cnst13 = 8 Hz).

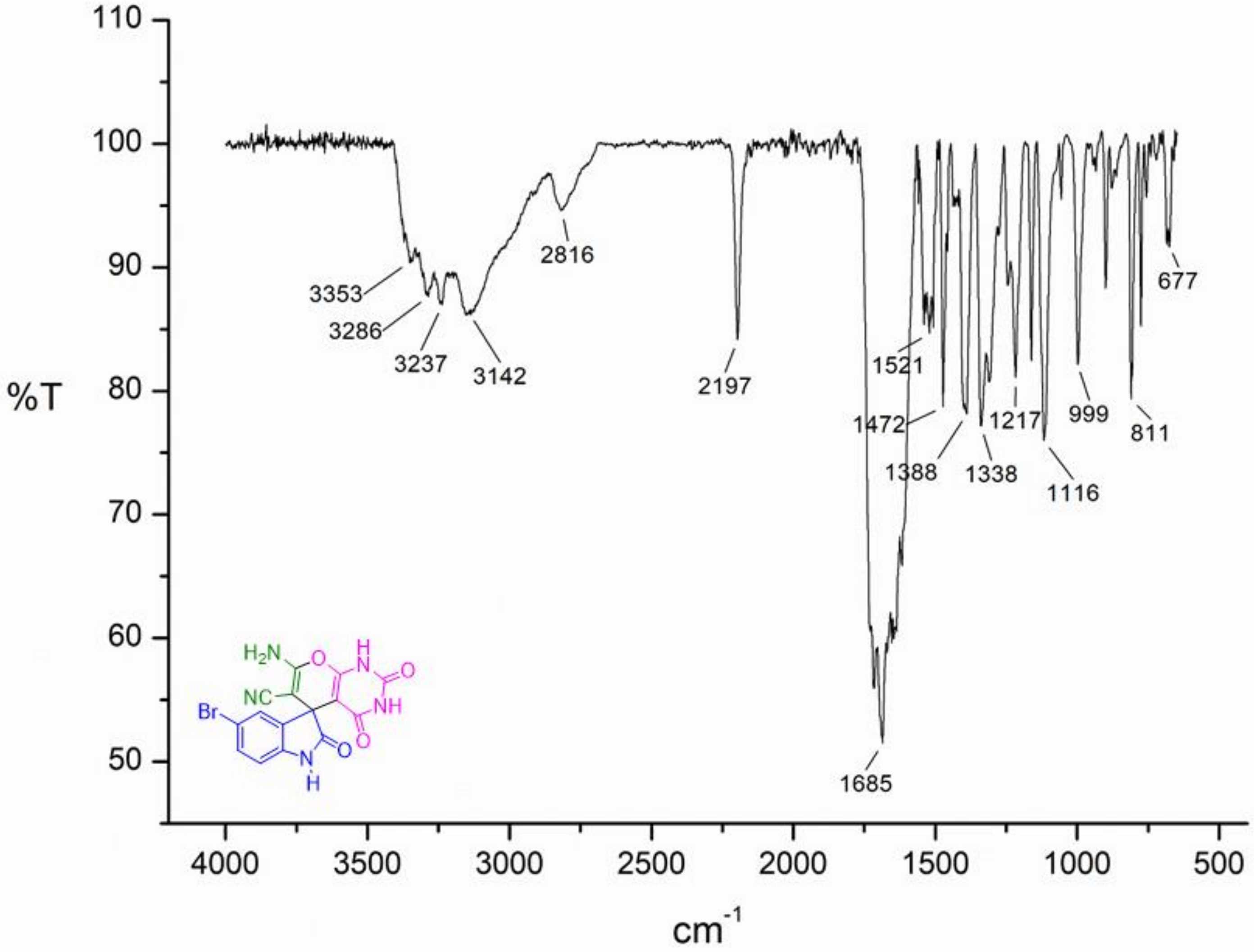

Figure S8. Infrared spectrum of compound **1b**.

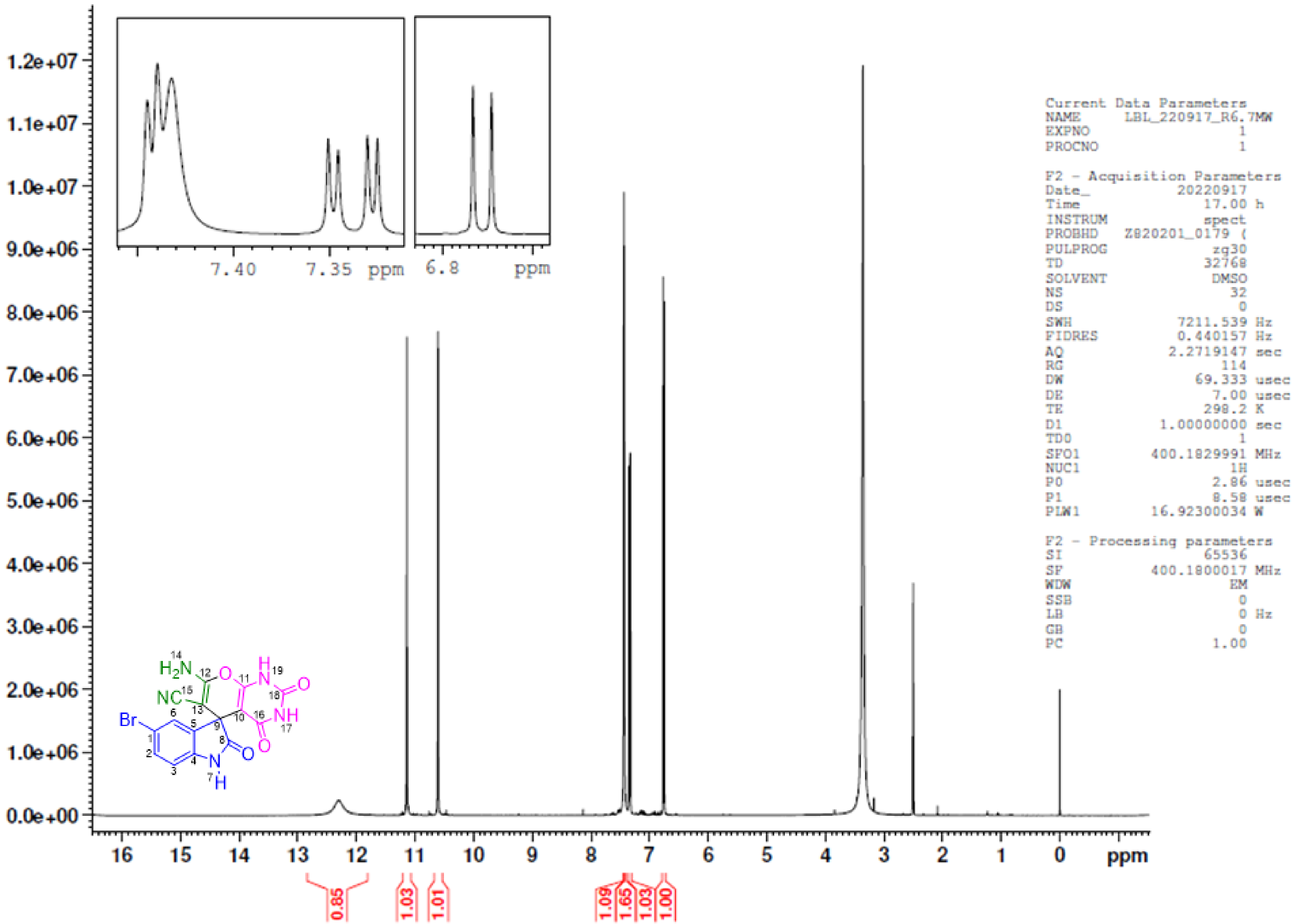

Figure S9. ^{1}H NMR spectrum of compound **1b**.

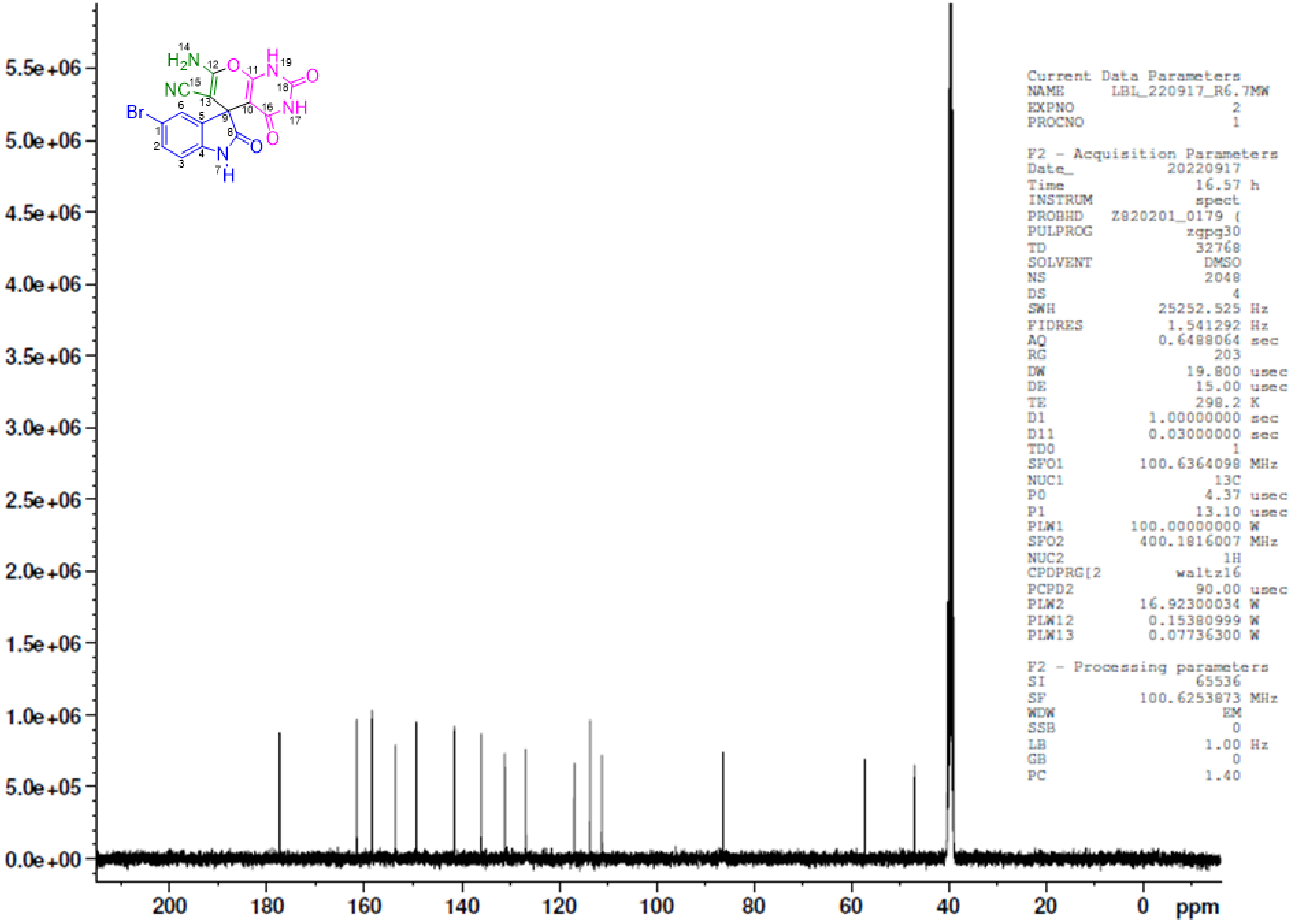

Figure S10. ^{13}C NMR spectrum of compound **1b**.

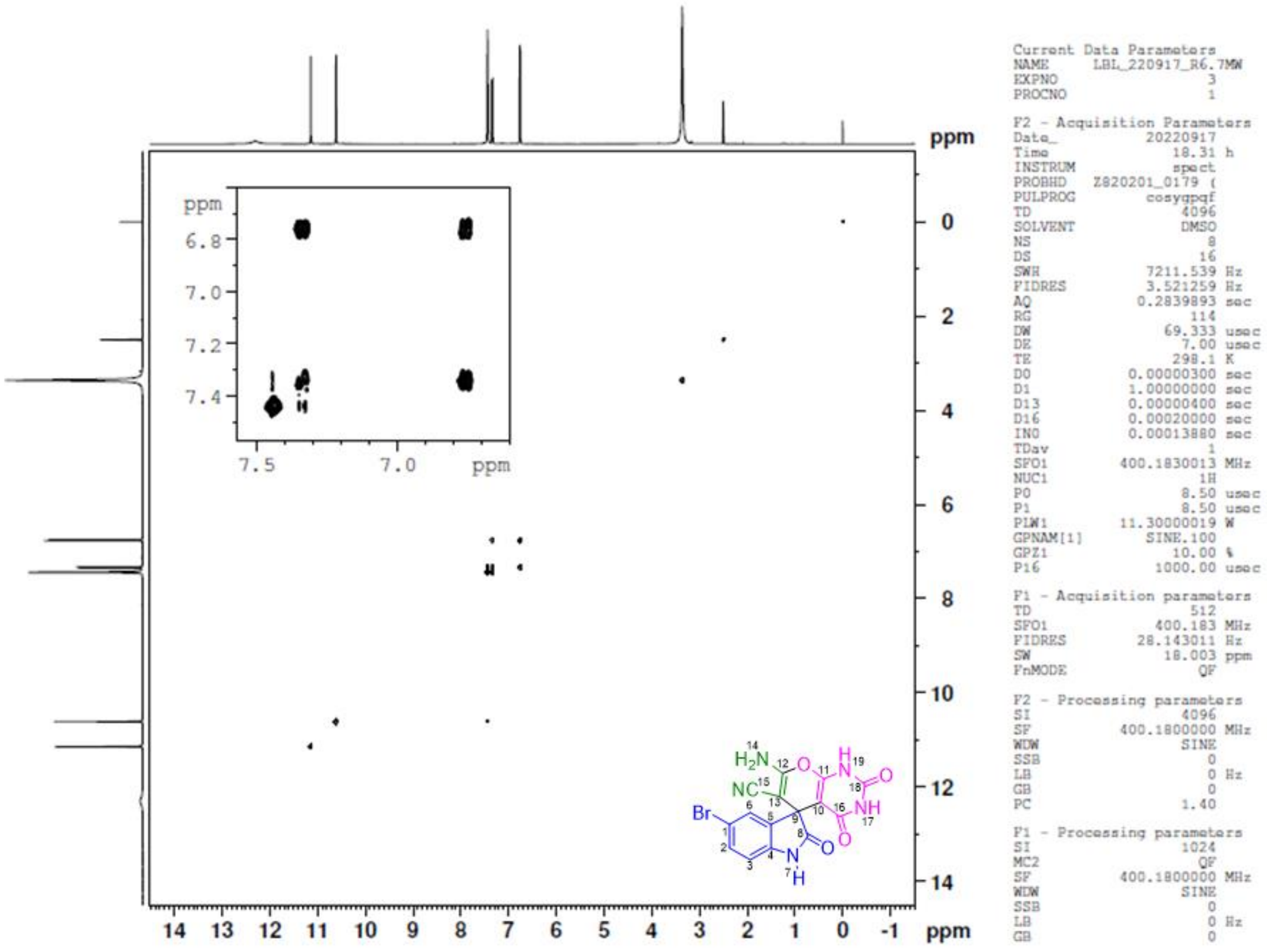

Figure S11. ^1H-^1H COSY NMR spectrum of compound **1b**.

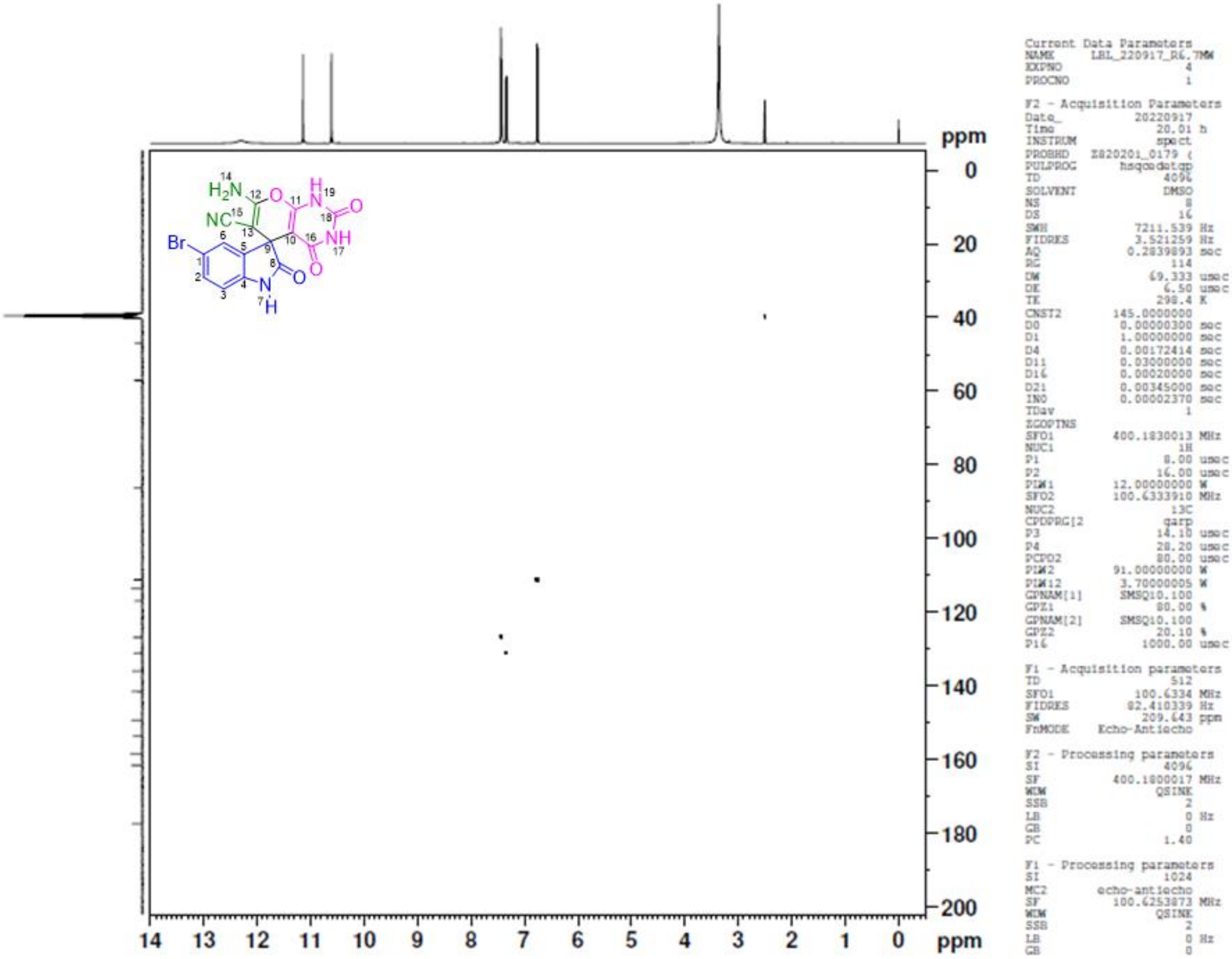

Figure S12. ^{1}H-^{13}C HSQC NMR spectrum of compound **1b**.

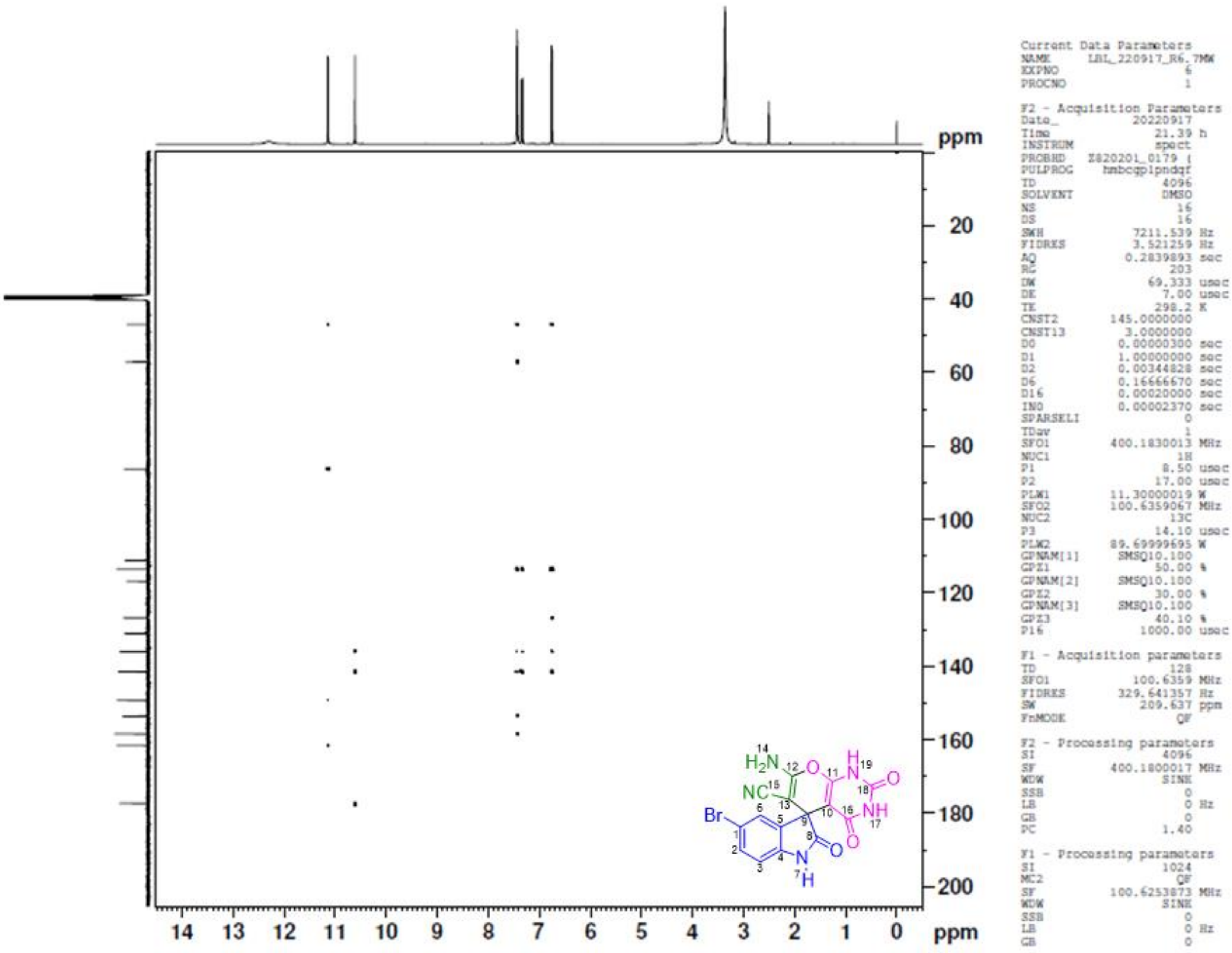

Figure S13. ^{1}H-^{13}C HMBC NMR spectrum of compound **1b** (cnst13 = 3 Hz).

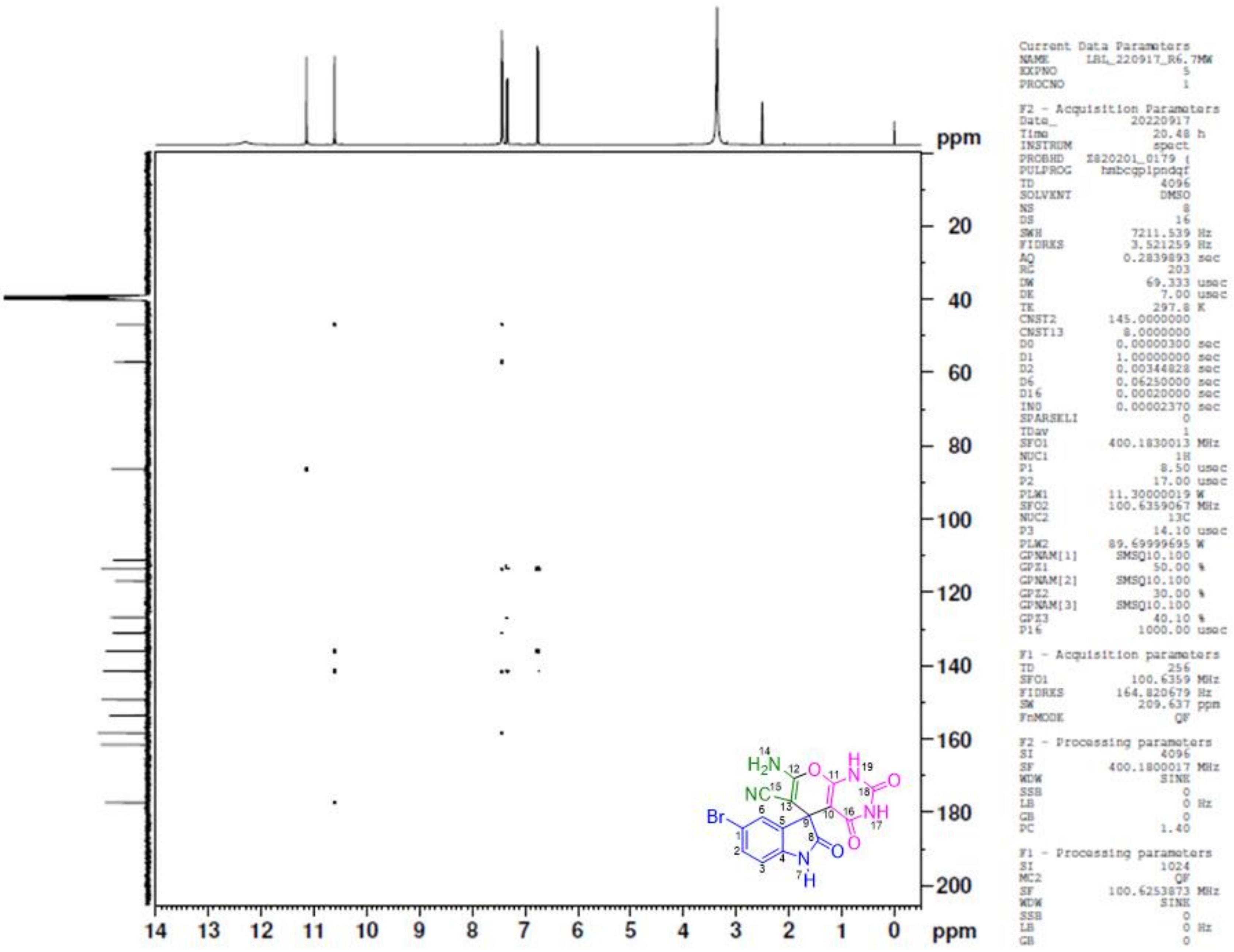

Figure S14. ^1H-^{13}C HMBC NMR spectrum of compound **1b** (cnst13 = 8 Hz).

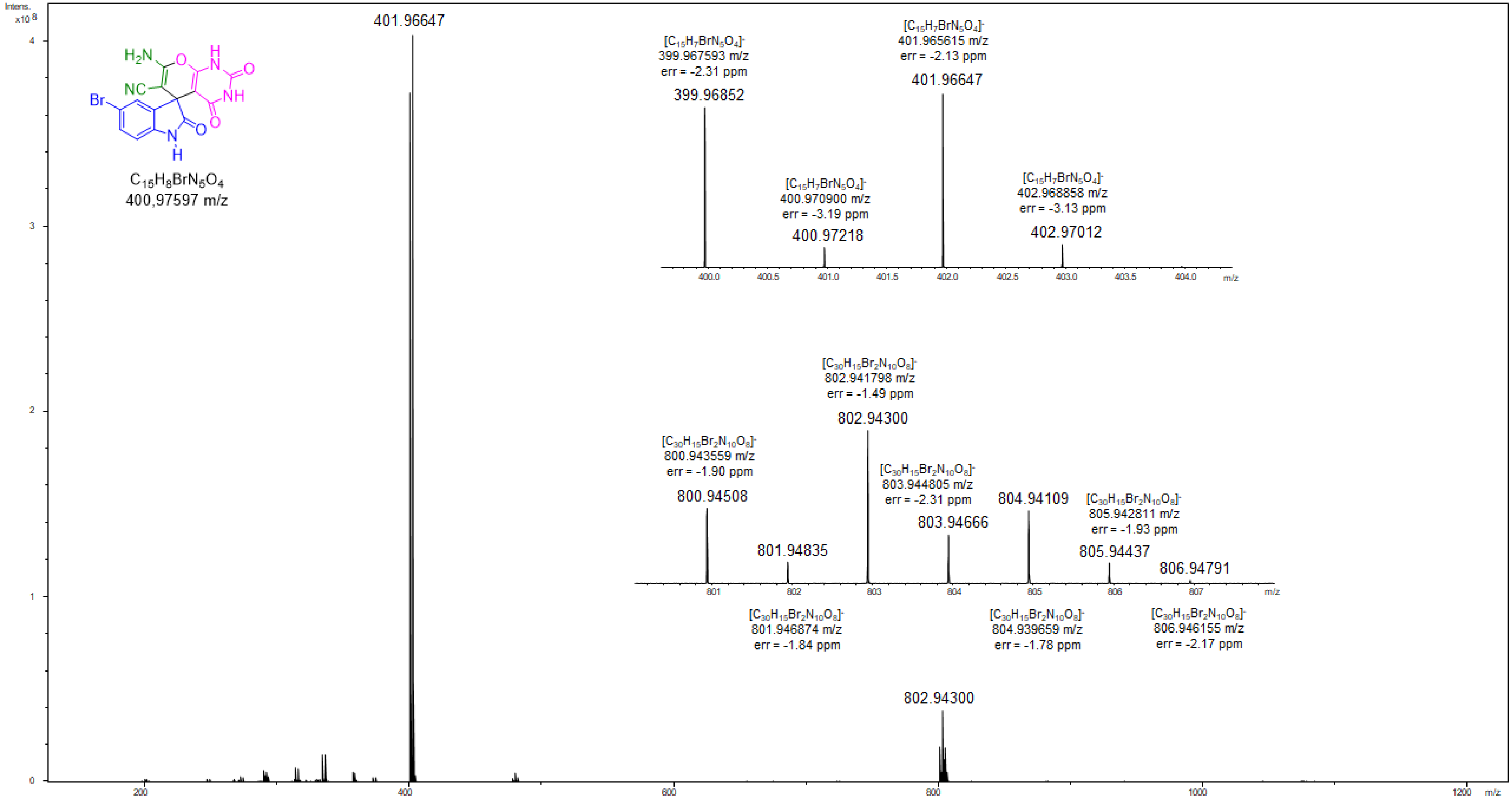

Figure S15. Mass spectrum of compound **1b**.

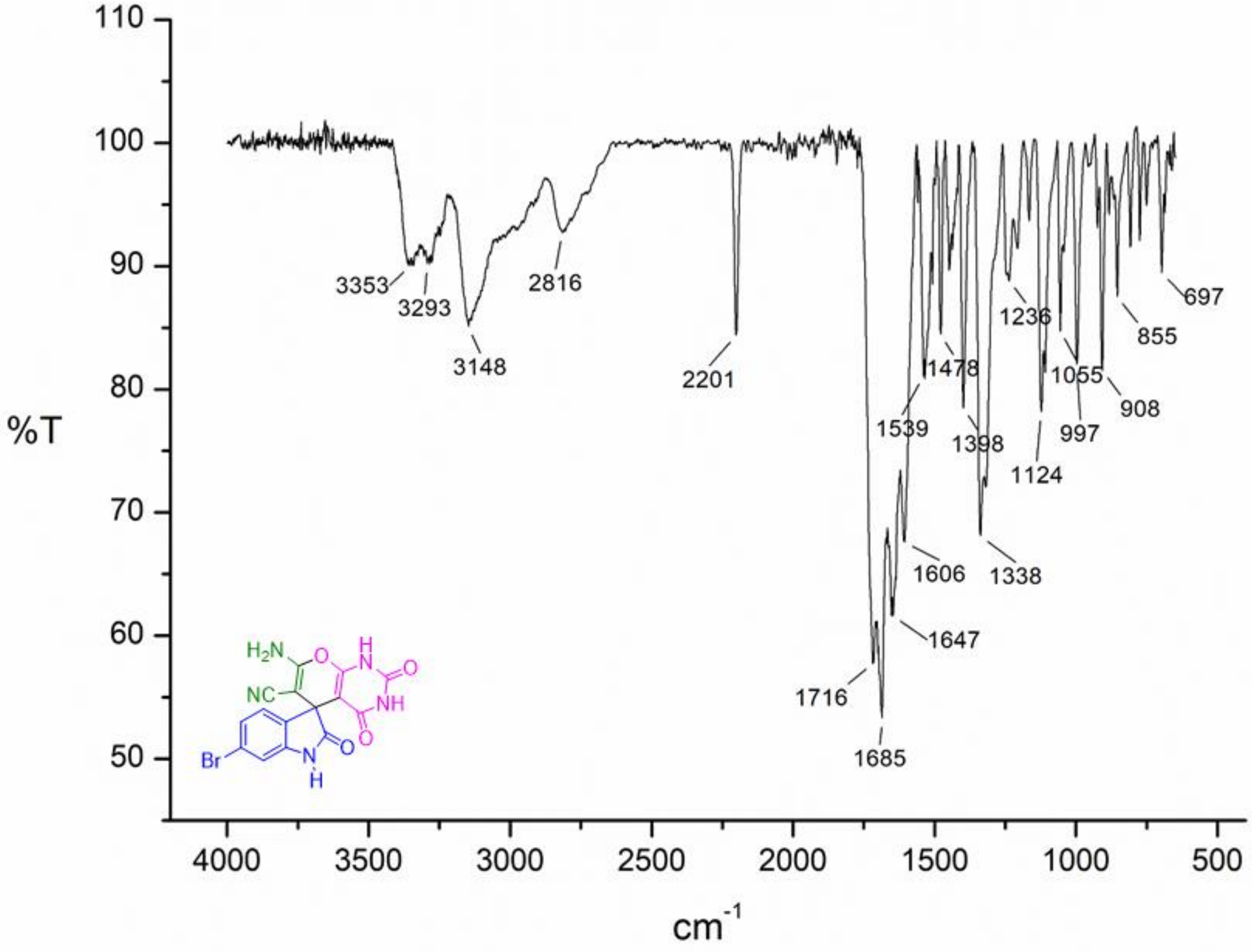

Figure S16. Infrared spectrum of compound **1c**.

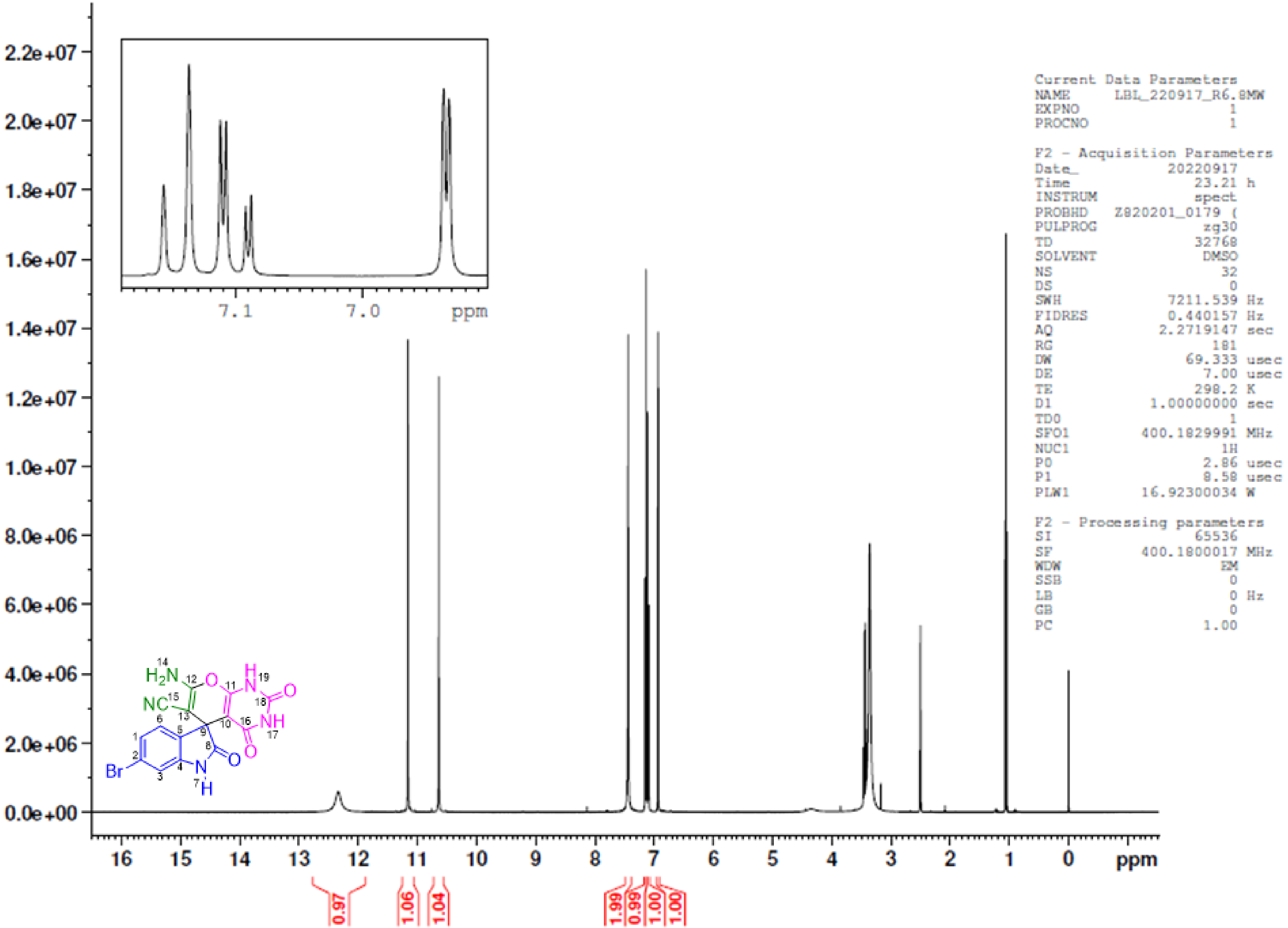

Figure S17. ^{1}H NMR spectrum of compound **1c**.

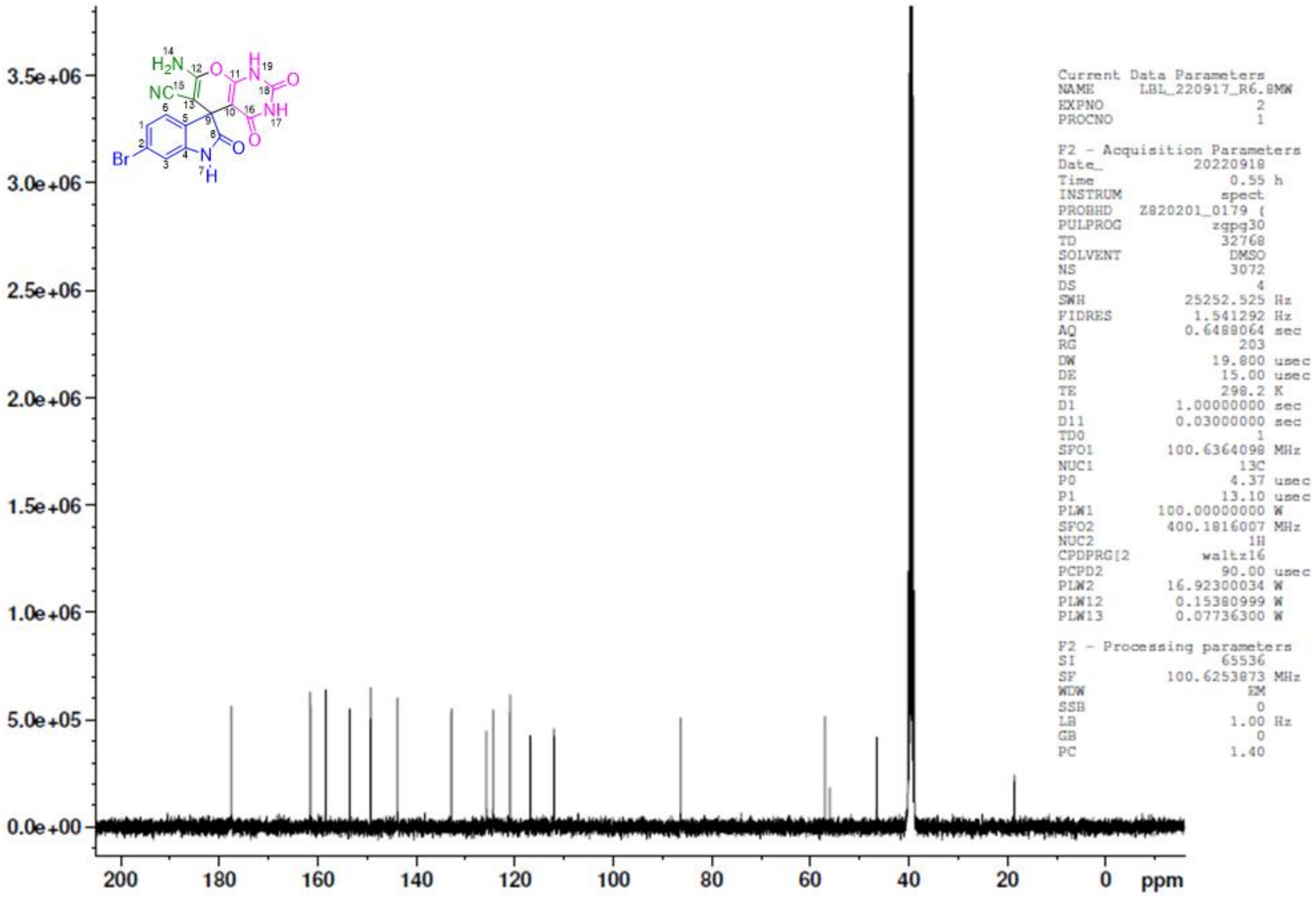

Figure S18. ^{13}C NMR spectrum of compound **1c**.

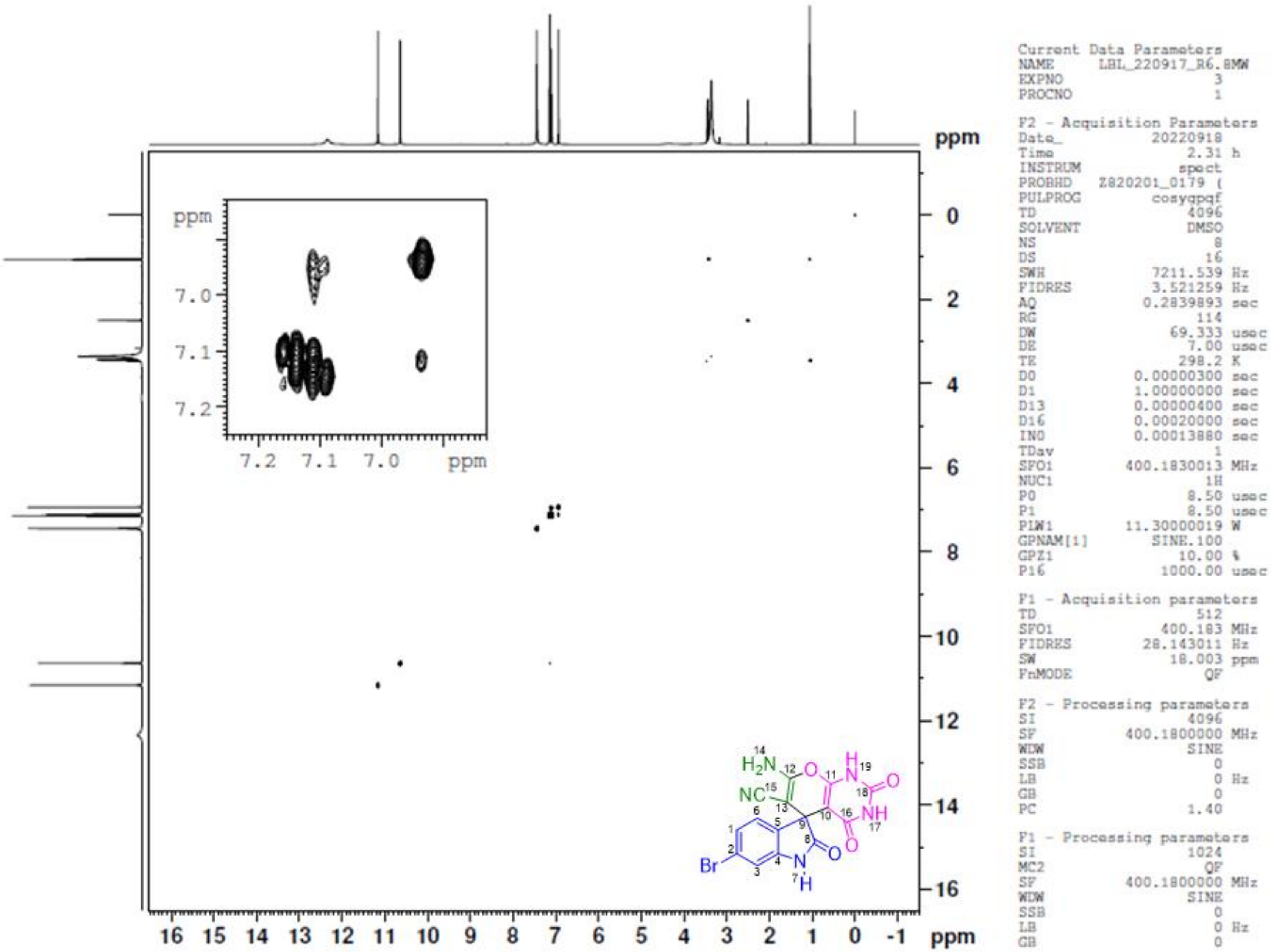

Figure S19. ^1H-^1H COSY NMR spectrum of compound **1c**.

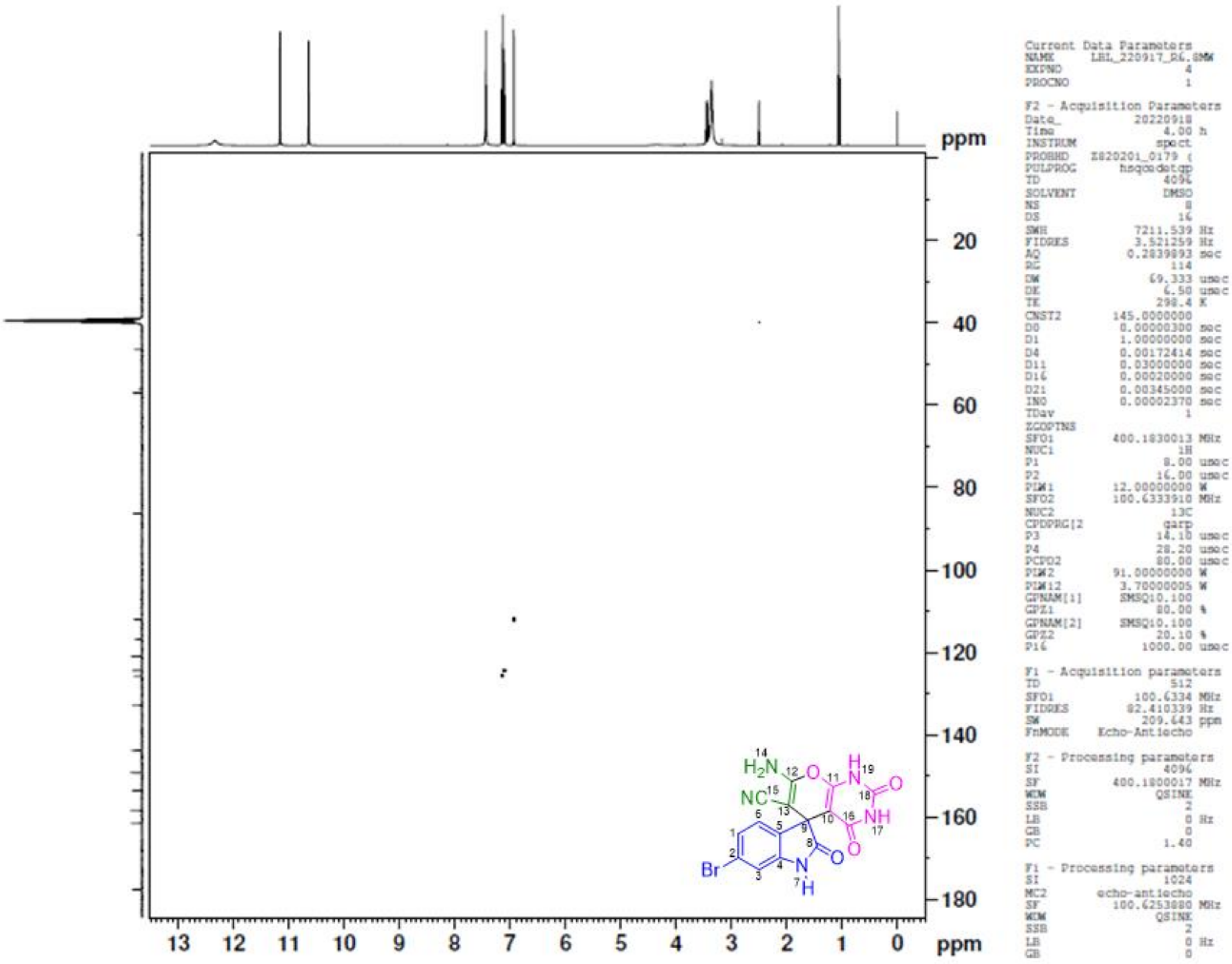

Figure S20. ^{1}H-^{13}C HSQC NMR spectrum of compound **1c**.

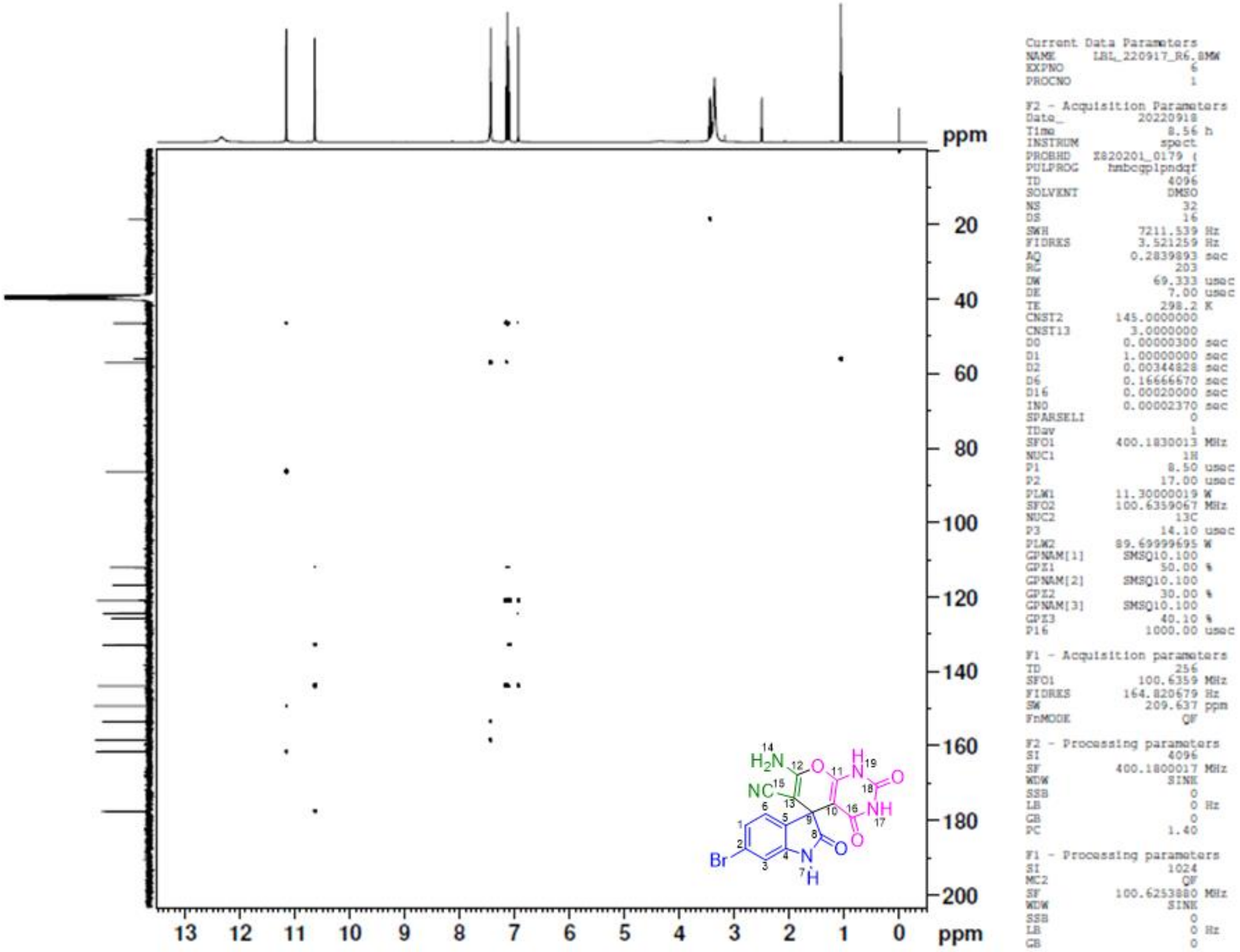

Figure S21. ^{1}H-^{13}C HMBC NMR spectrum of compound **1c** (cnst13 = 3 Hz).

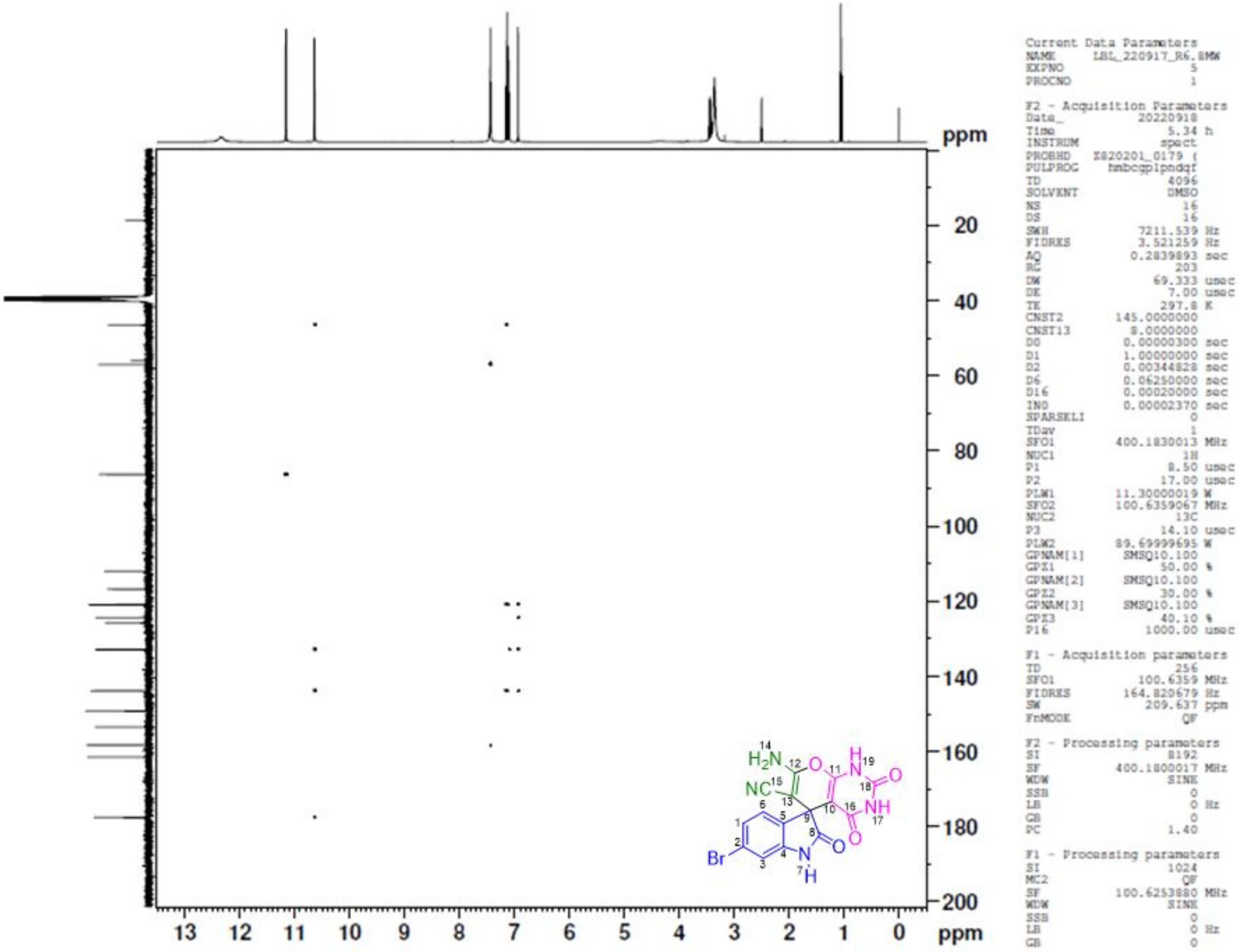

Figure S22. ^{1}H-^{13}C HMBC NMR spectrum of compound **1c** (cnst13 = 8 Hz).

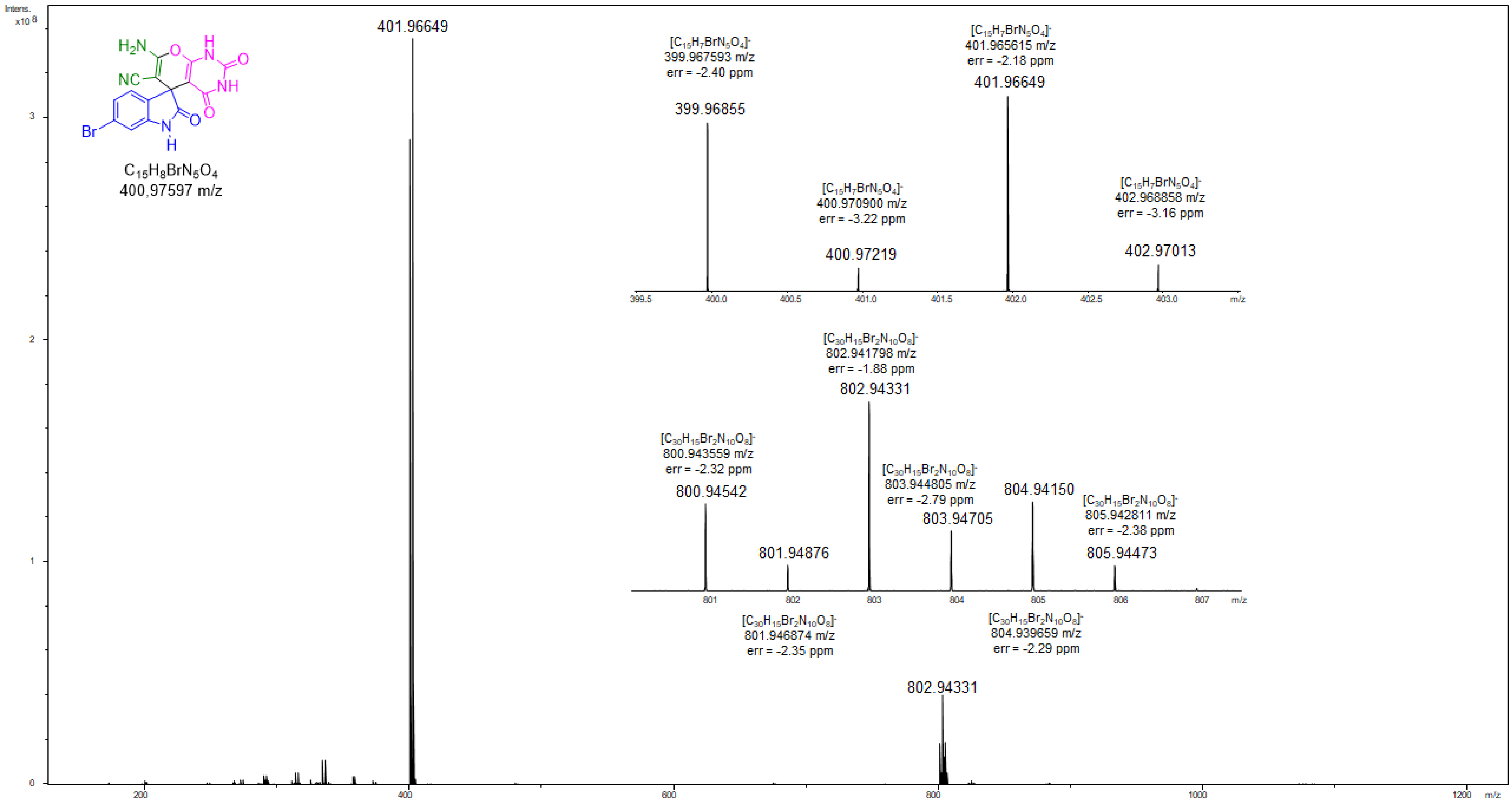

Figure S23. Mass spectrum of compound **1c**.

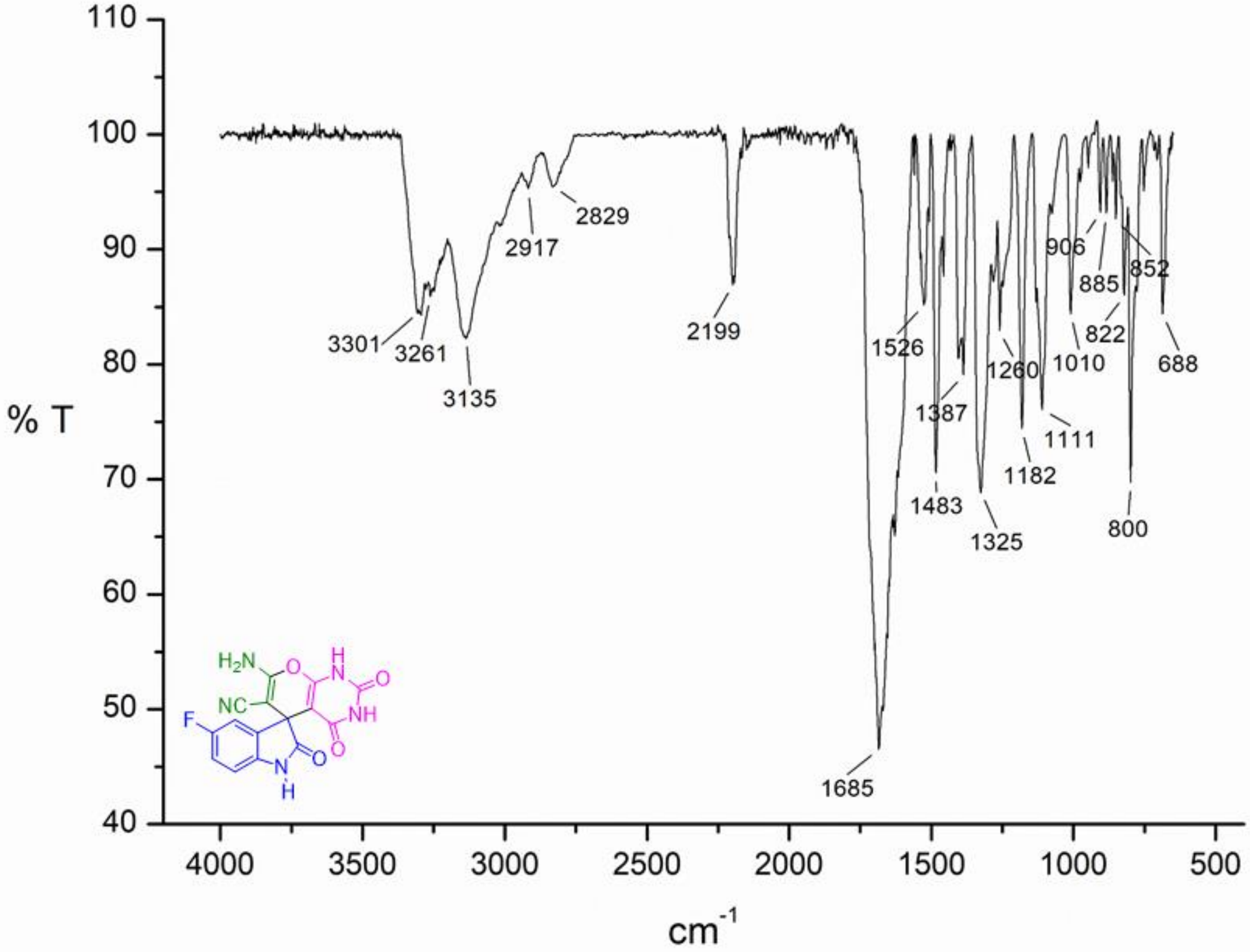

Figure S24. Infrared spectrum of compound **1d**.

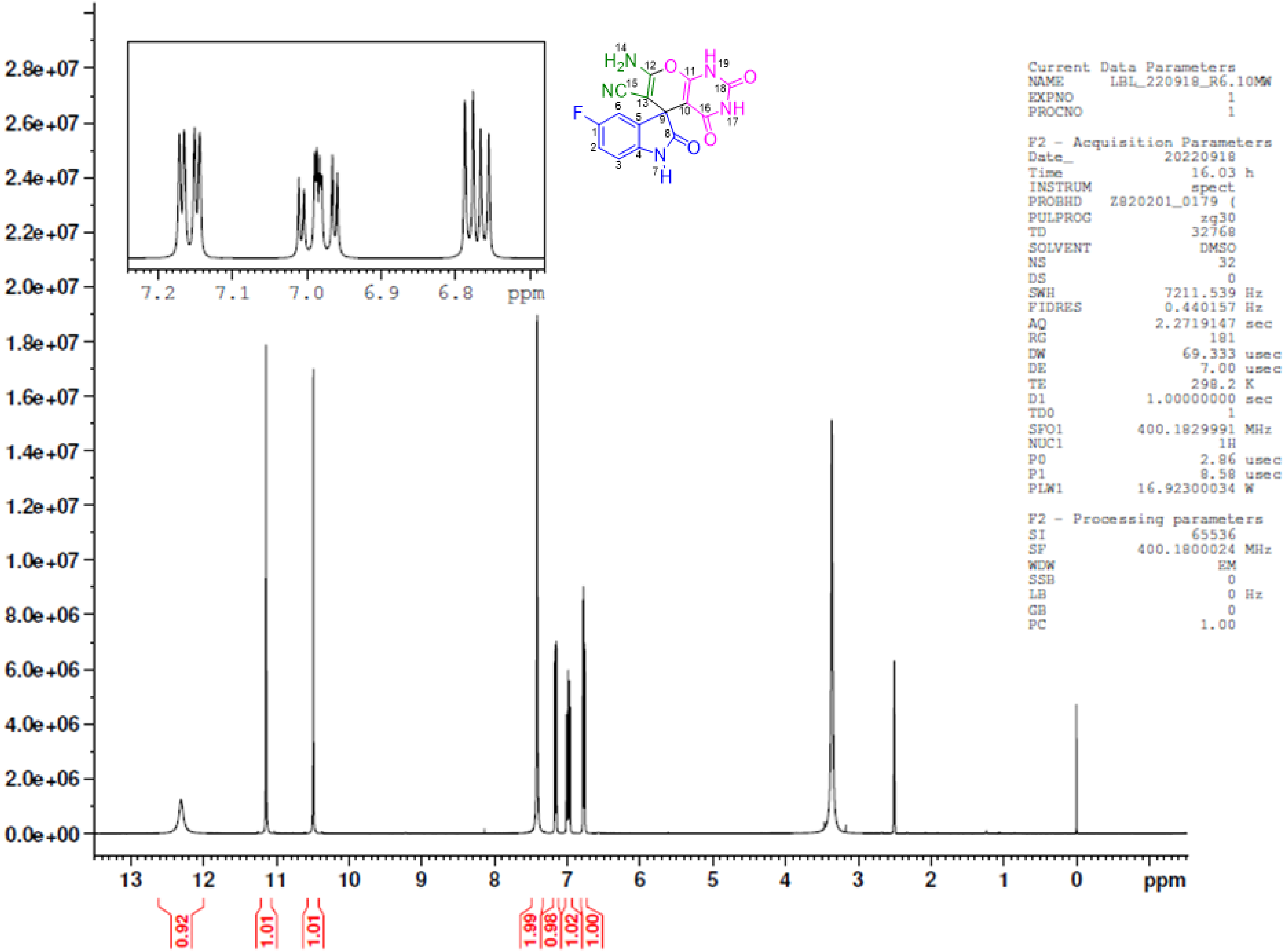

Figure S25. ^{1}H NMR spectrum of compound **1d**.

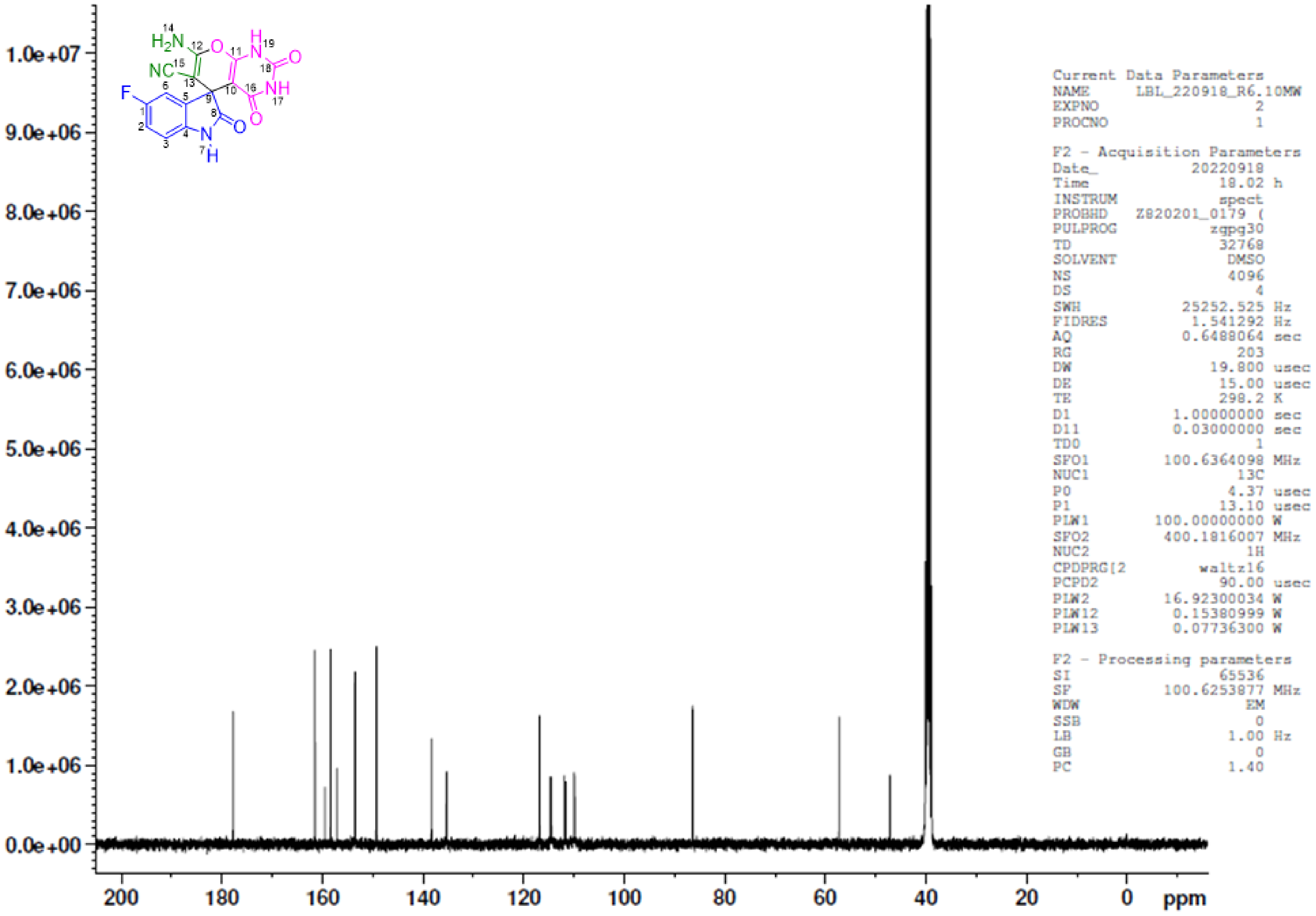

Figure S26. ^{13}C NMR spectrum of compound **1d**.

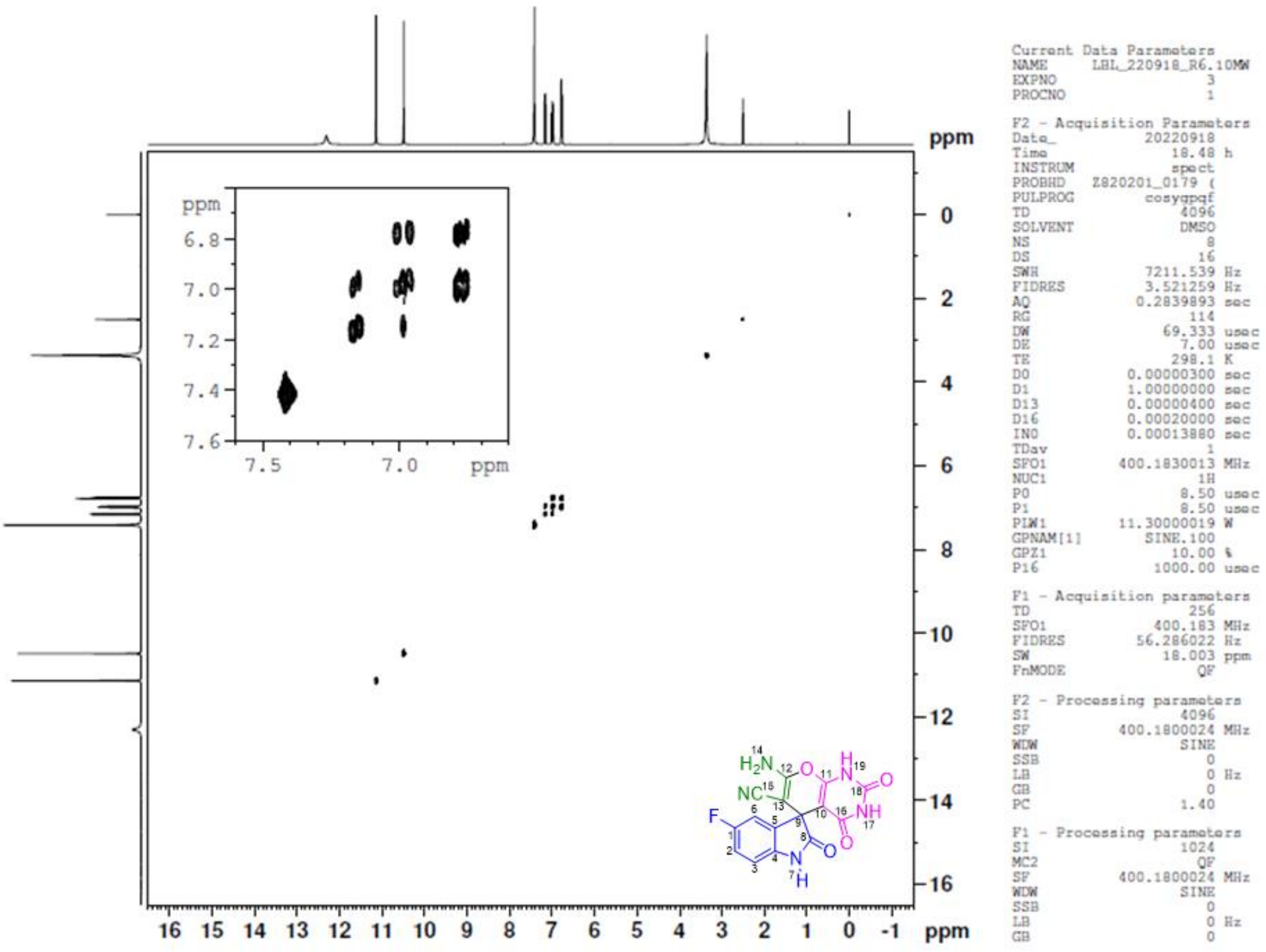

Figure S27. ^1H-^1H COSY NMR spectrum of compound **1d**.

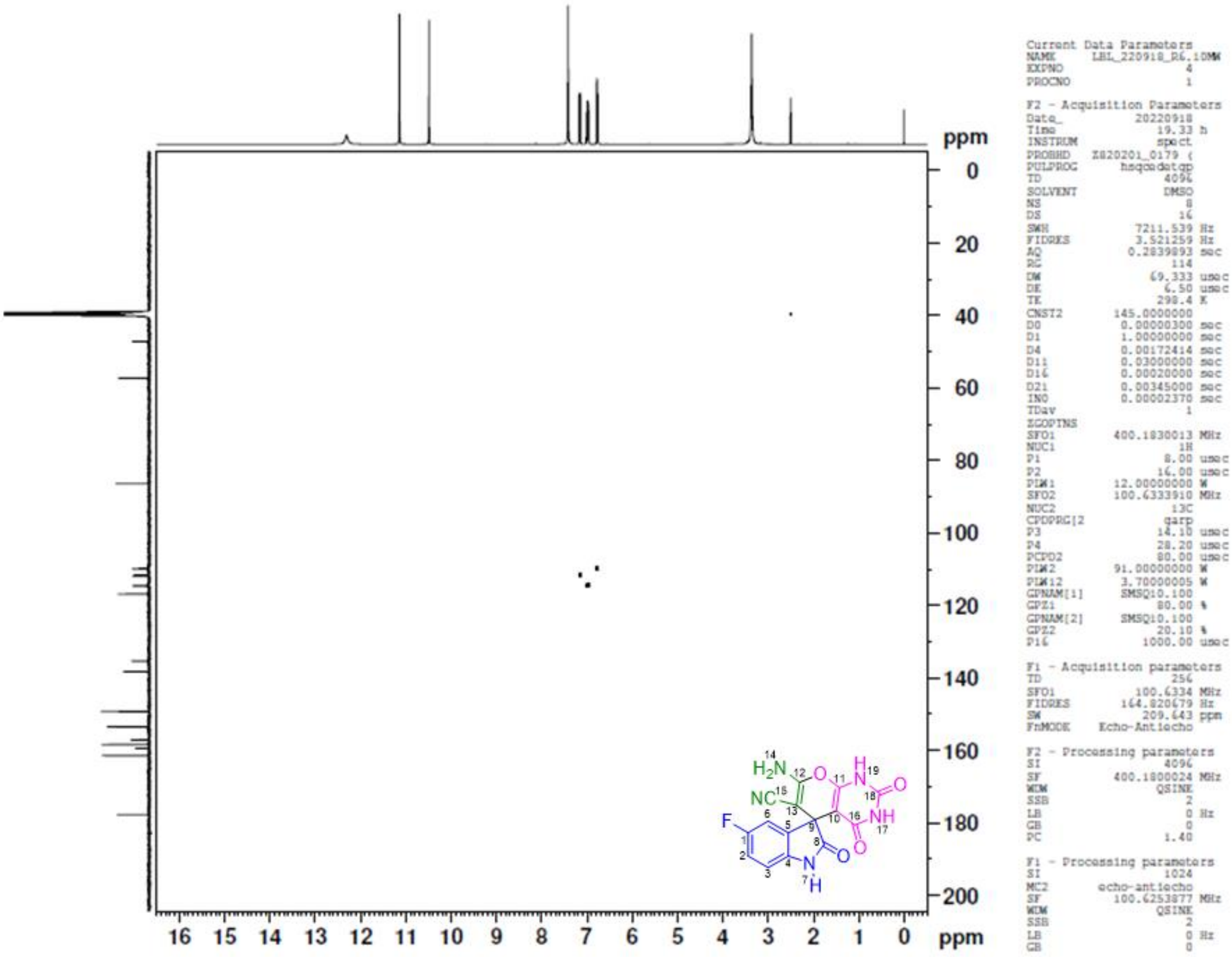

Figure S28. ^{1}H-^{13}C HSQC NMR spectrum of compound **1d**.

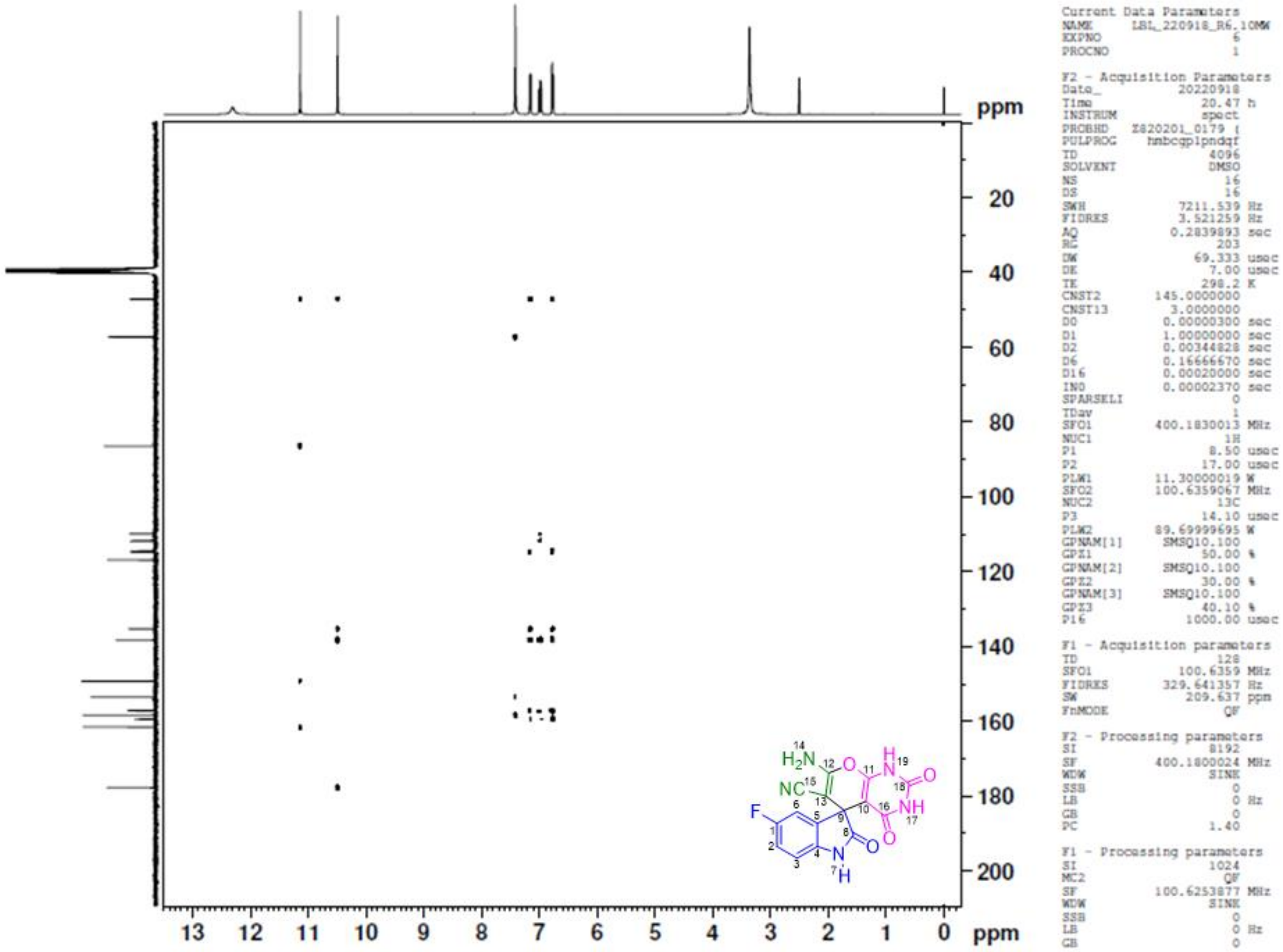

Figure S29. ^{1}H-^{13}C HMBC NMR spectrum of compound **1d** (cnst13 = 3 Hz).

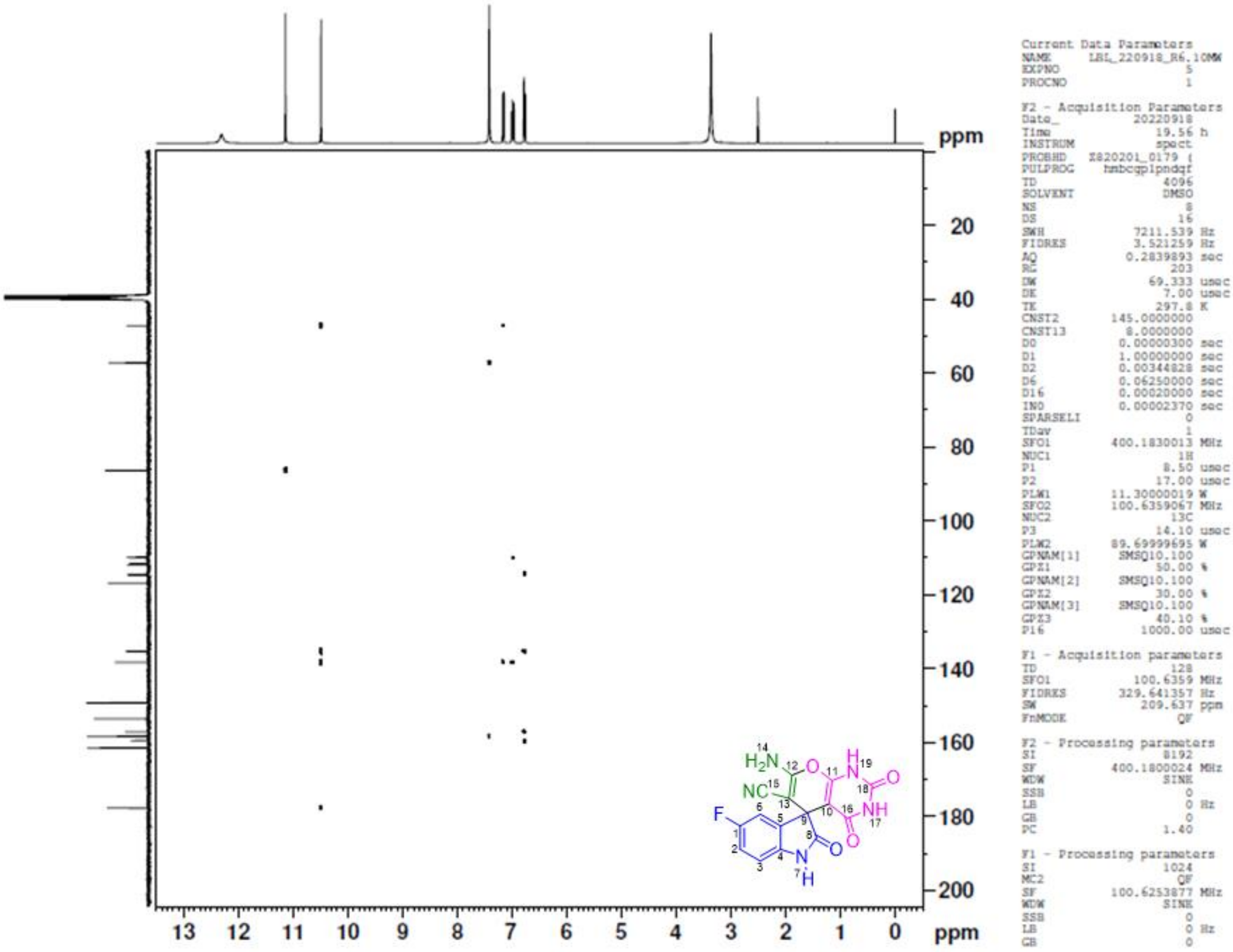

Figure S30. ^{1}H-^{13}C HMBC NMR spectrum of compound **1d** (cnst13 = 8 Hz).

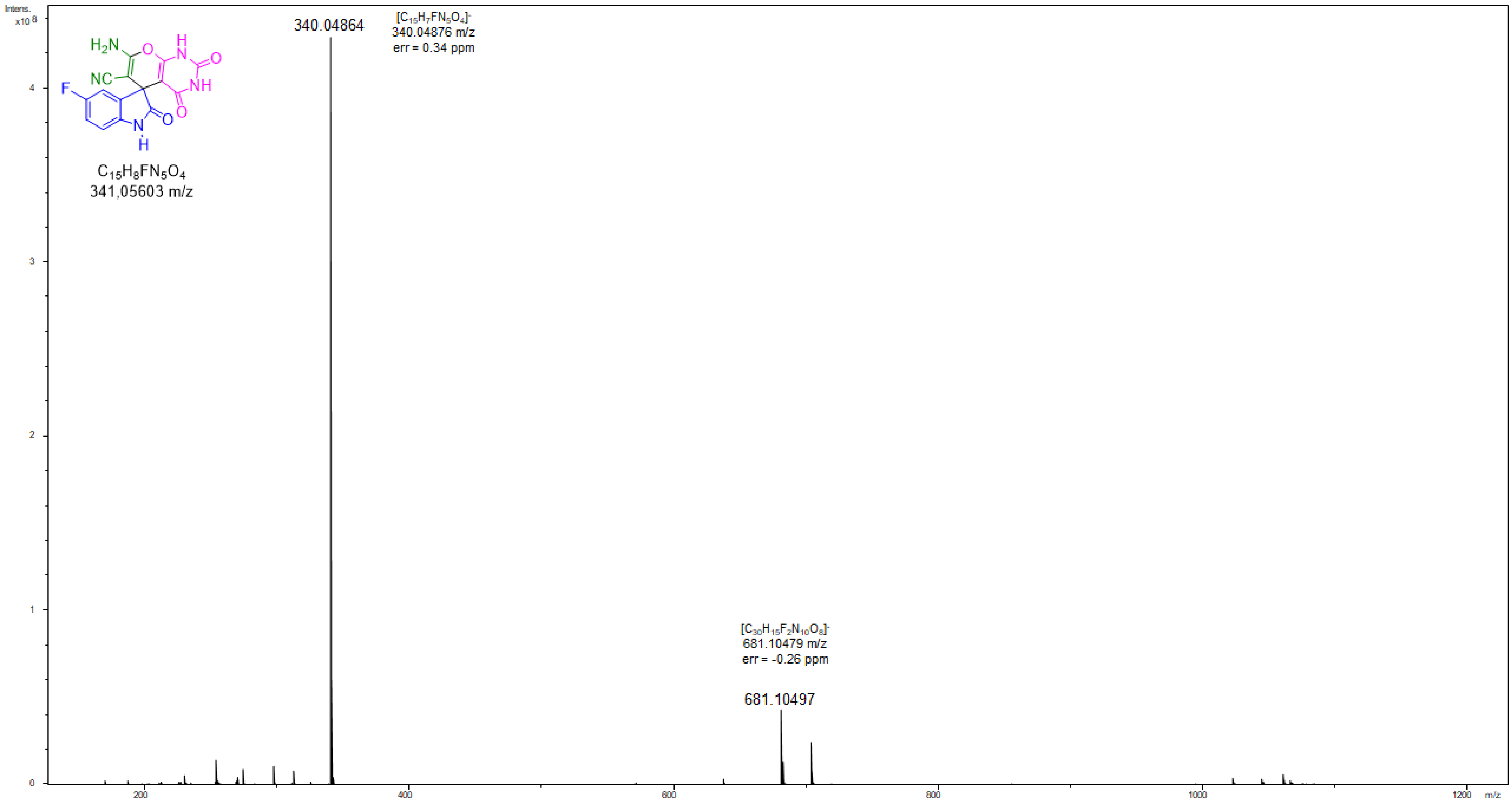

Figure S31. Mass spectrum of compound **1d**.

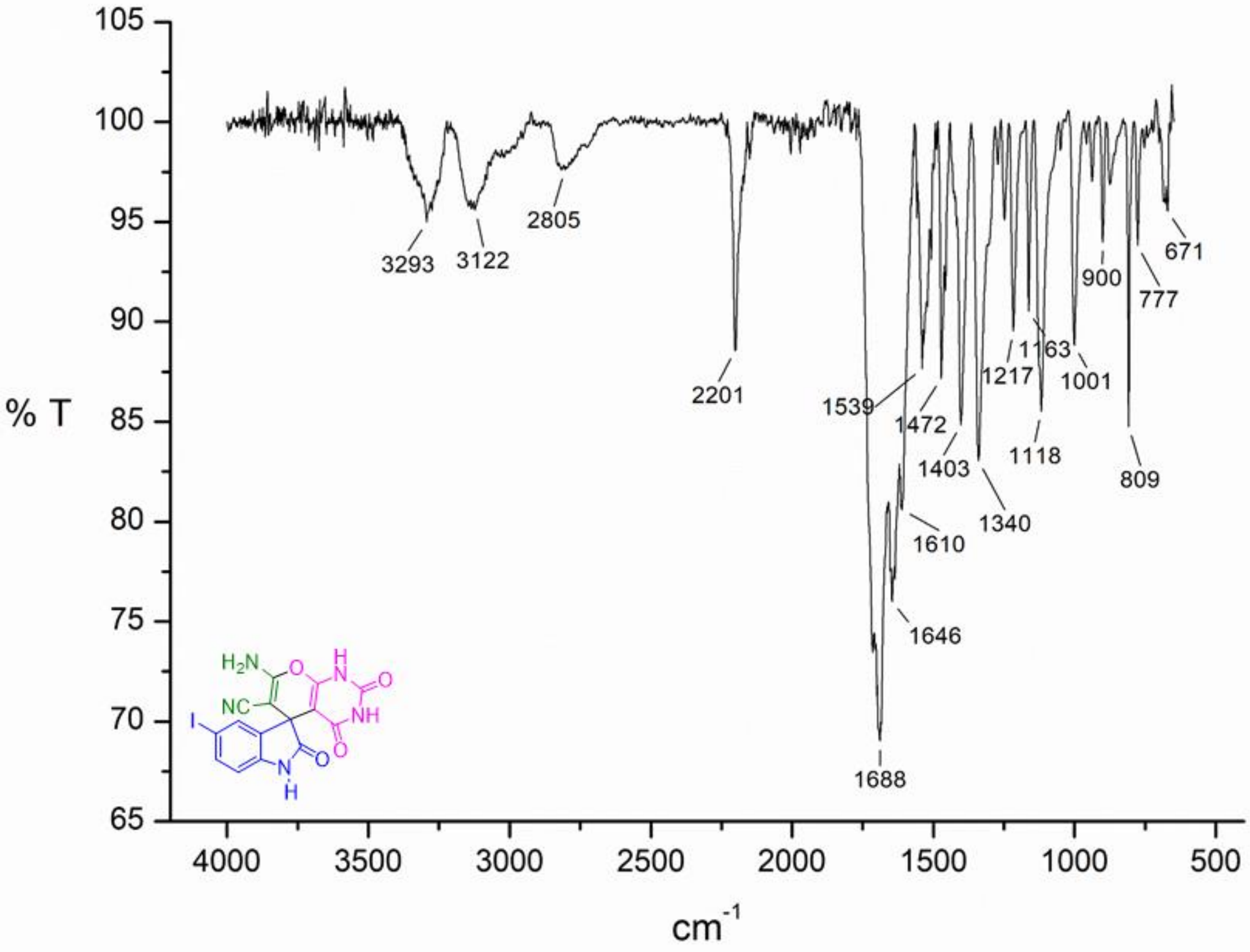

Figure S32. Infrared spectrum of compound **1e**.

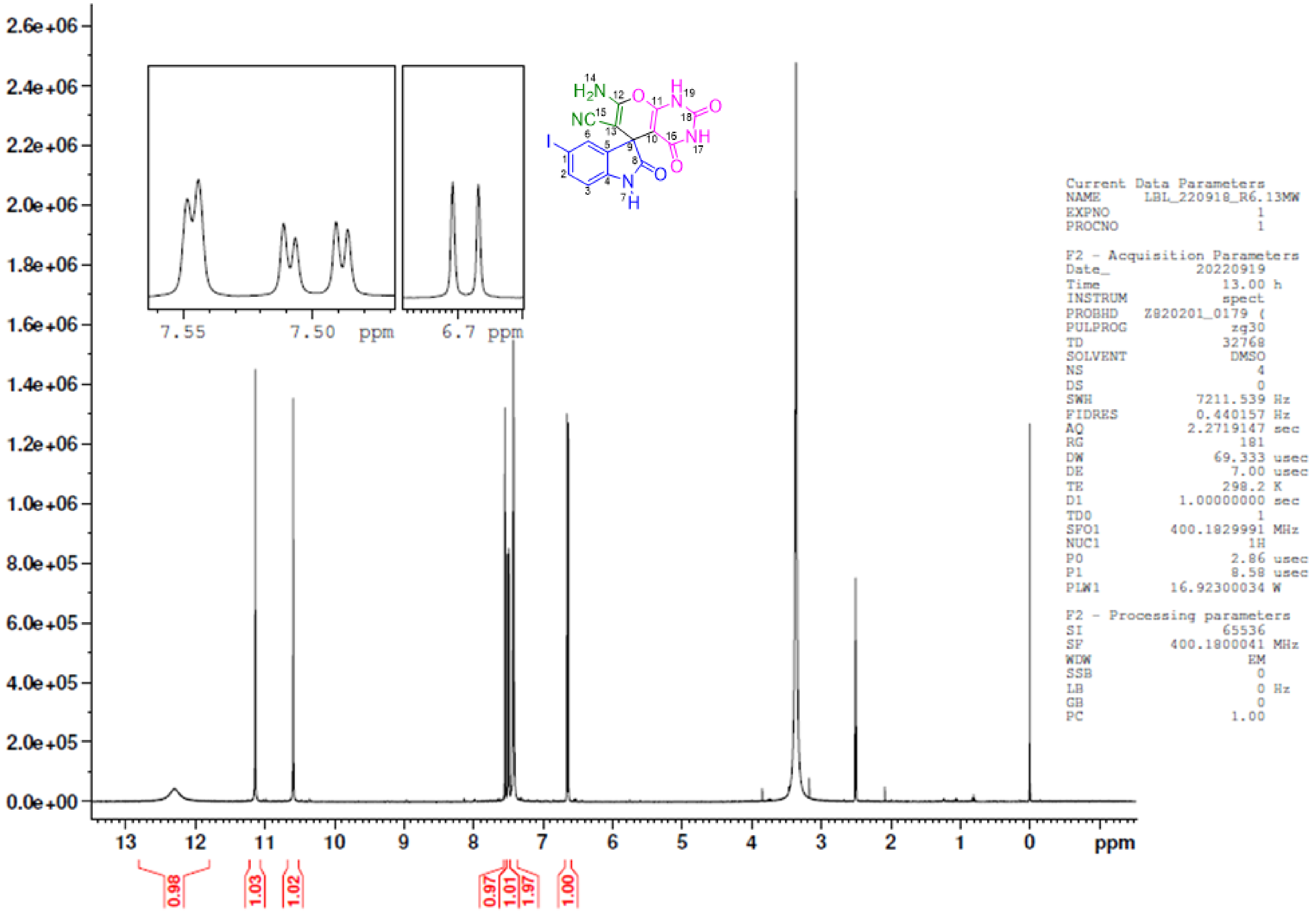

Figure S33. ^{1}H NMR spectrum of compound **1e**.

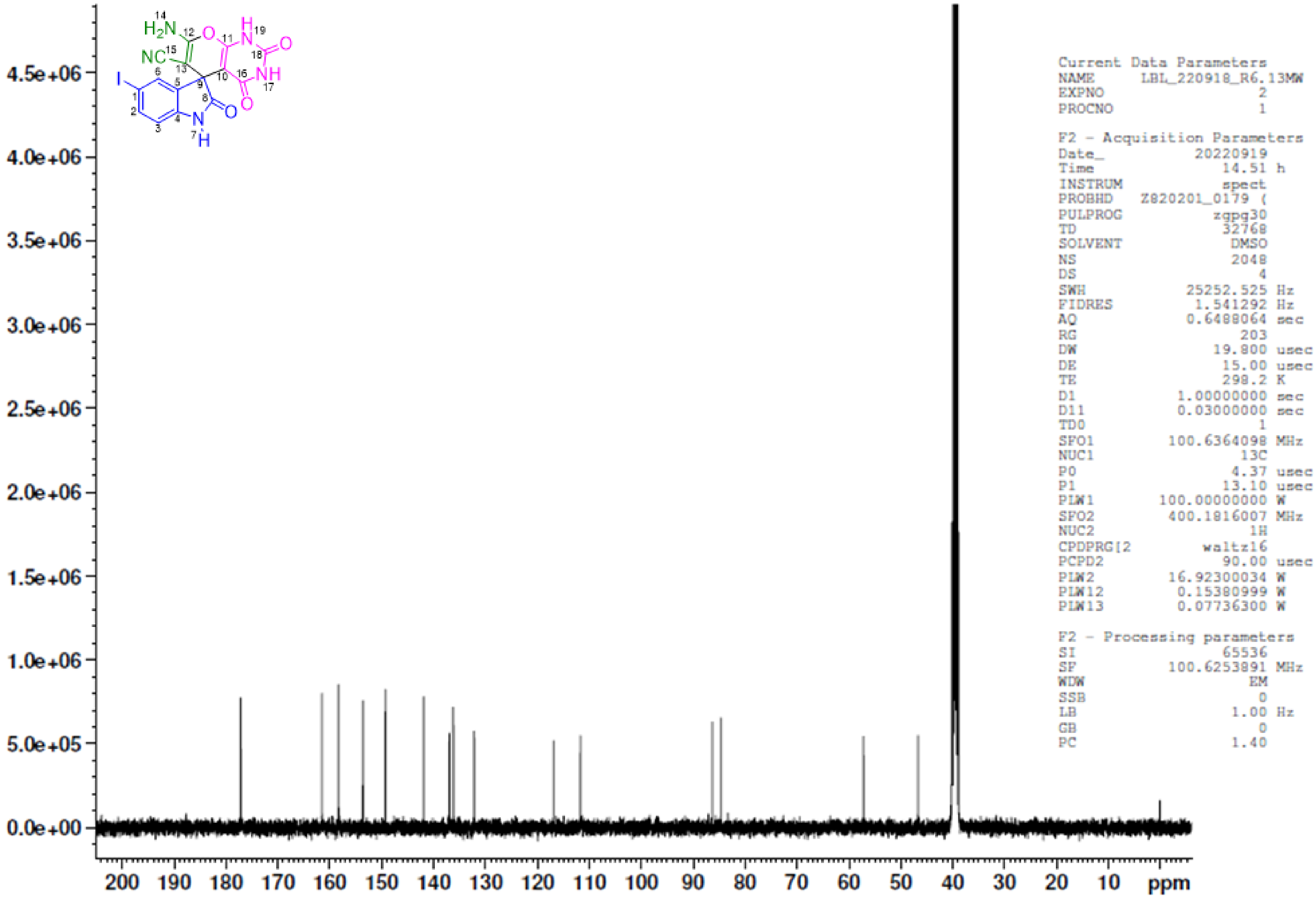

Figure S34. ^{13}C NMR spectrum of compound **1e**.

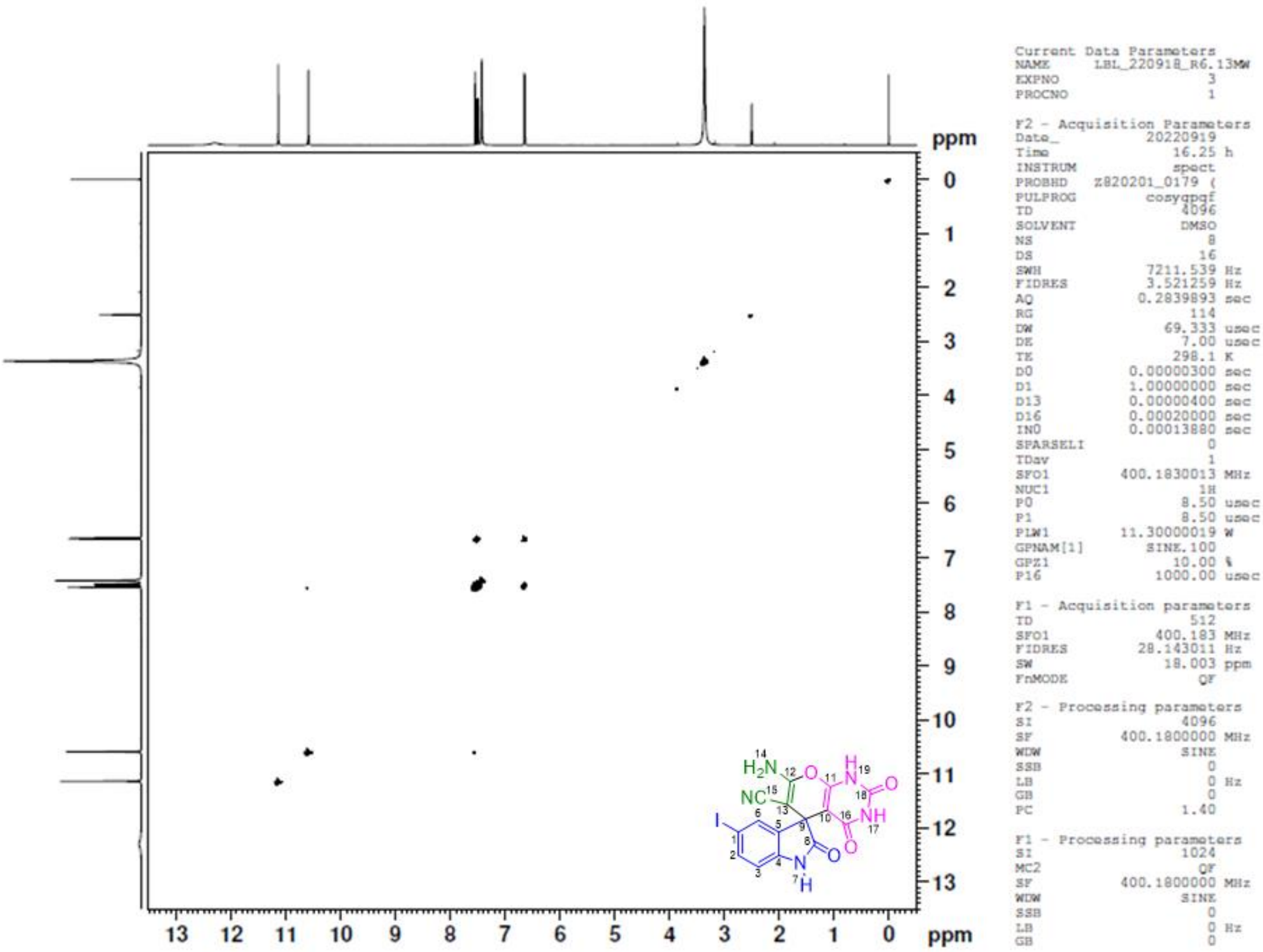

Figure S35. ^1H-^1H COSY NMR spectrum of compound **1e**.

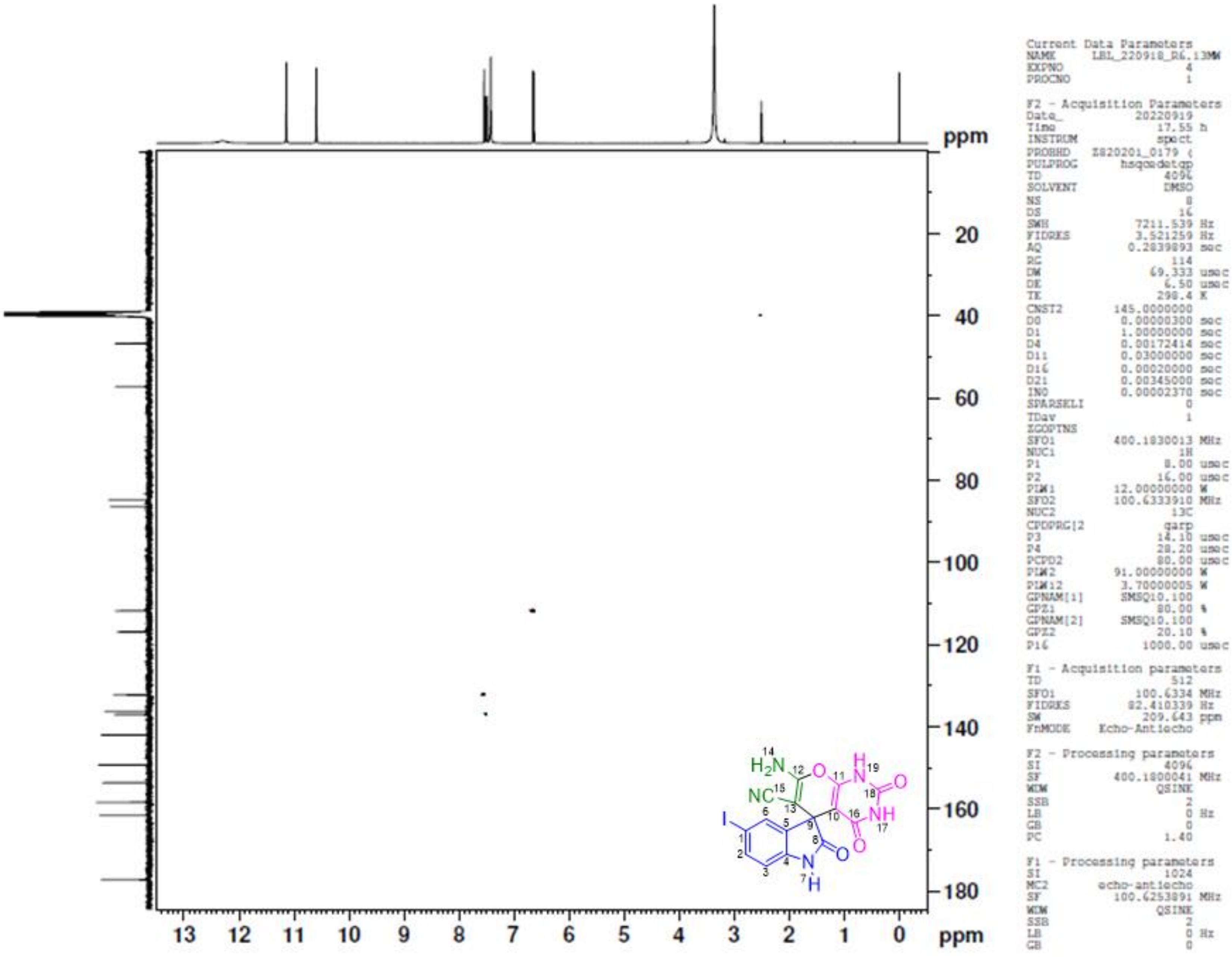

Figure S36. ^1H-^{13}C HSQC NMR spectrum of compound **1e**.

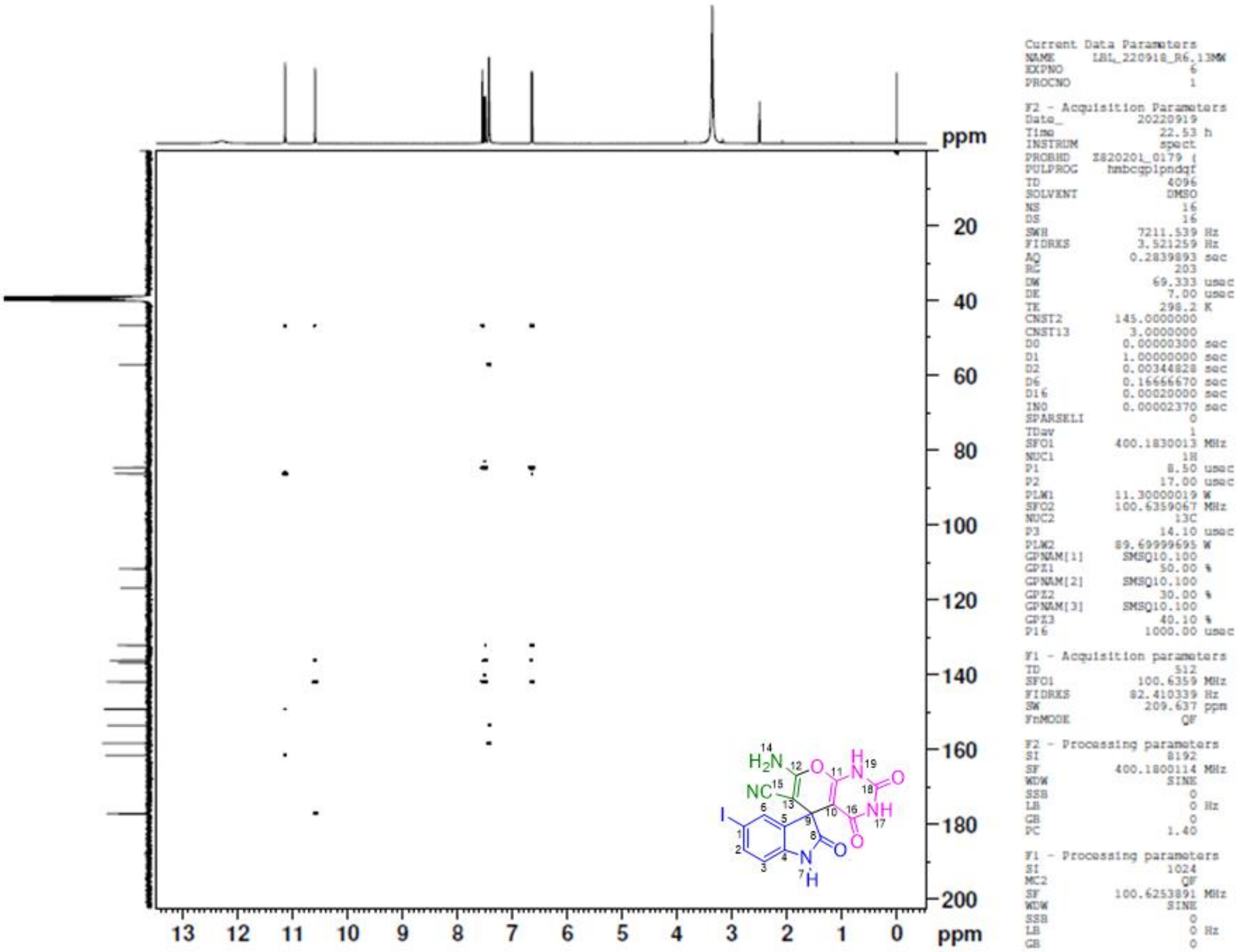

Figure S37. ^{1}H-^{13}C HMBC NMR spectrum of compound **1e** (cnst13 = 3 Hz).

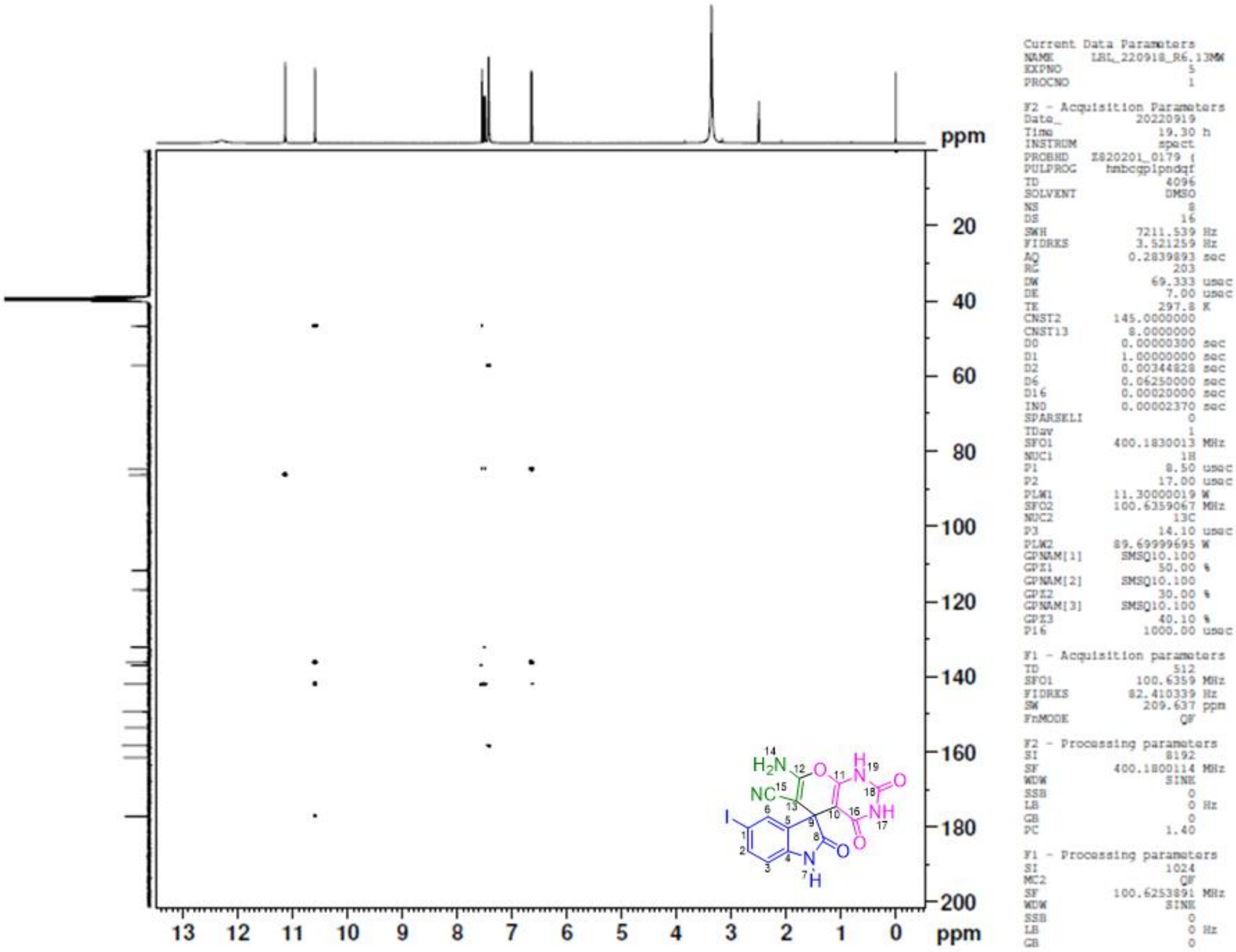

Figure S38. ^{1}H-^{13}C HMBC NMR spectrum of compound **1e** (cnst13 = 8 Hz).

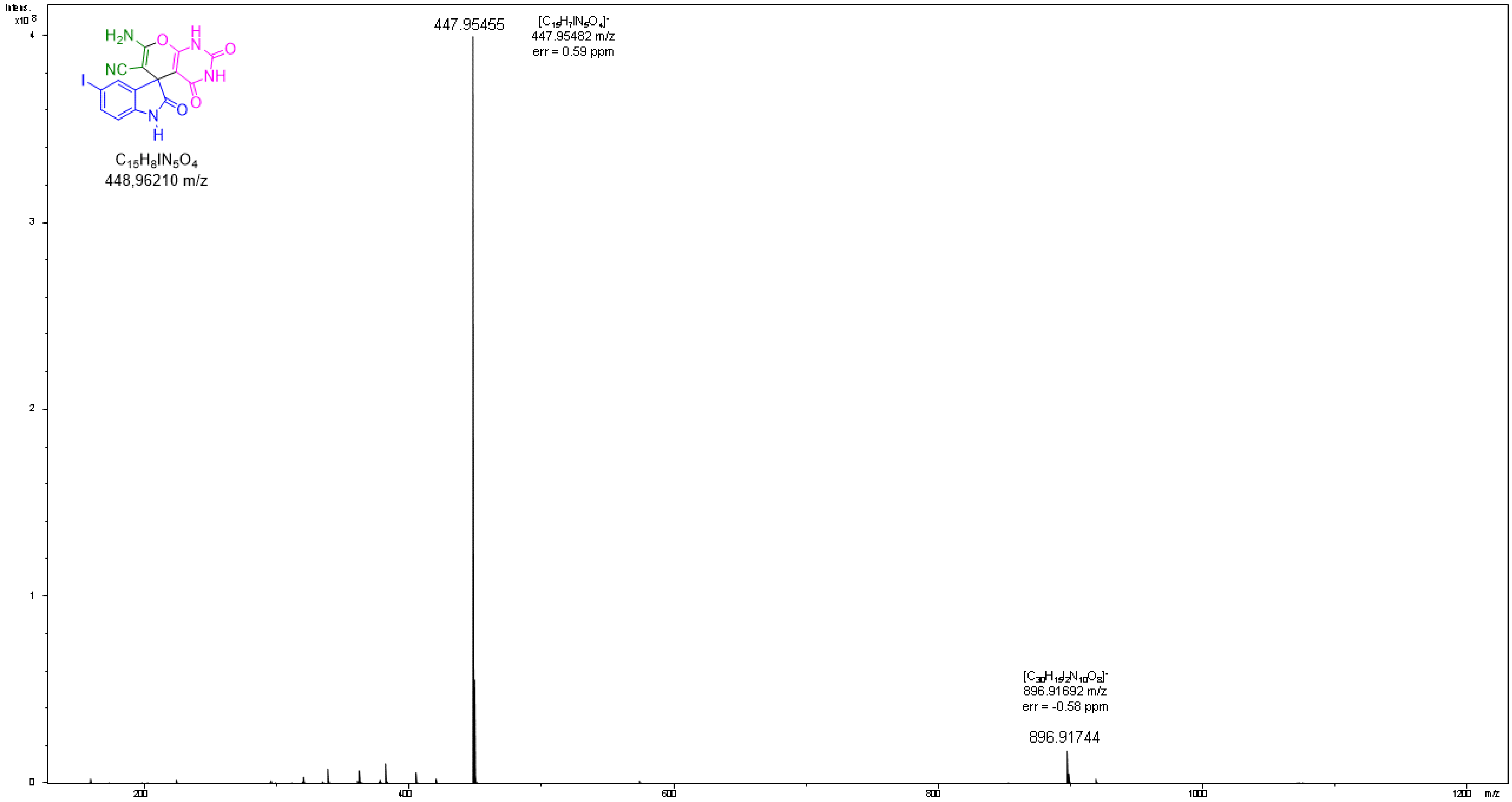

Figure S39. Mass spectrum of compound **1e**.

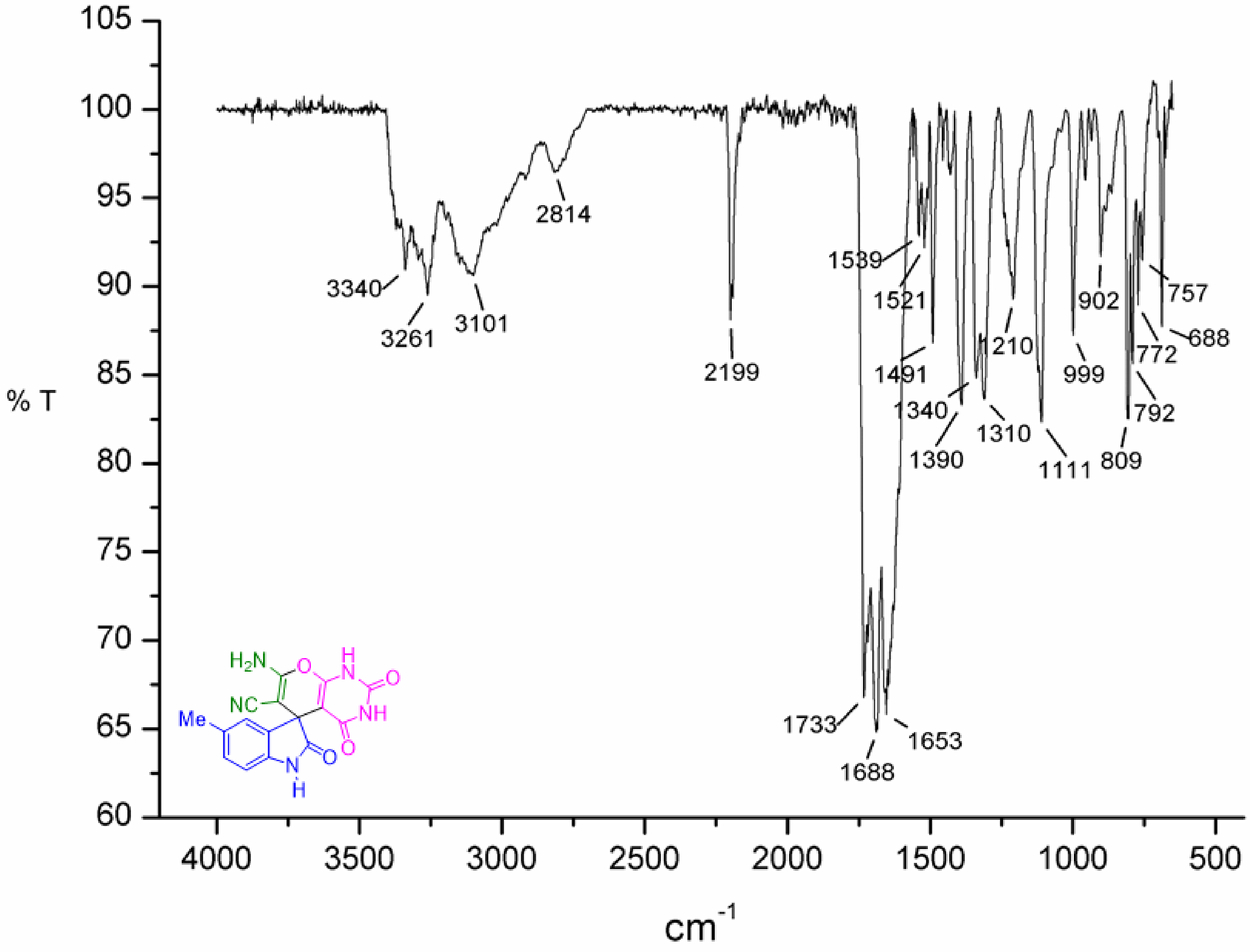

Figure S40. Infrared spectrum of compound **1f**.

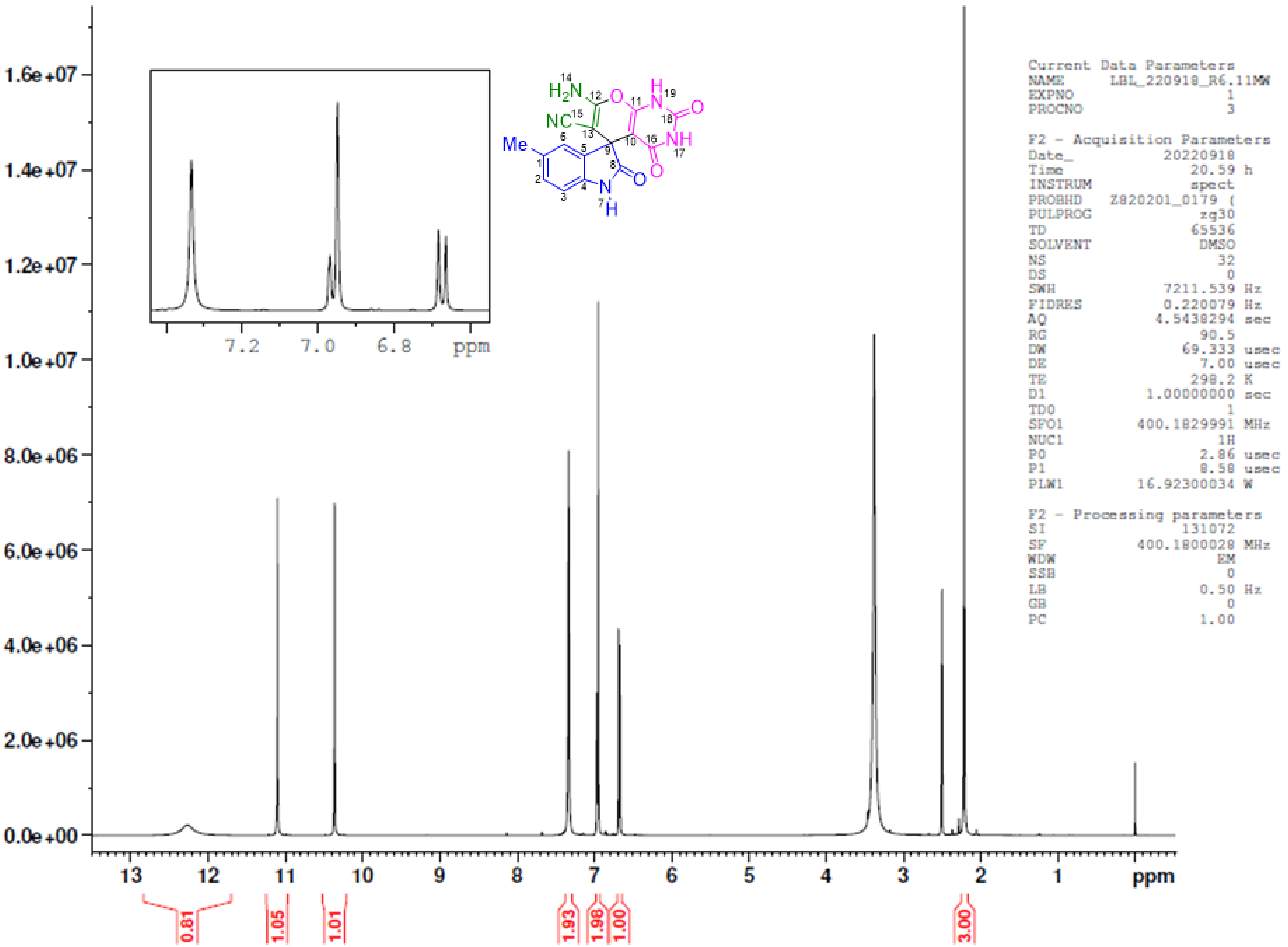

Figure S41. ^{1}H NMR spectrum of compound **1f**.

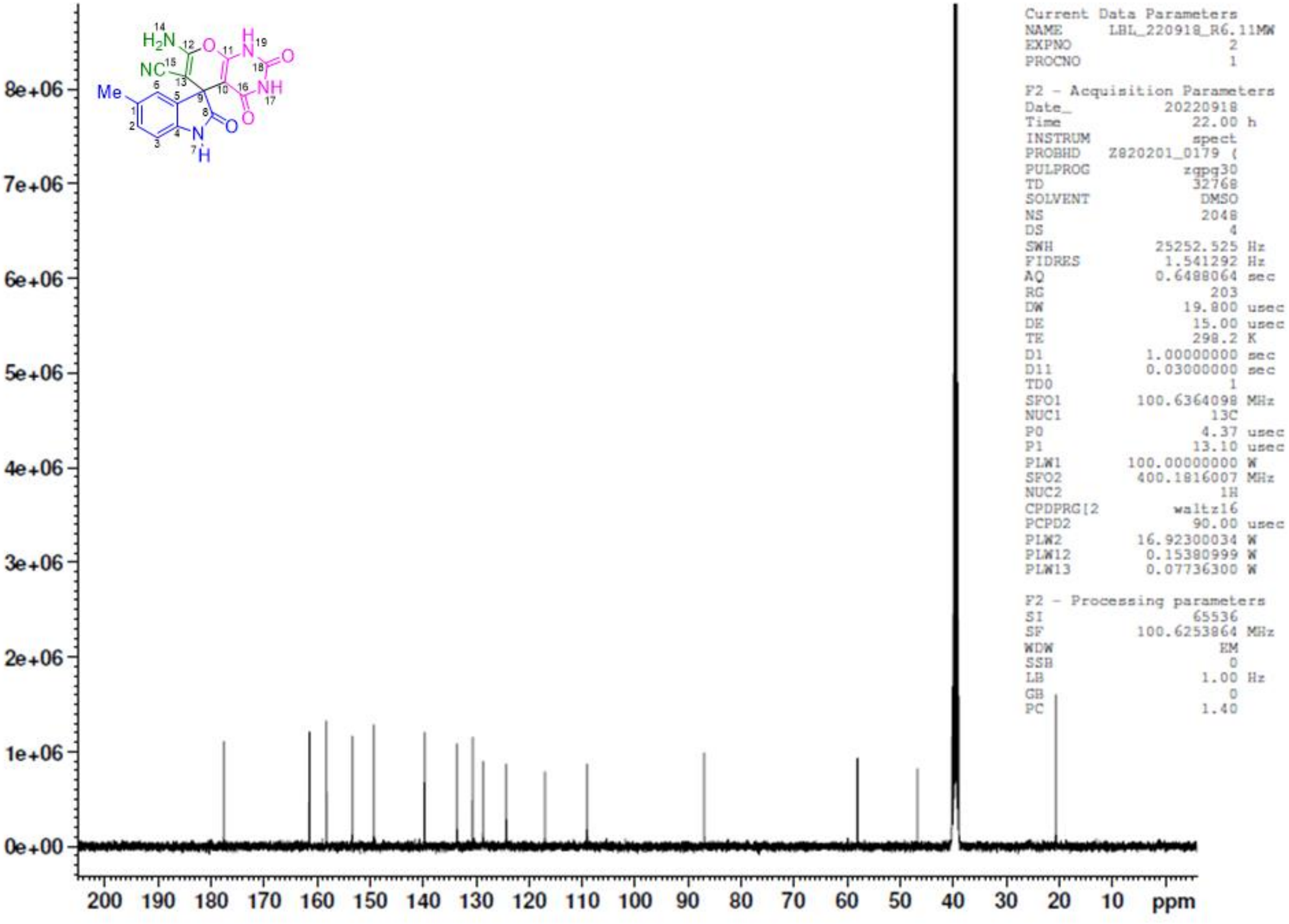

Figure S42. ^{13}C NMR spectrum of compound **1f**.

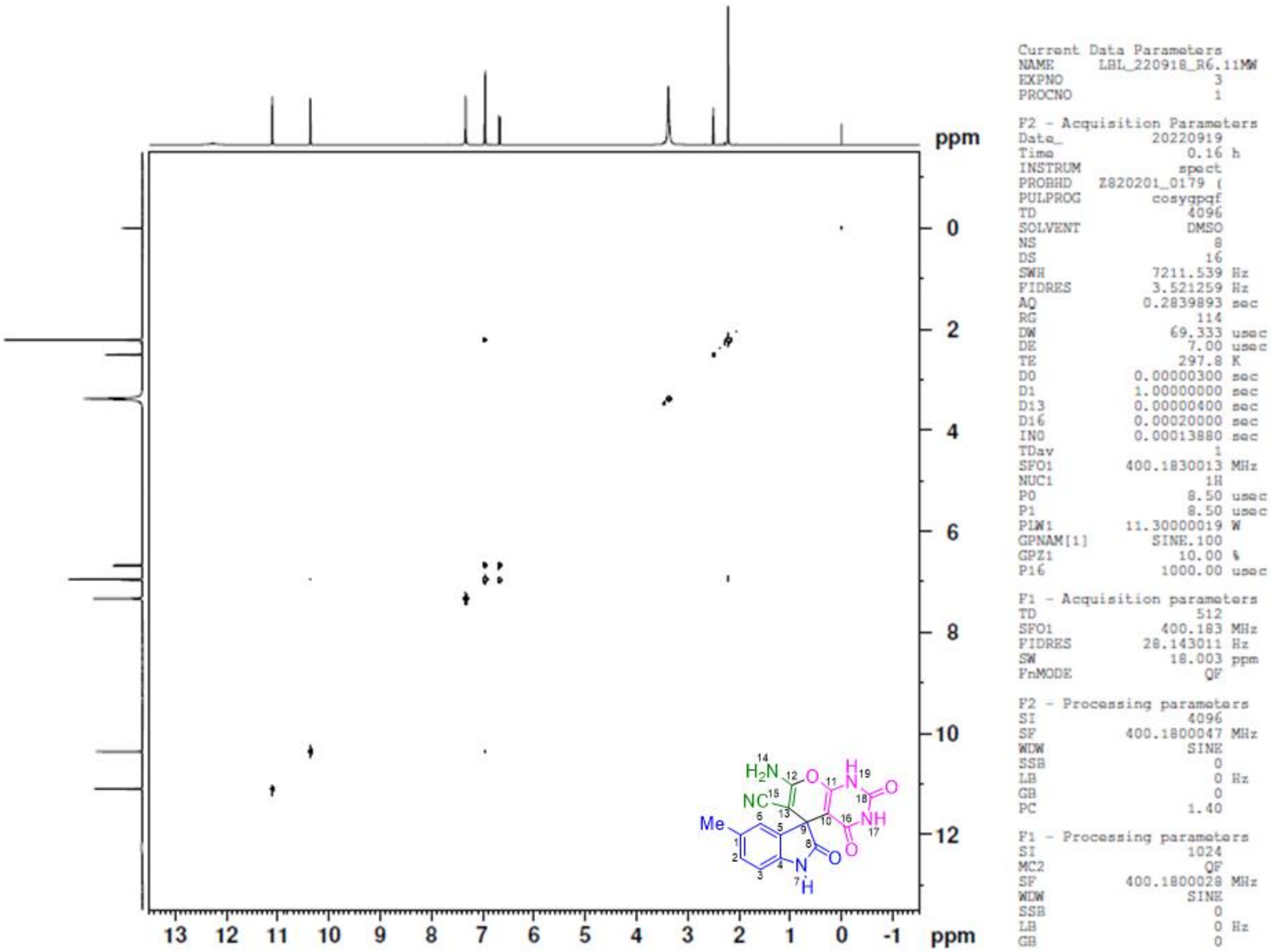

Figure S43. ^1H-^1H COSY NMR spectrum of compound **1f**.

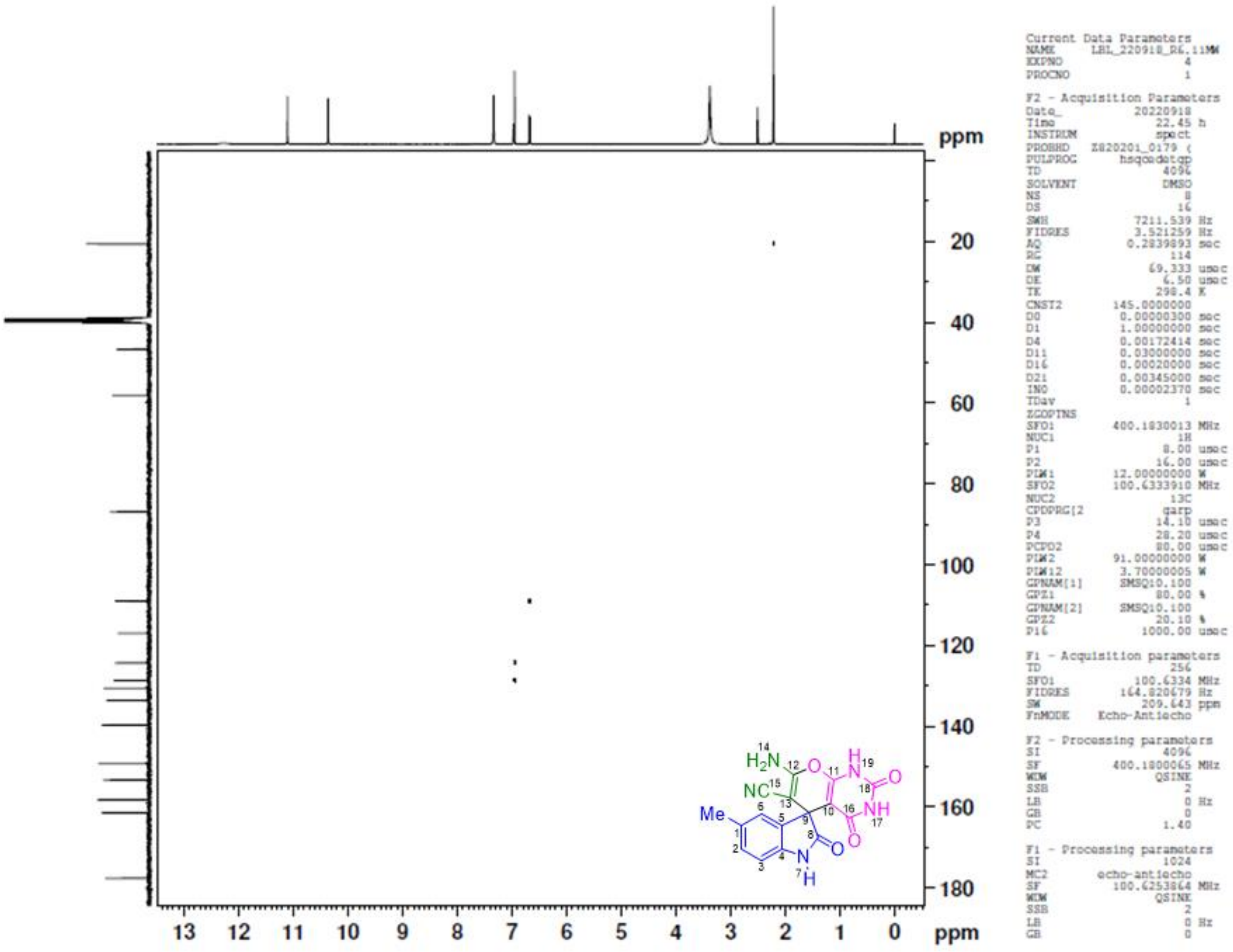

Figure S44. ^1H-^{13}C HSQC NMR spectrum of compound **1f**.

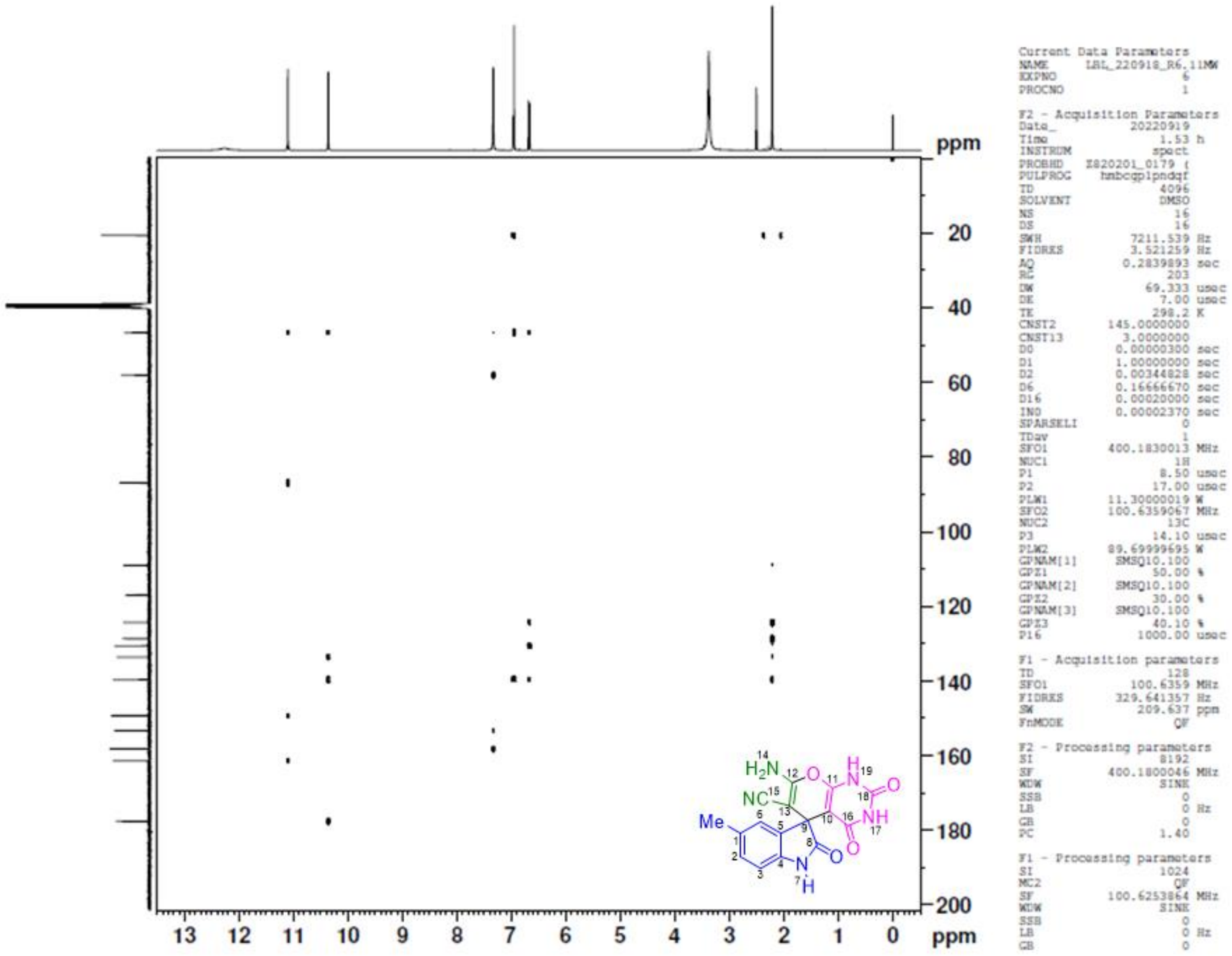

Figure S45. ^{1}H-^{13}C HMBC NMR spectrum of compound **1f** (cnst13 = 3 Hz).

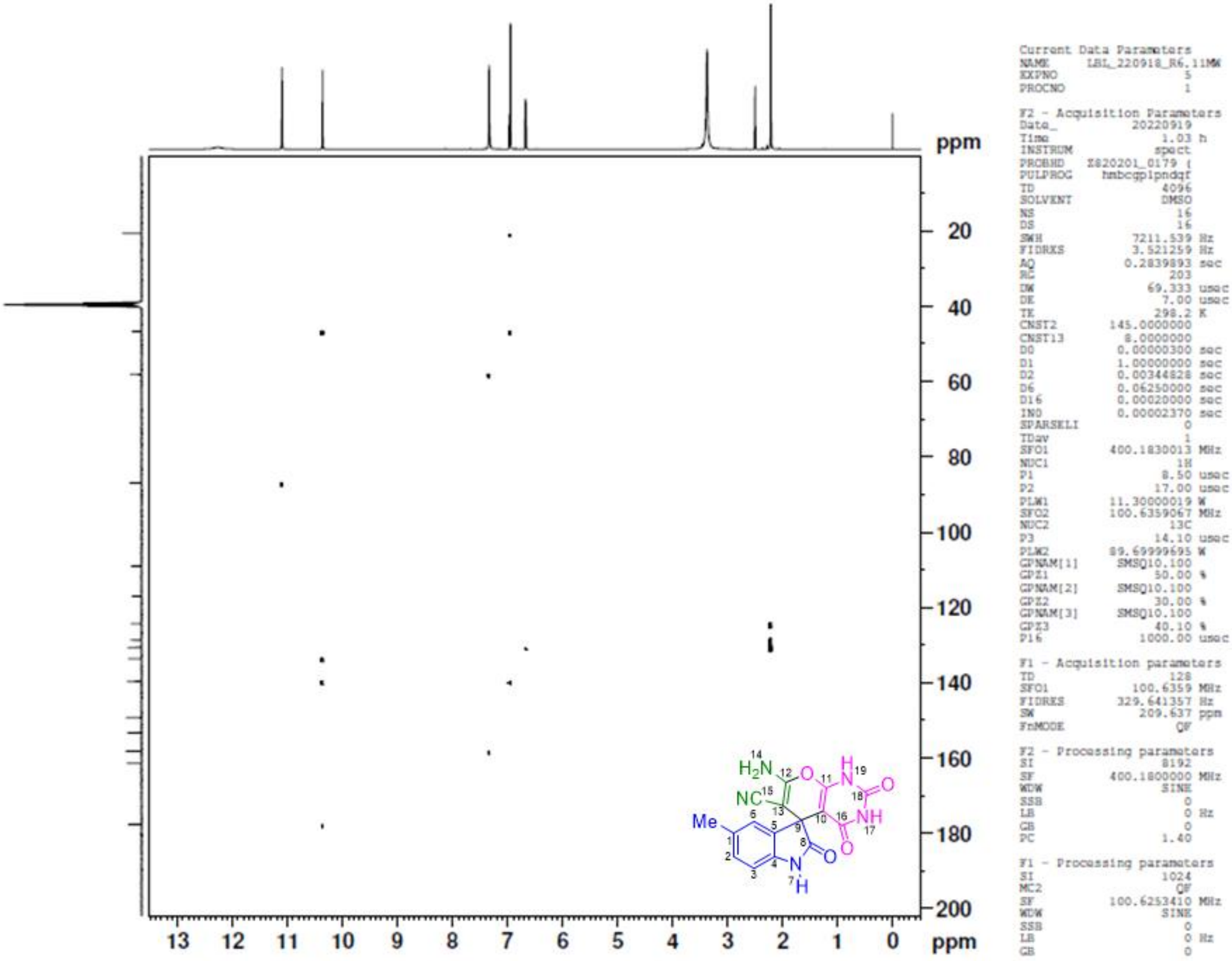

Figure S46. ^{1}H-^{13}C HMBC NMR spectrum of compound **1f** (cnst13 = 8 Hz).

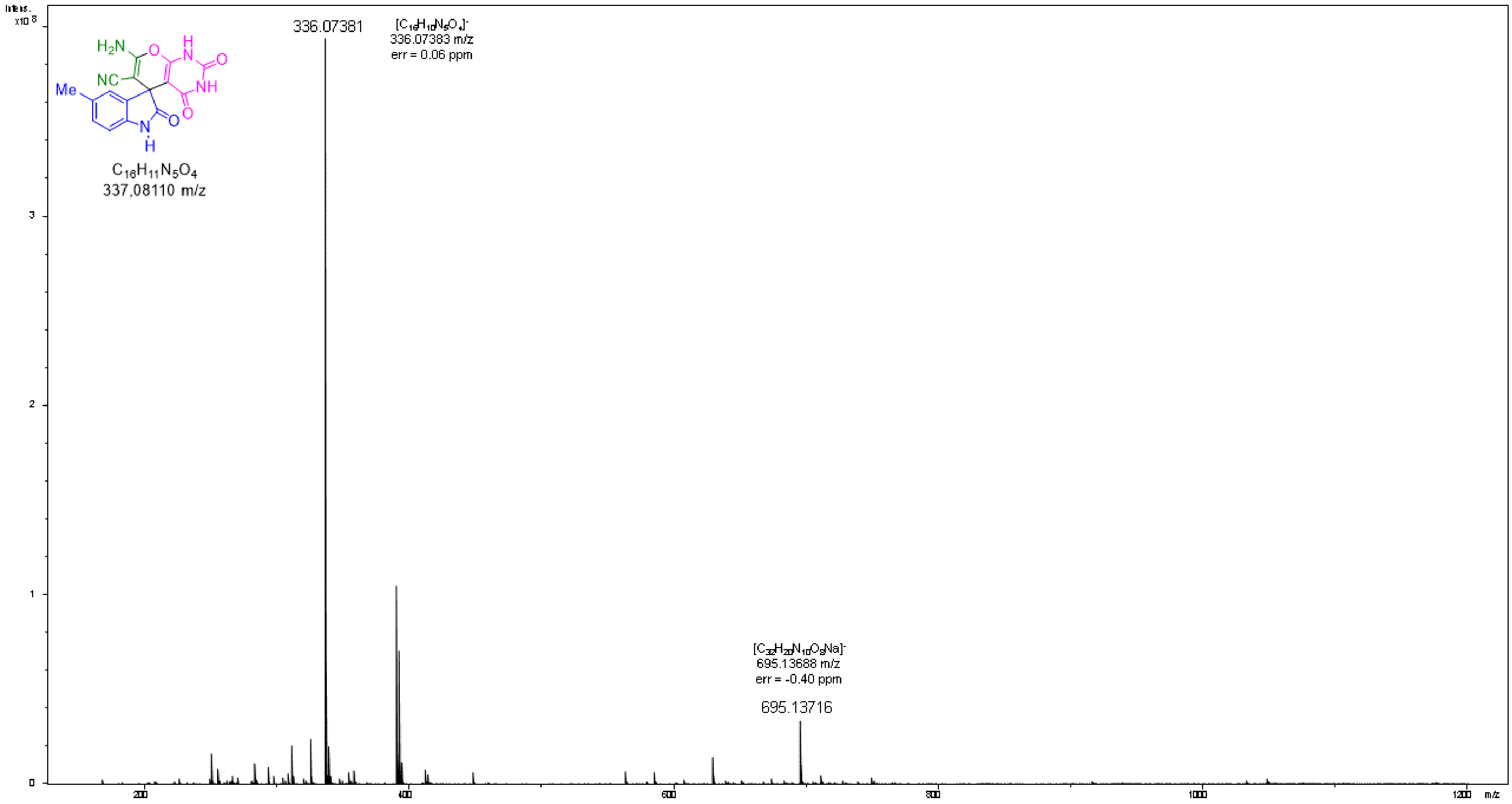

Figure S47. Mass spectrum of compound **1f**.

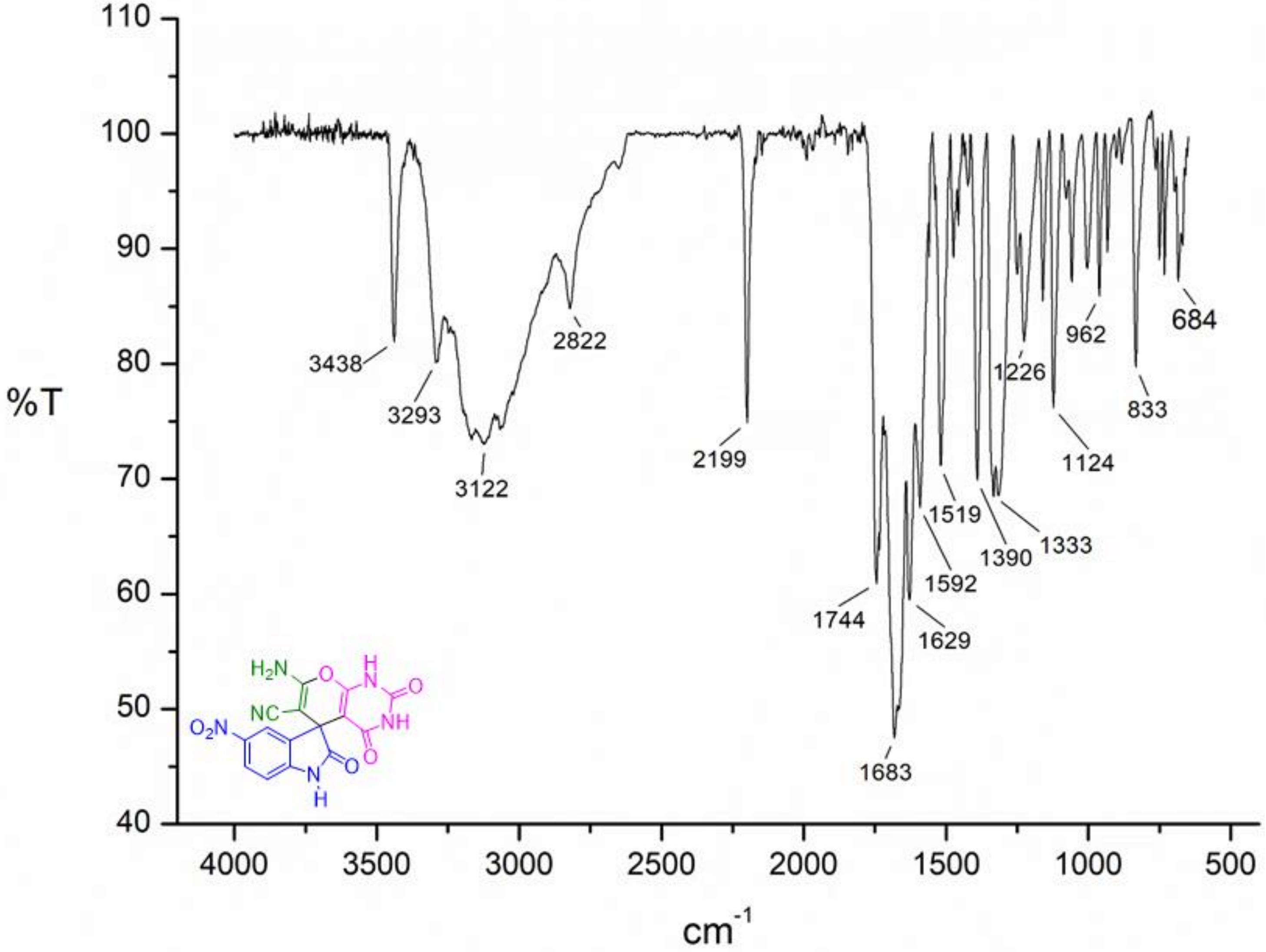

Figure S48. Infrared spectrum of compound **1g**.

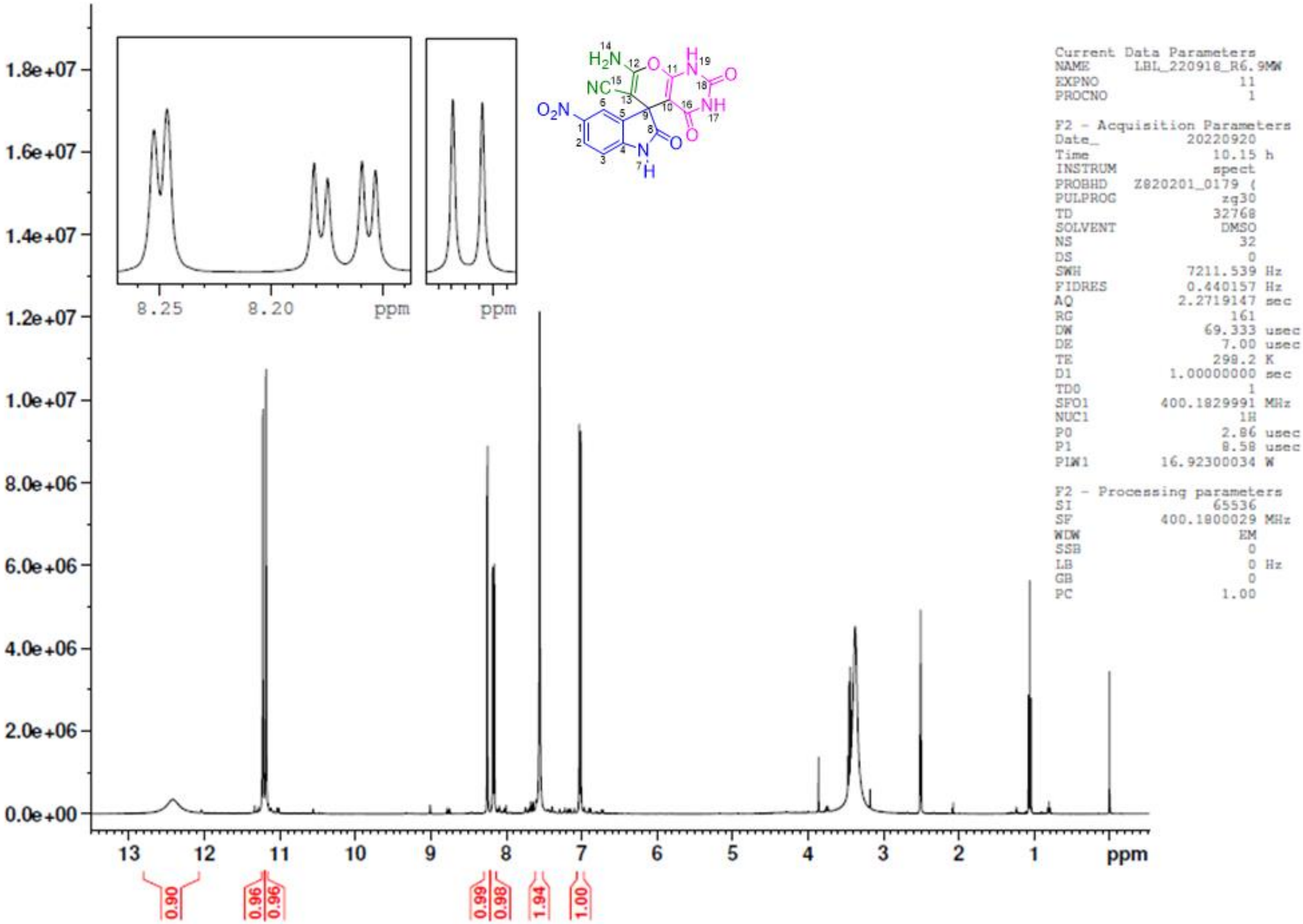

Figure S49. ^{1}H NMR spectrum of compound **1g**.

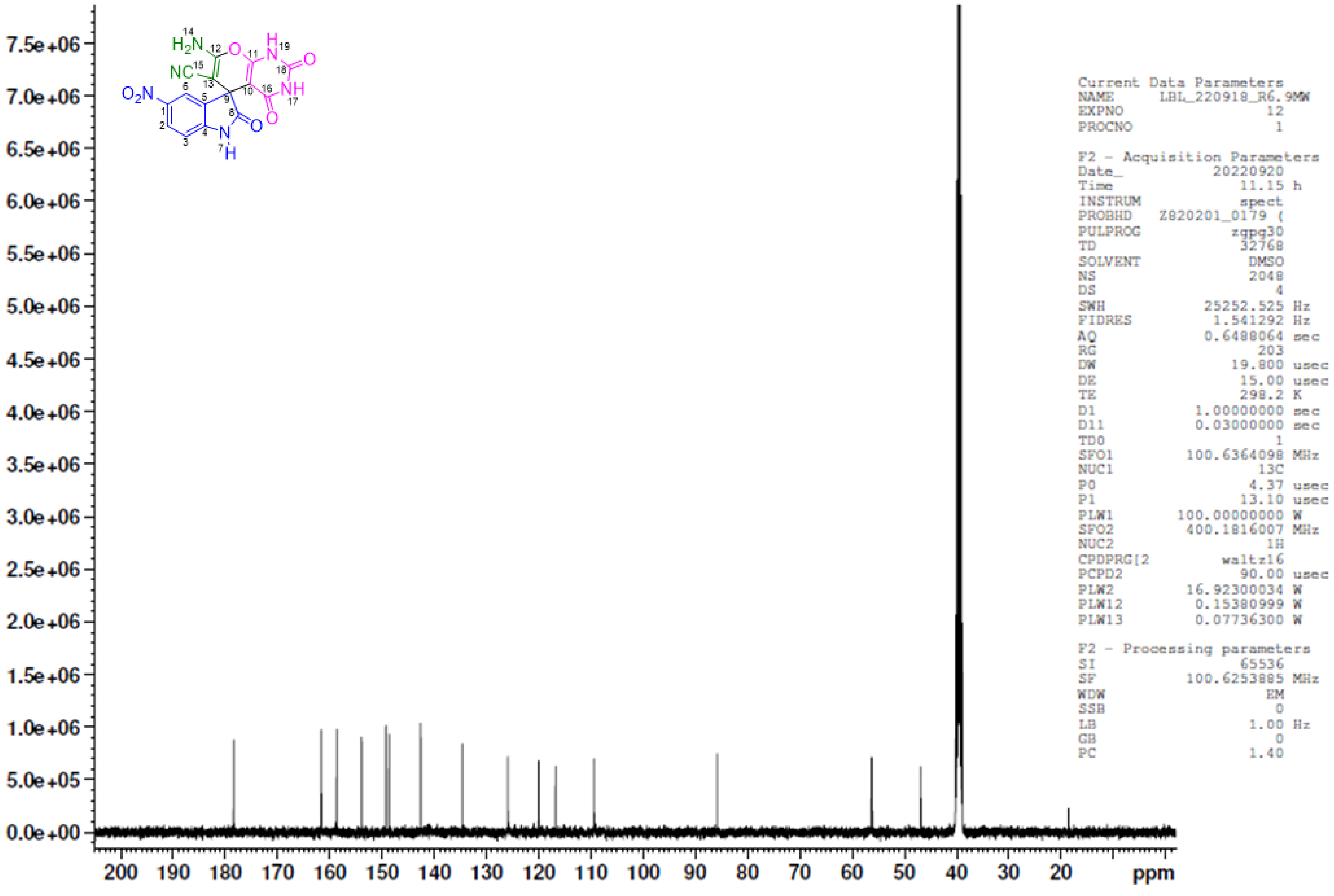

Figure S50. ^{13}C NMR spectrum of compound **1g**.

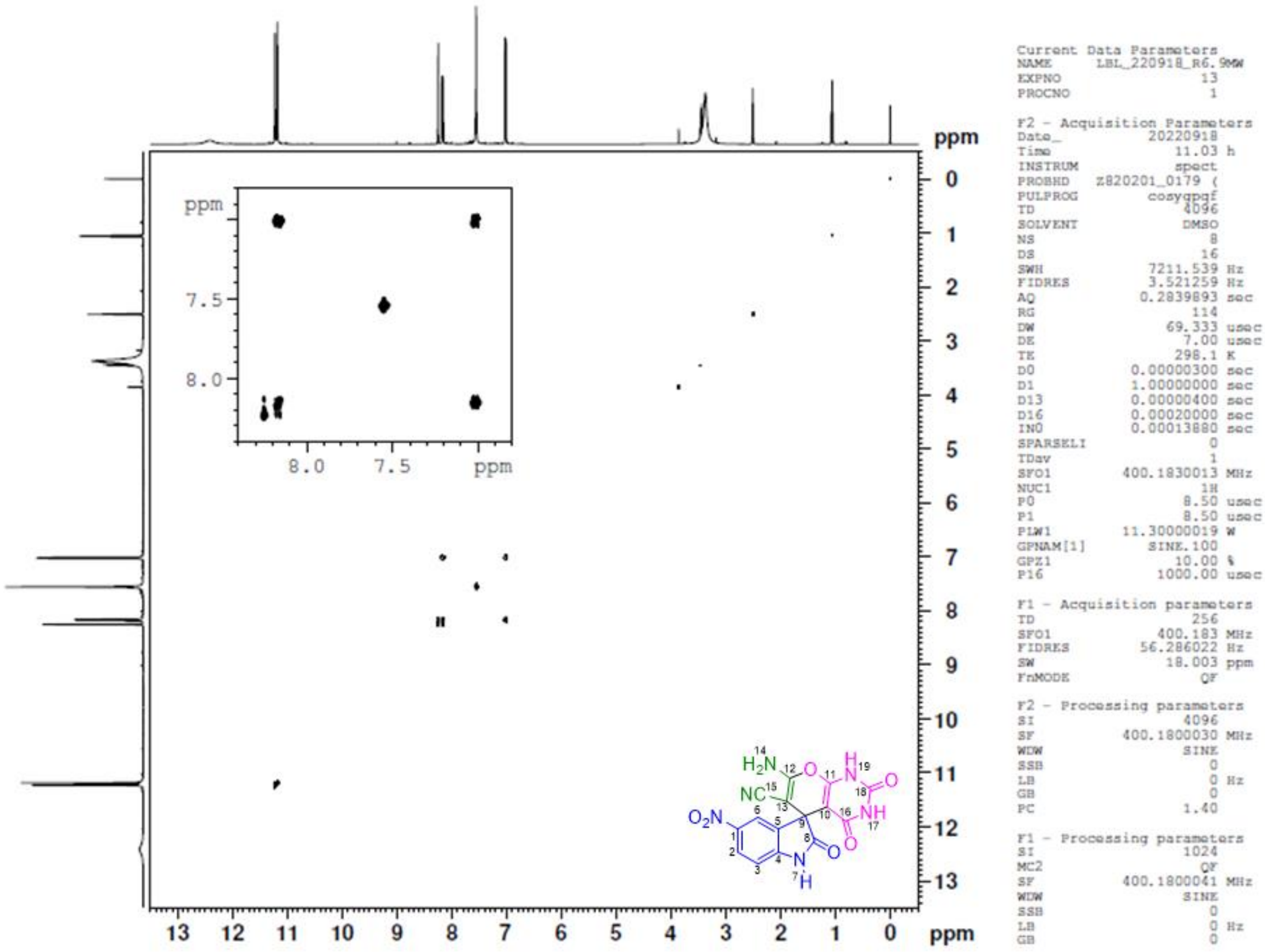

Figure S51. ^1H-^1H COSY NMR spectrum of compound **1g**.

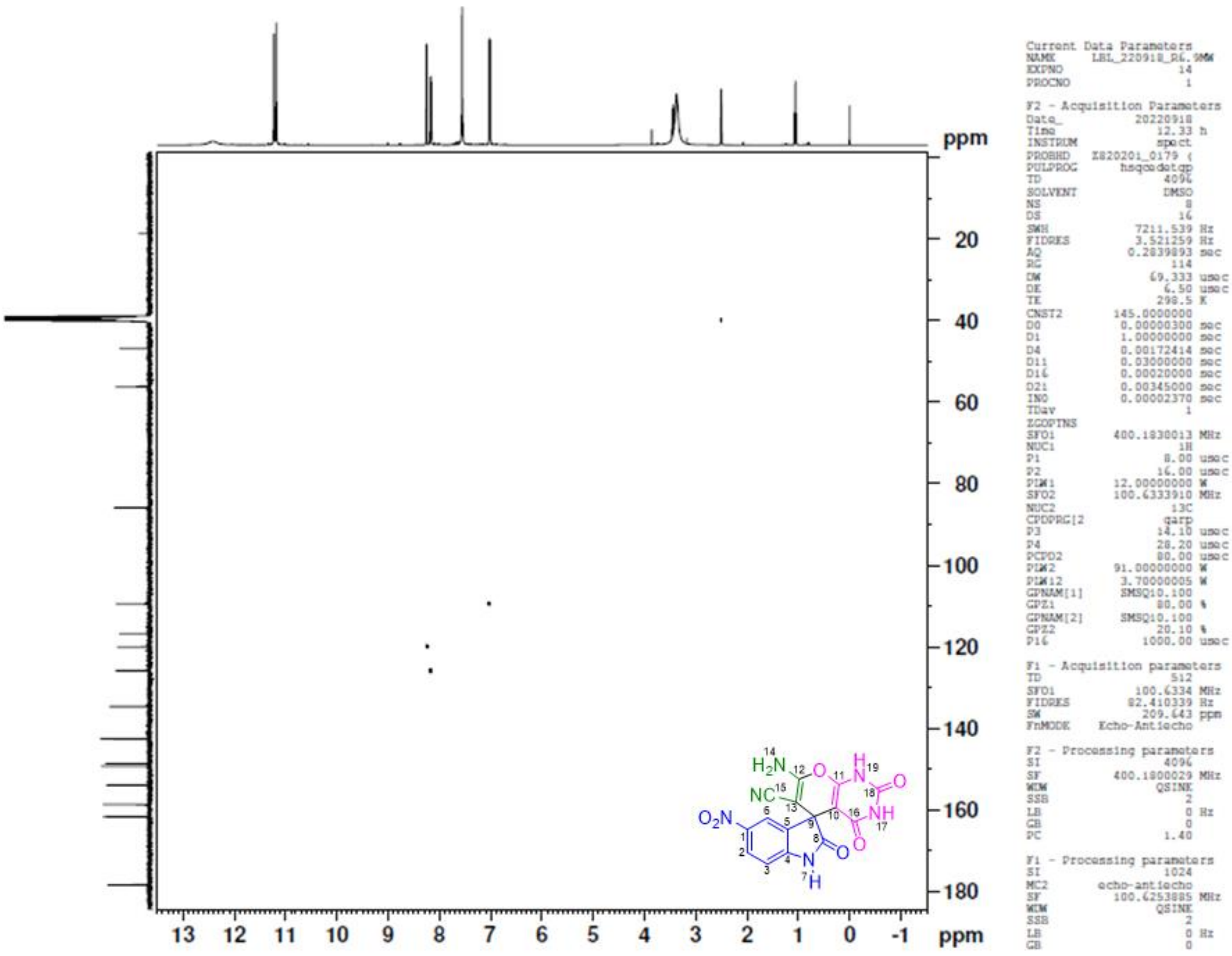

Figure S52. ^{1}H-^{13}C HSQC NMR spectrum of compound **1g**.

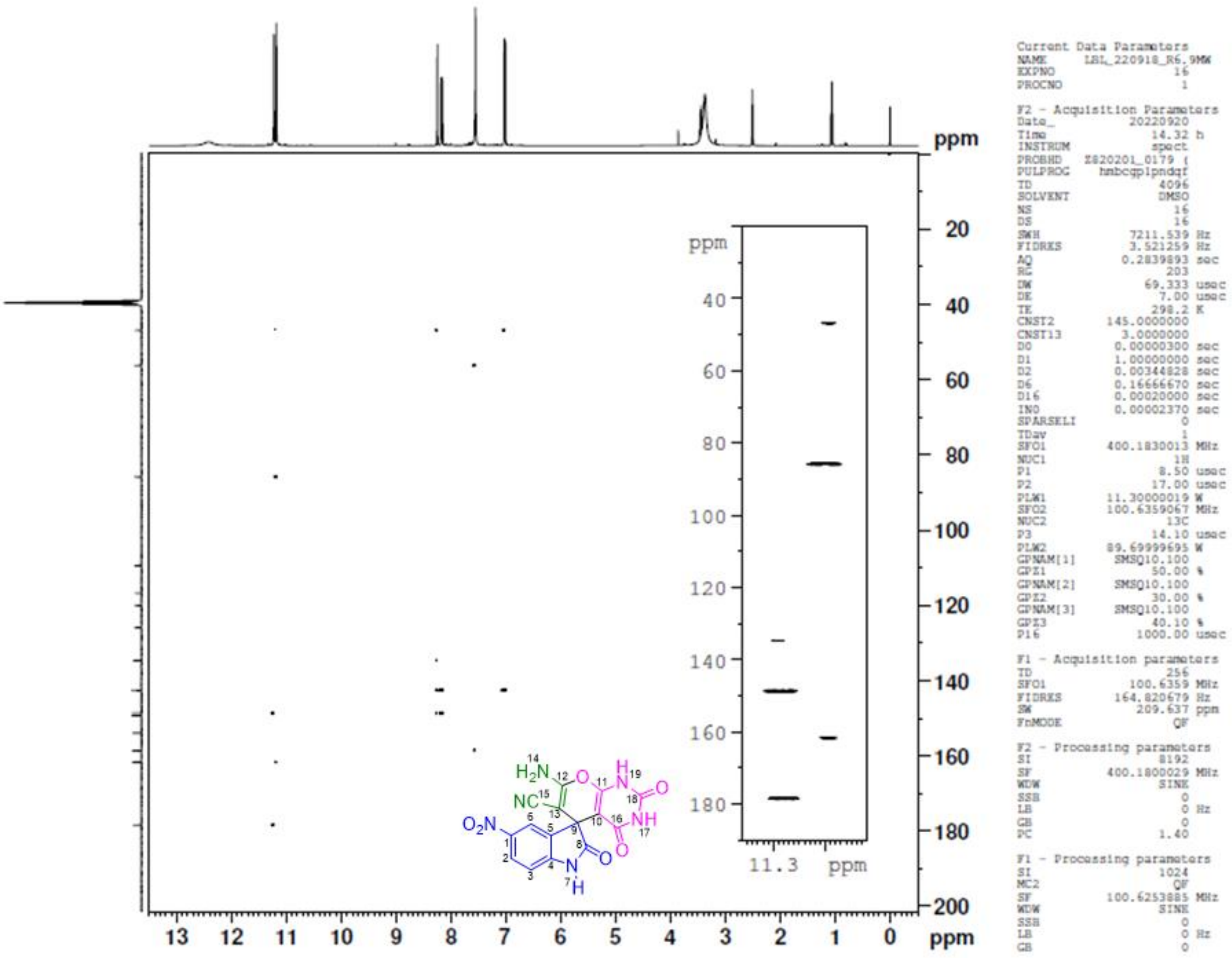

Figure S53. ^{1}H-^{13}C HMBC NMR spectrum of compound **1g** (cnst13 = 3 Hz).

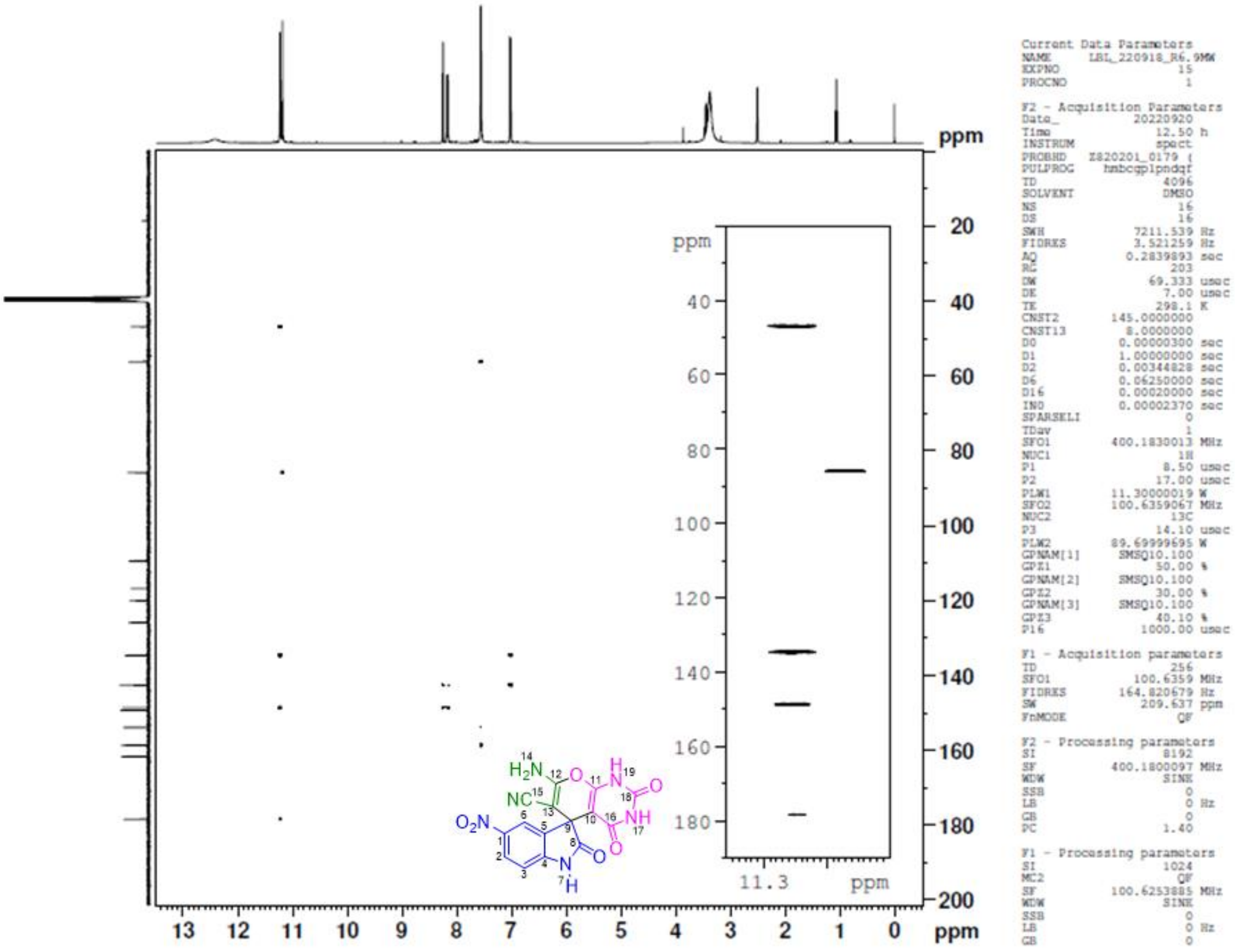

Figure S54. ^1H-^{13}C HMBC NMR spectrum of compound **1g** (cnst13 = 8 Hz).

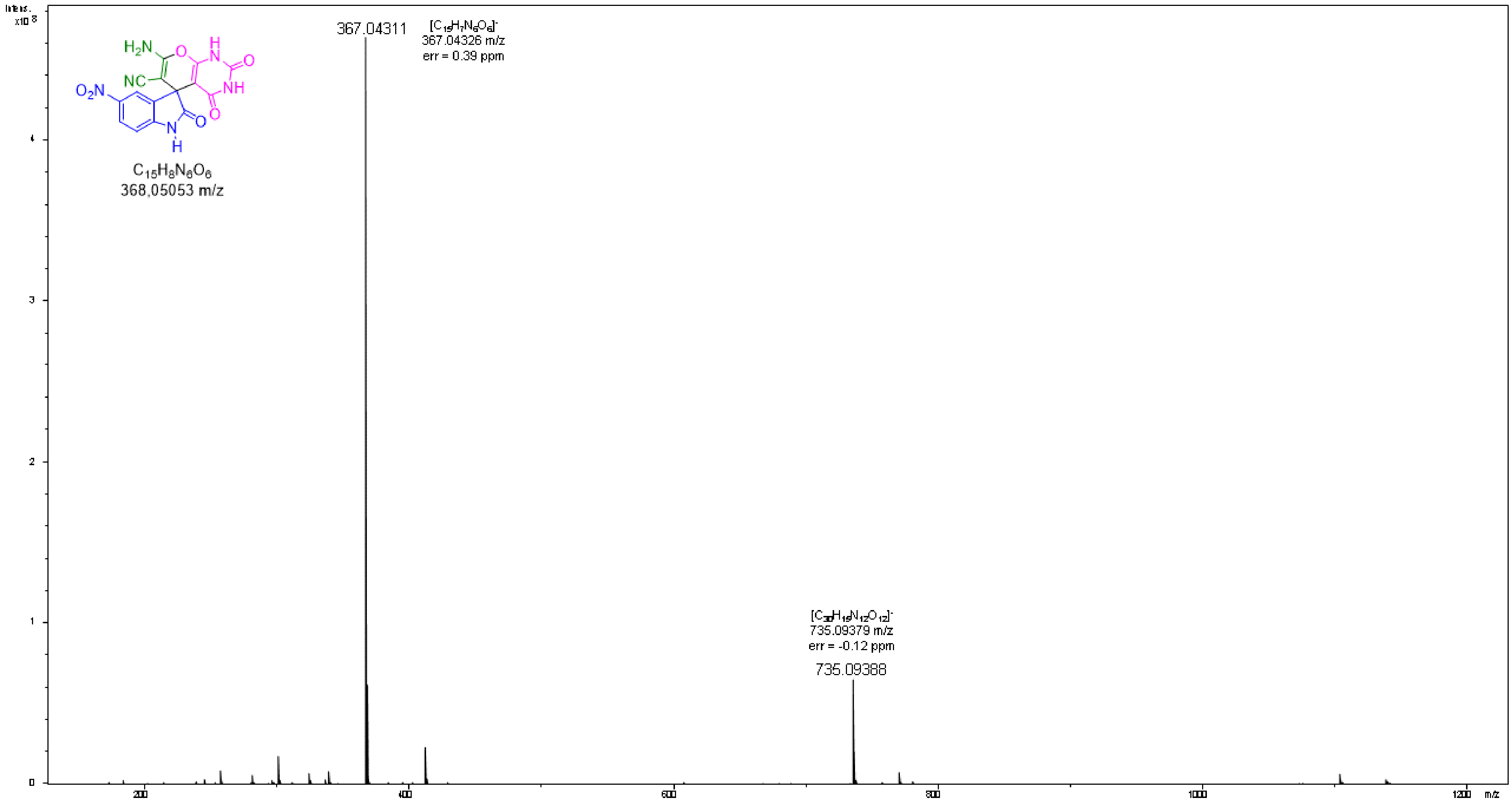

Figure S55. Mass spectrum of compound **1g**.

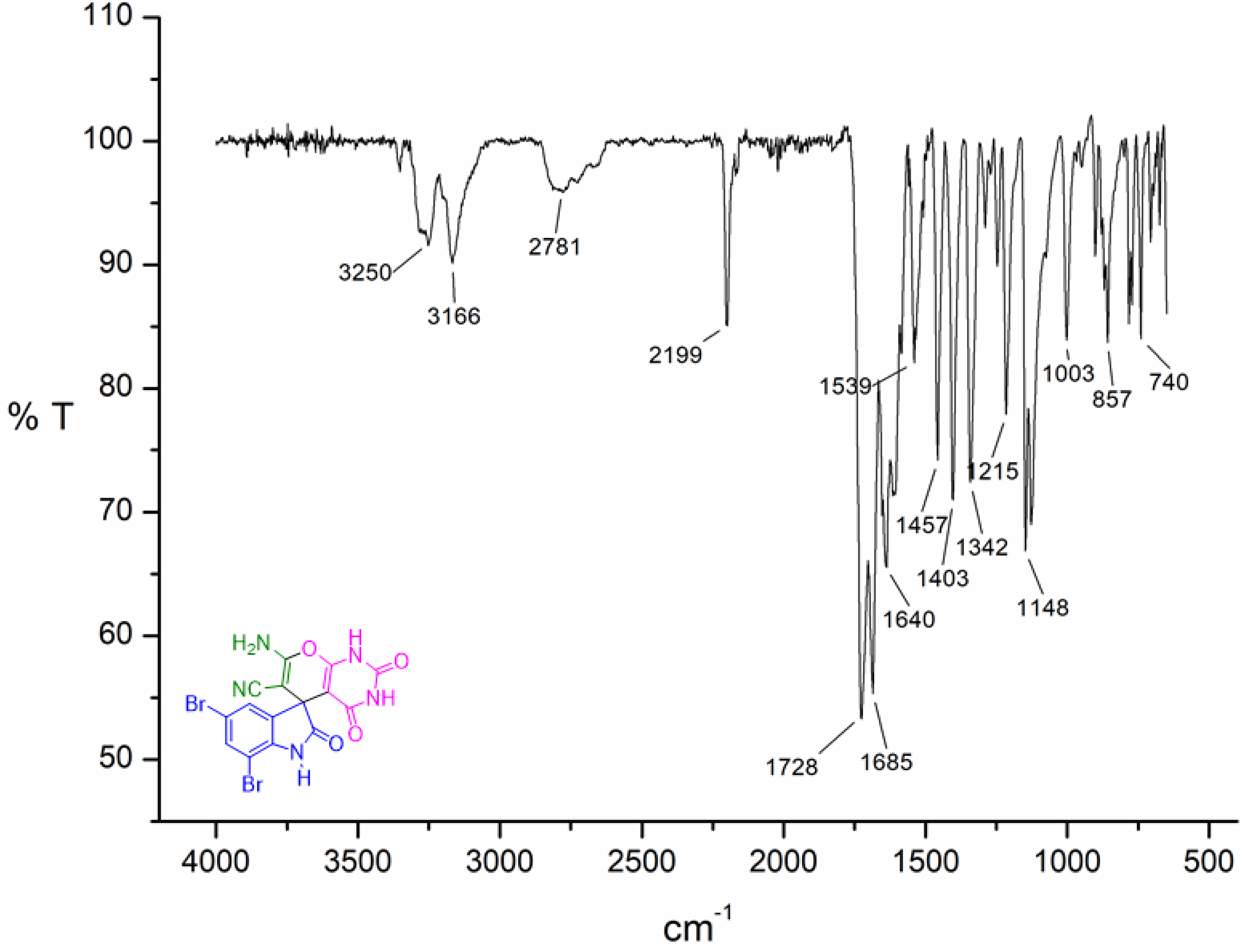

Figure S56. Infrared spectrum of compound **1h**.

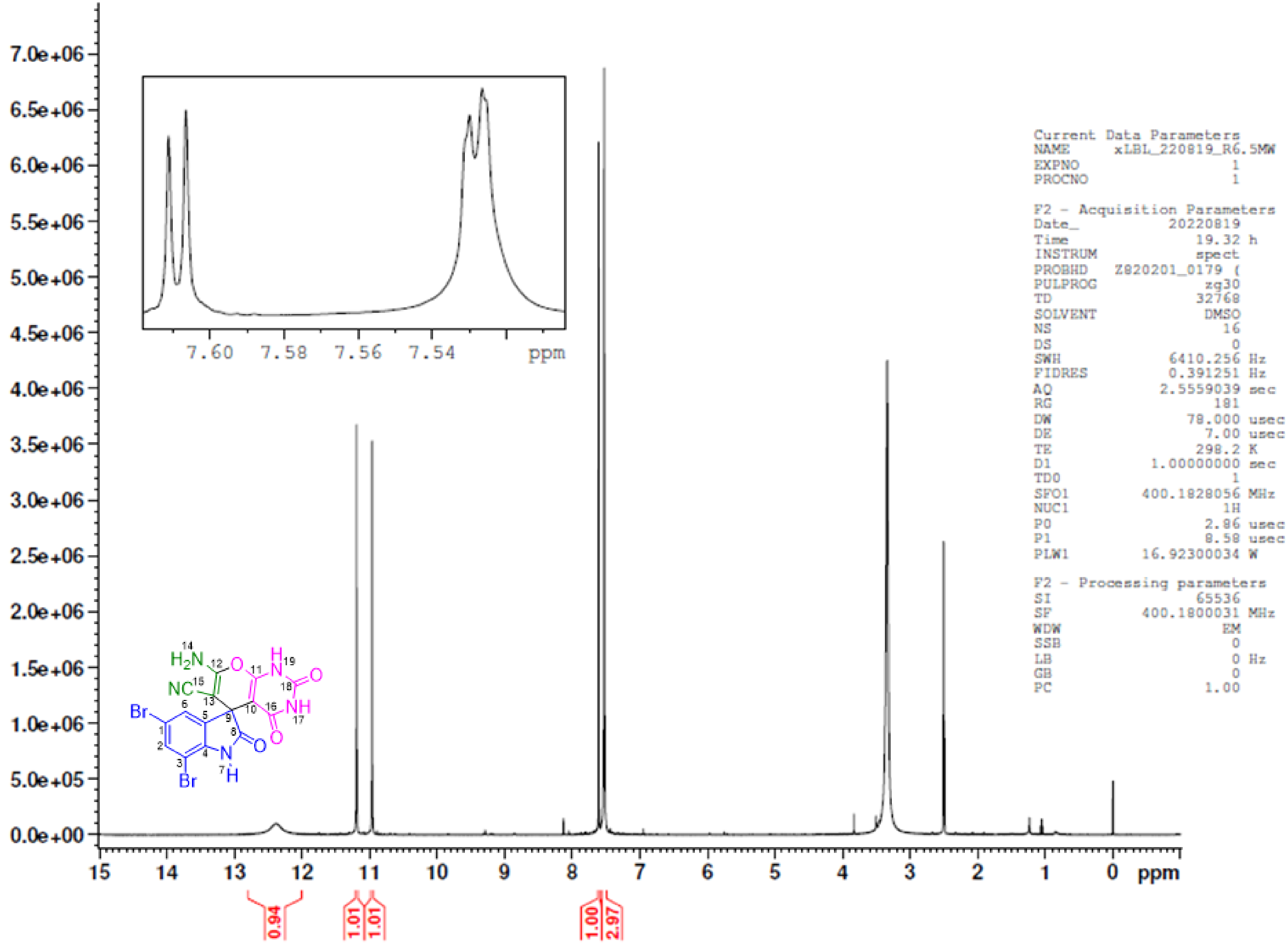

Figure S57. ^{1}H NMR spectrum of compound **1h**.

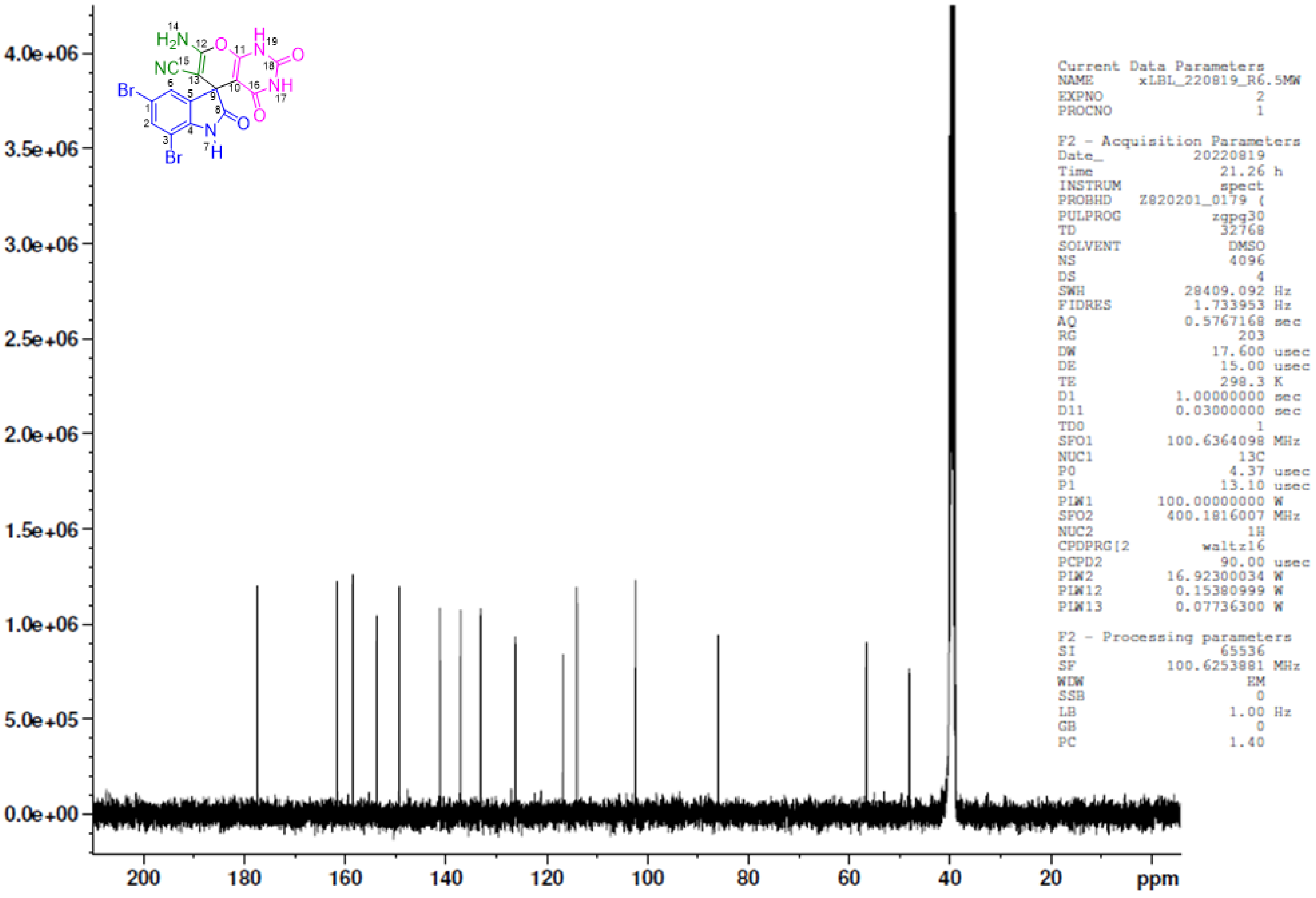

Figure S58. ^{13}C NMR spectrum of compound **1h**.

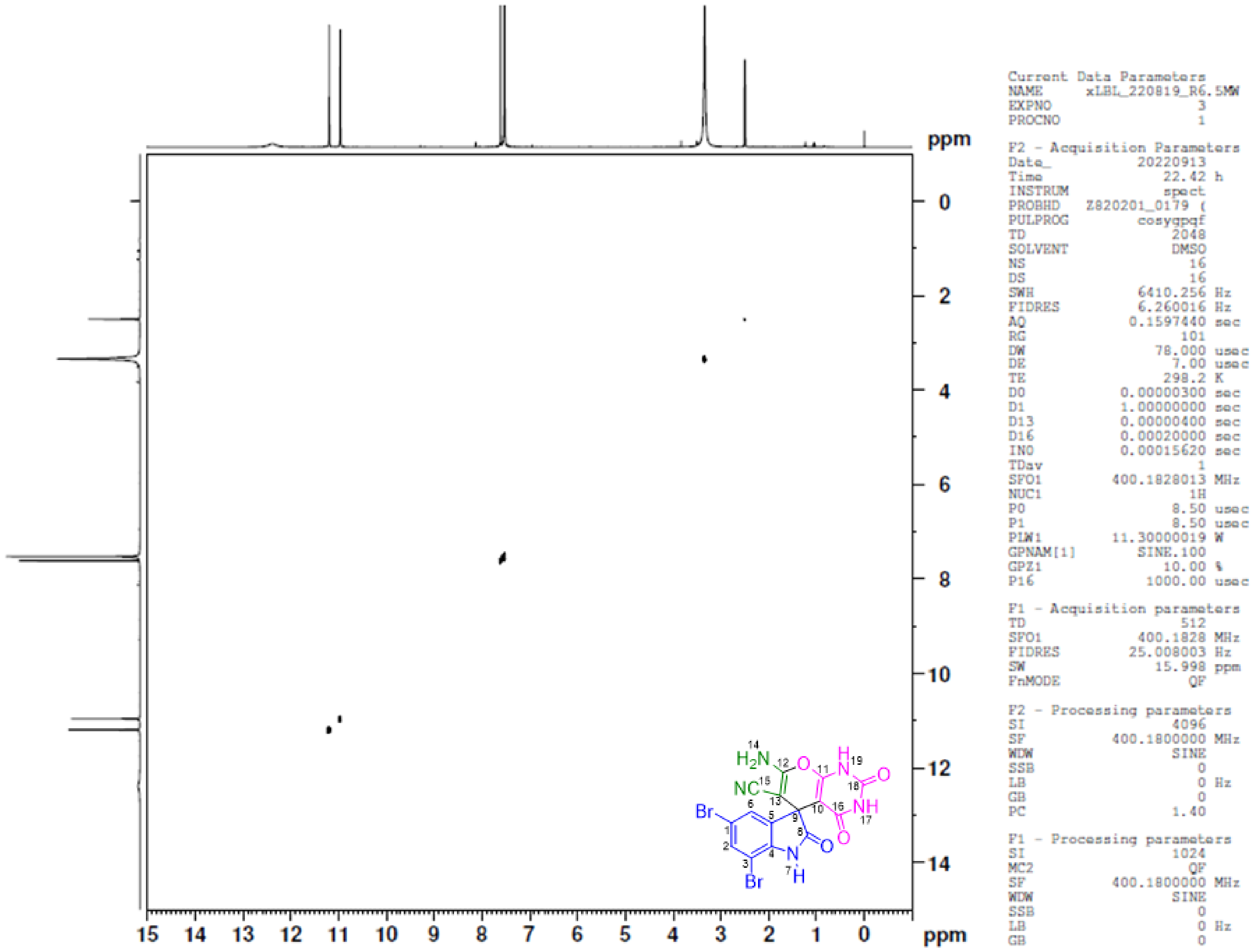

Figure S59. ^{1}H-^{1}H COSY NMR spectrum of compound **1h**.

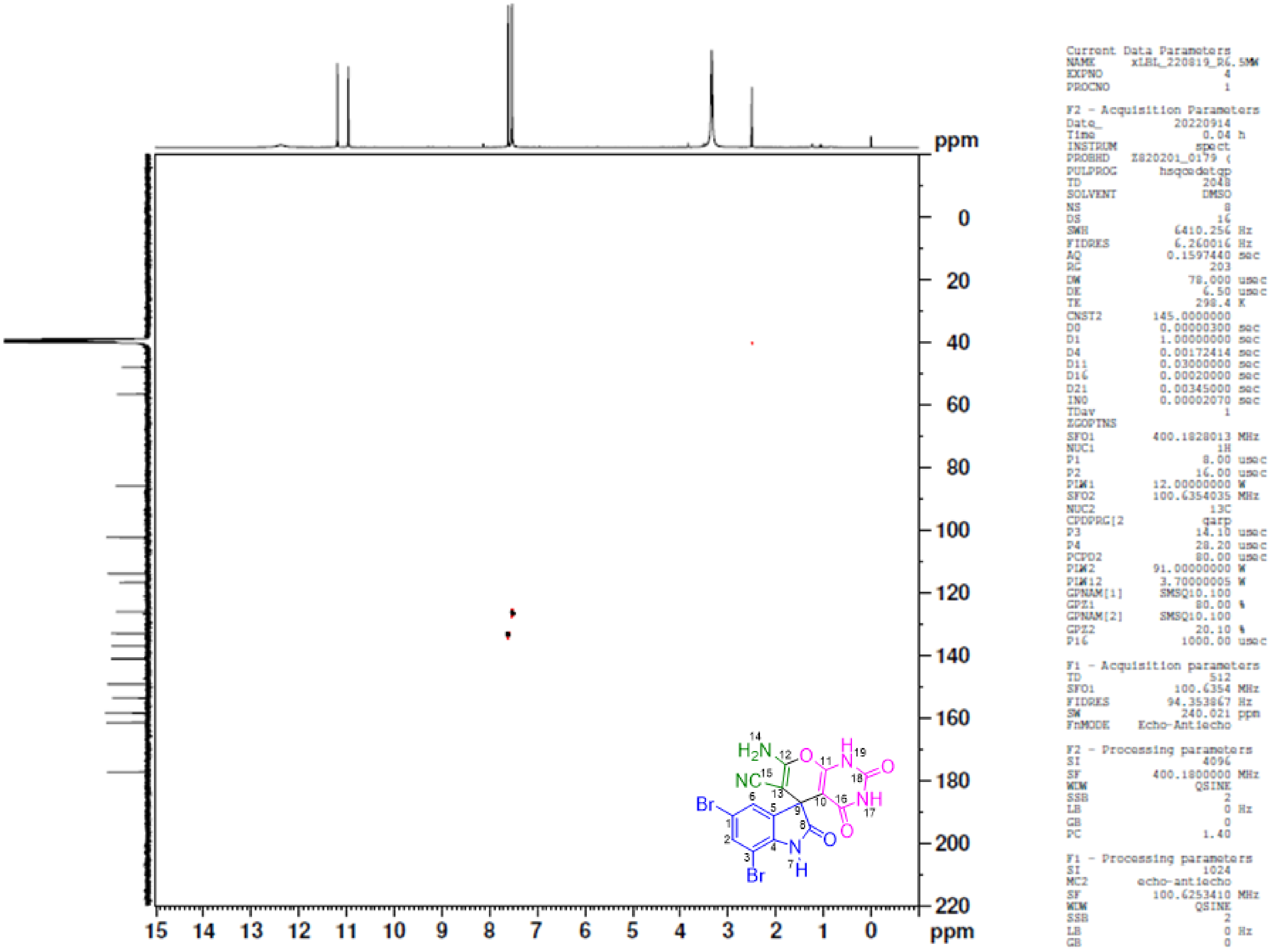

Figure S60. ^{1}H-^{13}C HSQC NMR spectrum of compound **1h**.

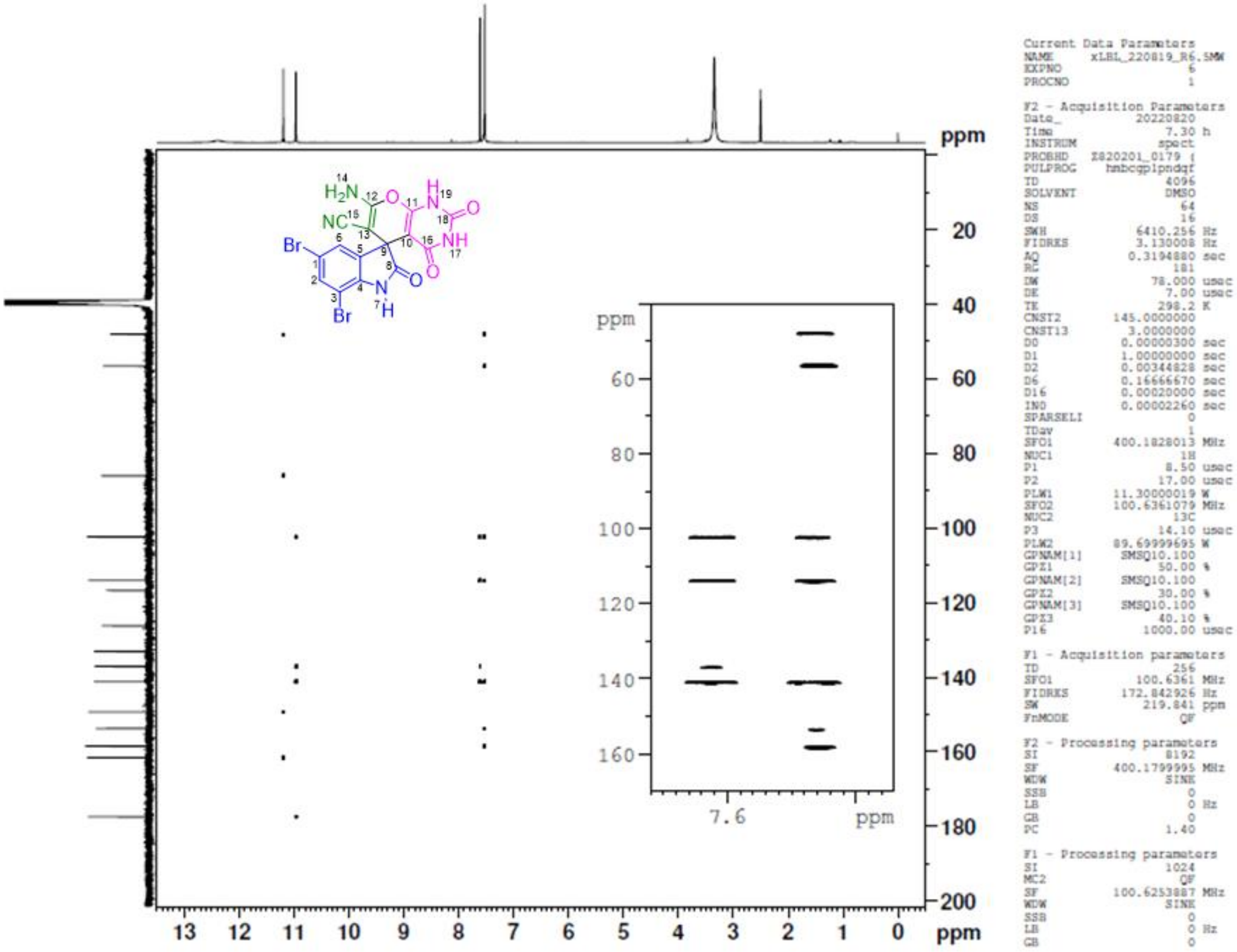

Figure S61. ^{1}H-^{13}C HMBC NMR spectrum of compound **1h** (cnst13 = 3 Hz).

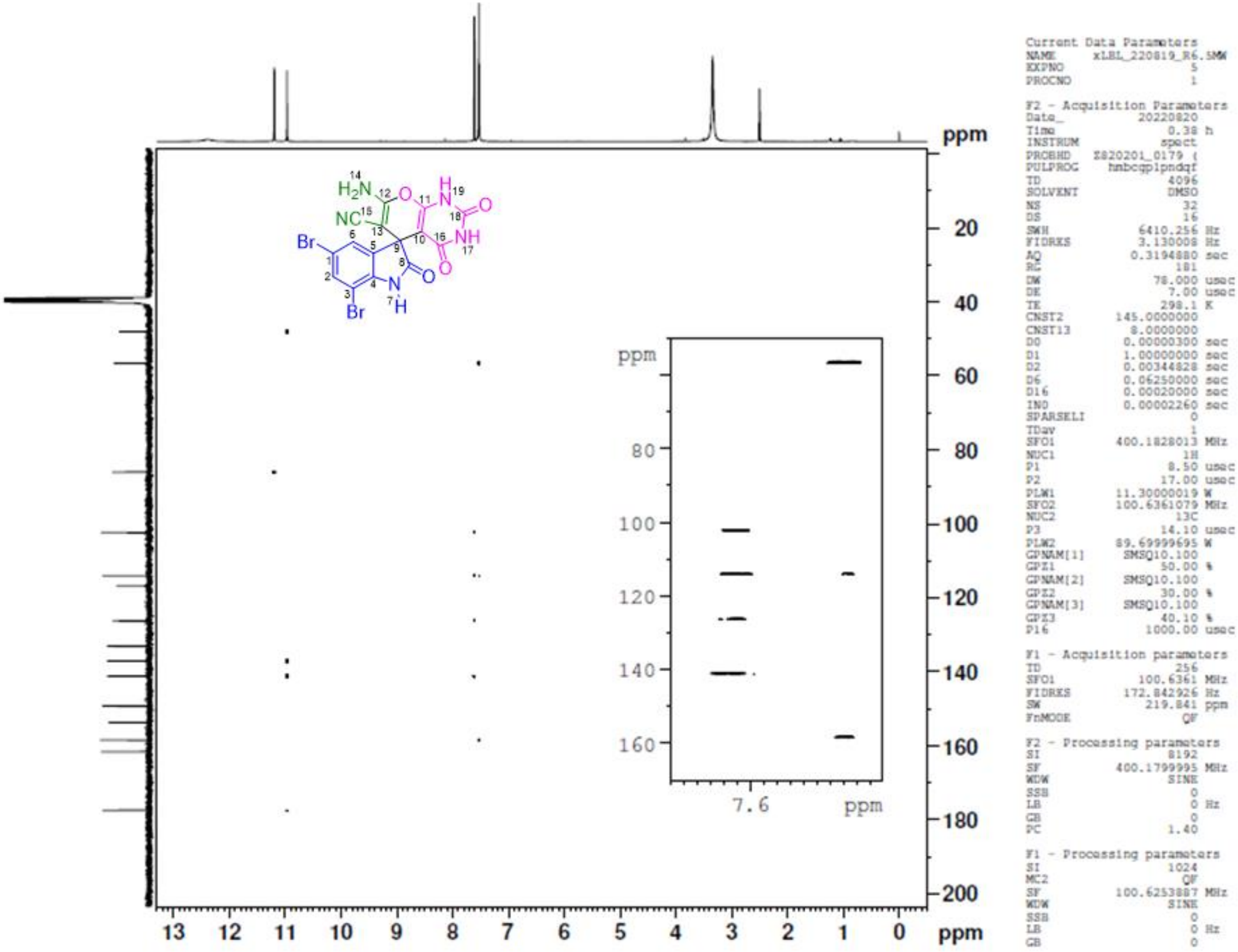

Figure S62. ^{1}H-^{13}C HMBC NMR spectrum of compound **1h** (cnst13 = 8 Hz).

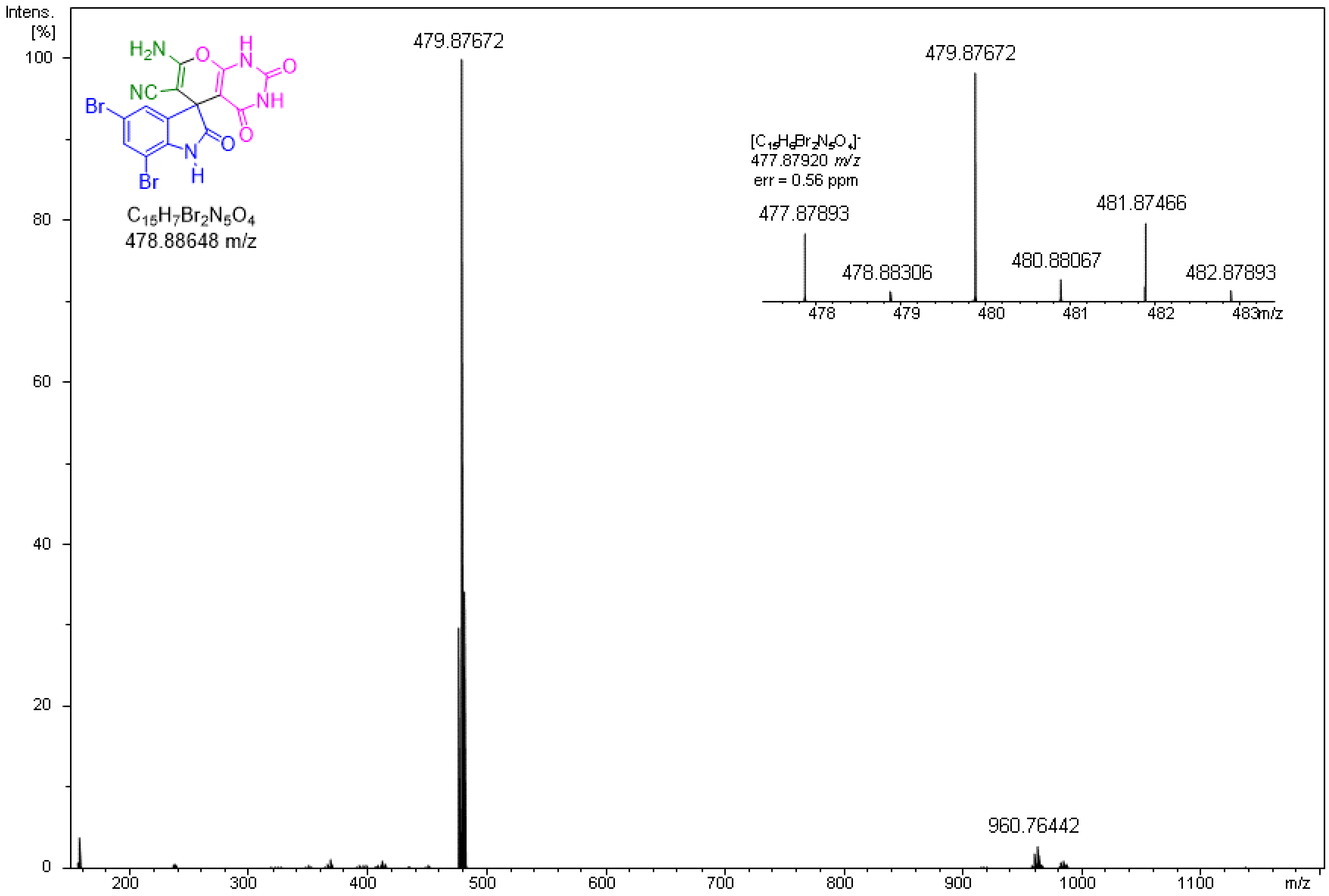

Figure S63. Mass spectrum of compound **1h**.

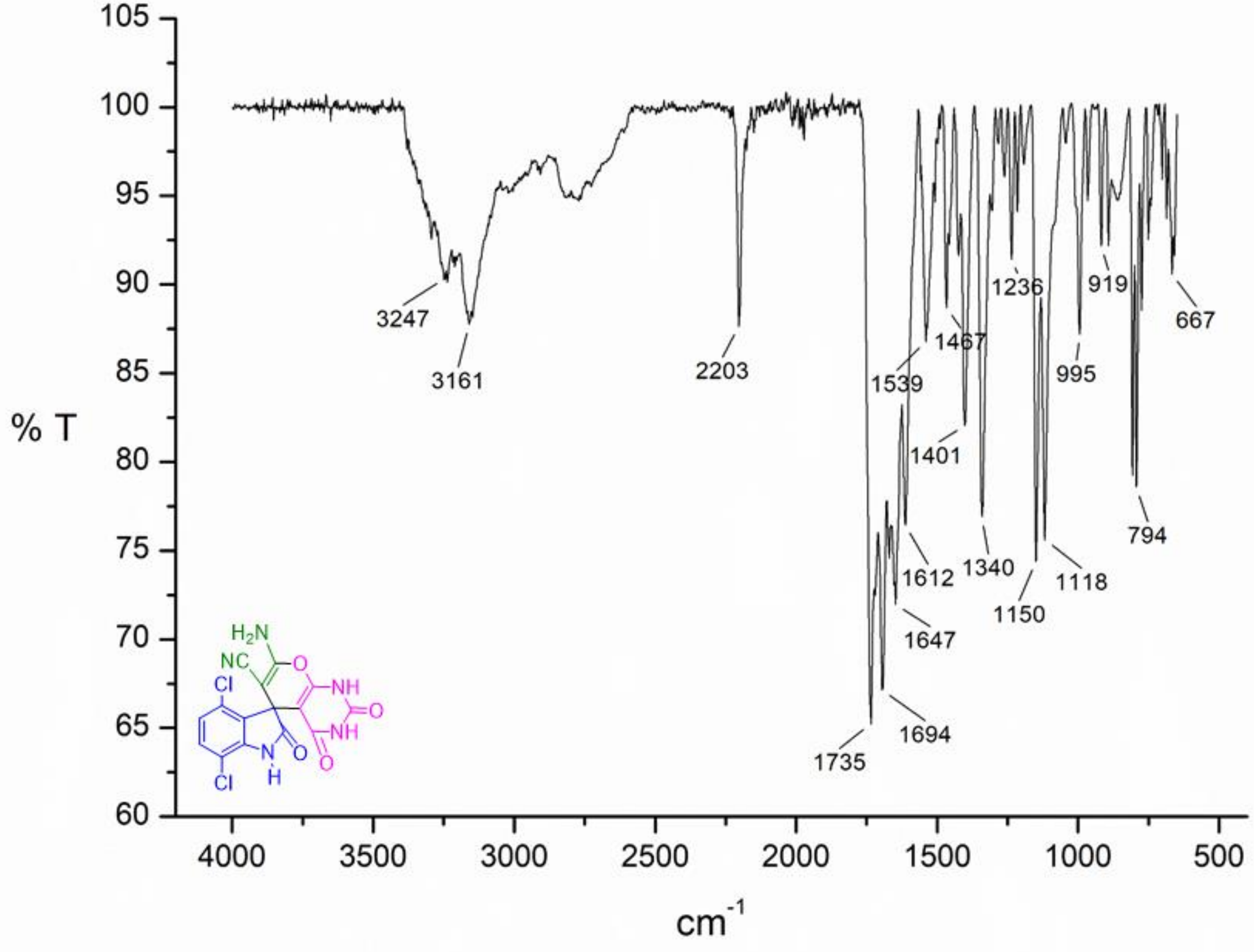

Figure S64. Infrared spectrum of compound **1i**.

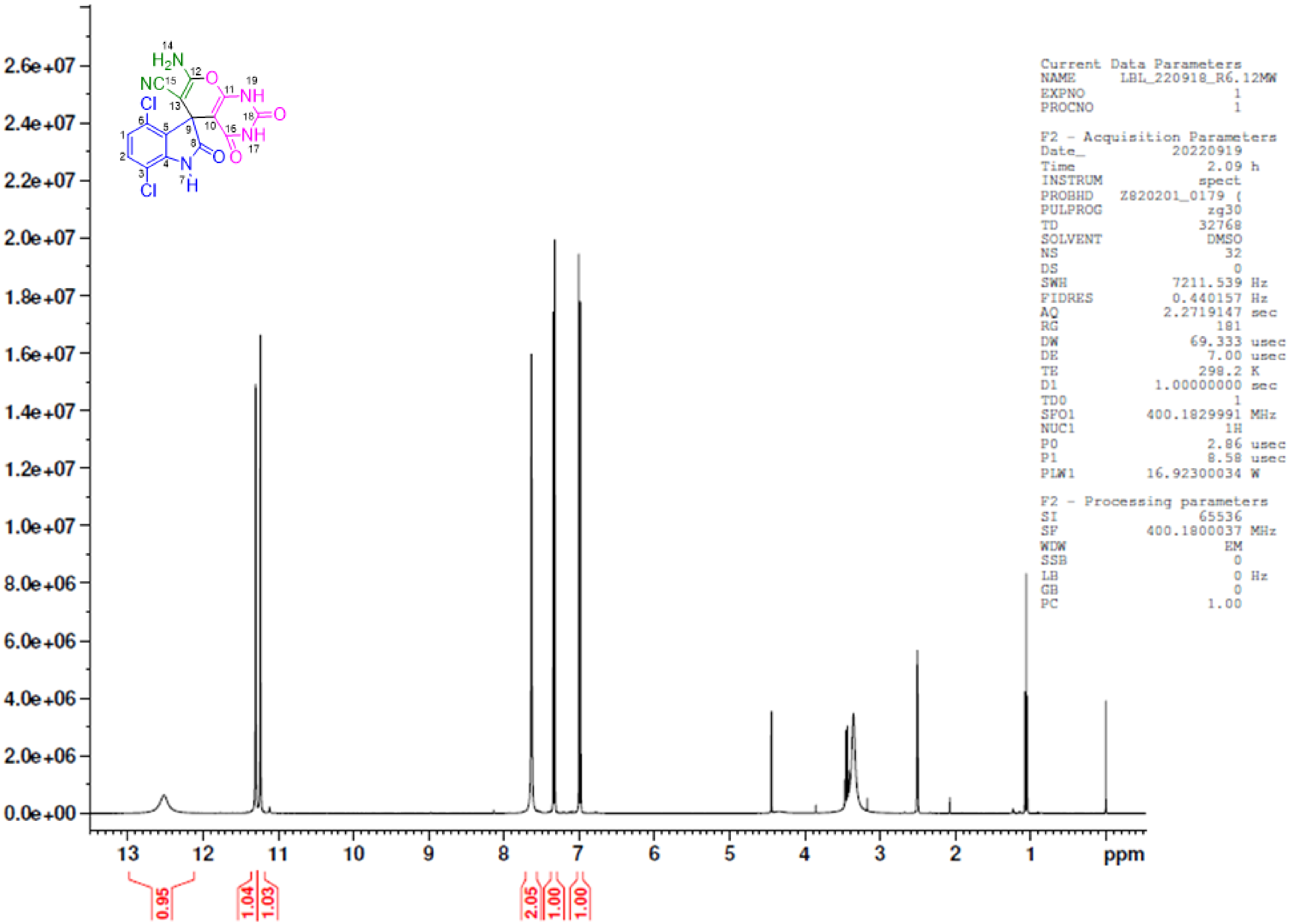

Figure S65. ^{1}H NMR spectrum of compound **1i**.

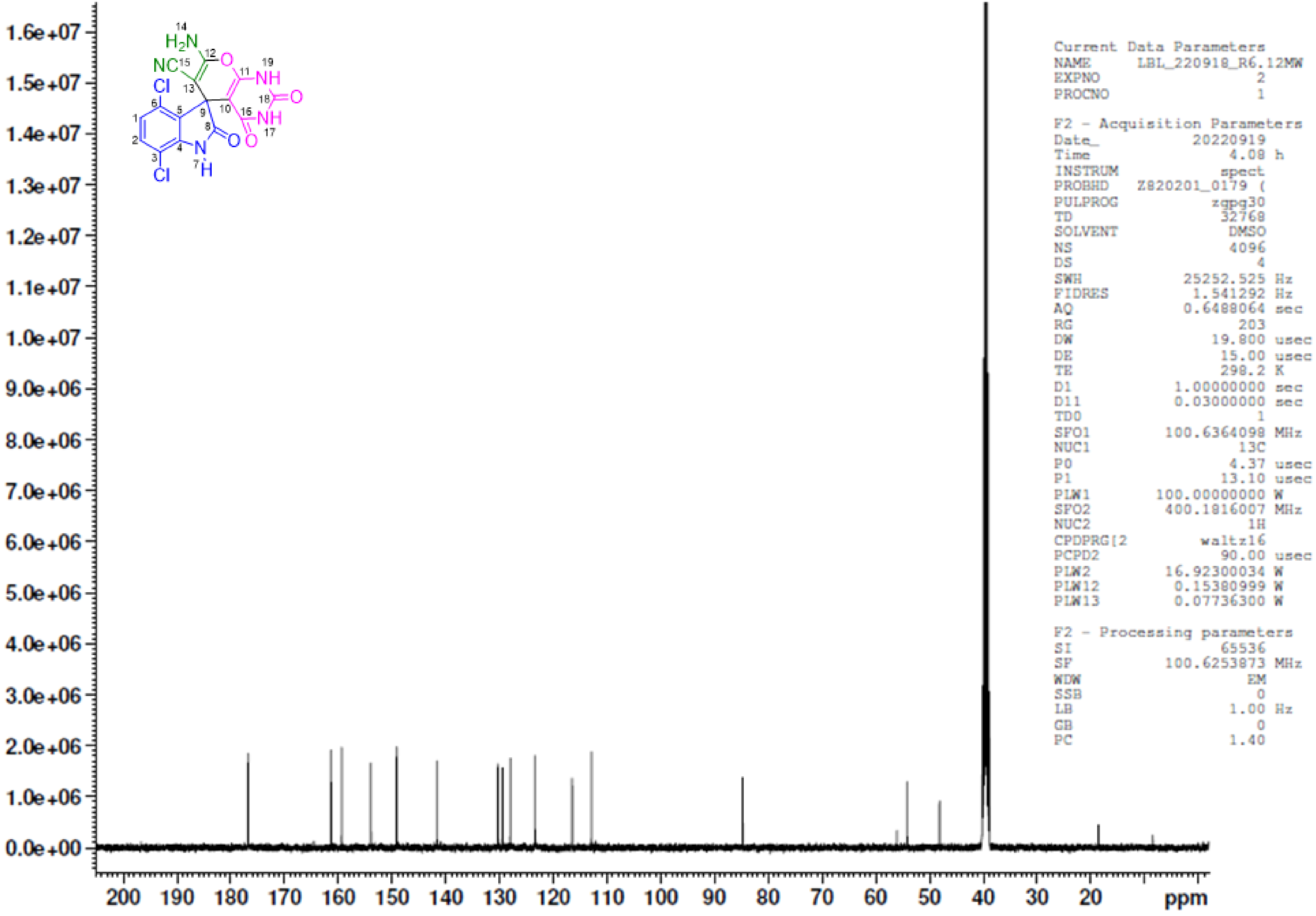

Figure S66. ^{13}C NMR spectrum of compound **1i**.

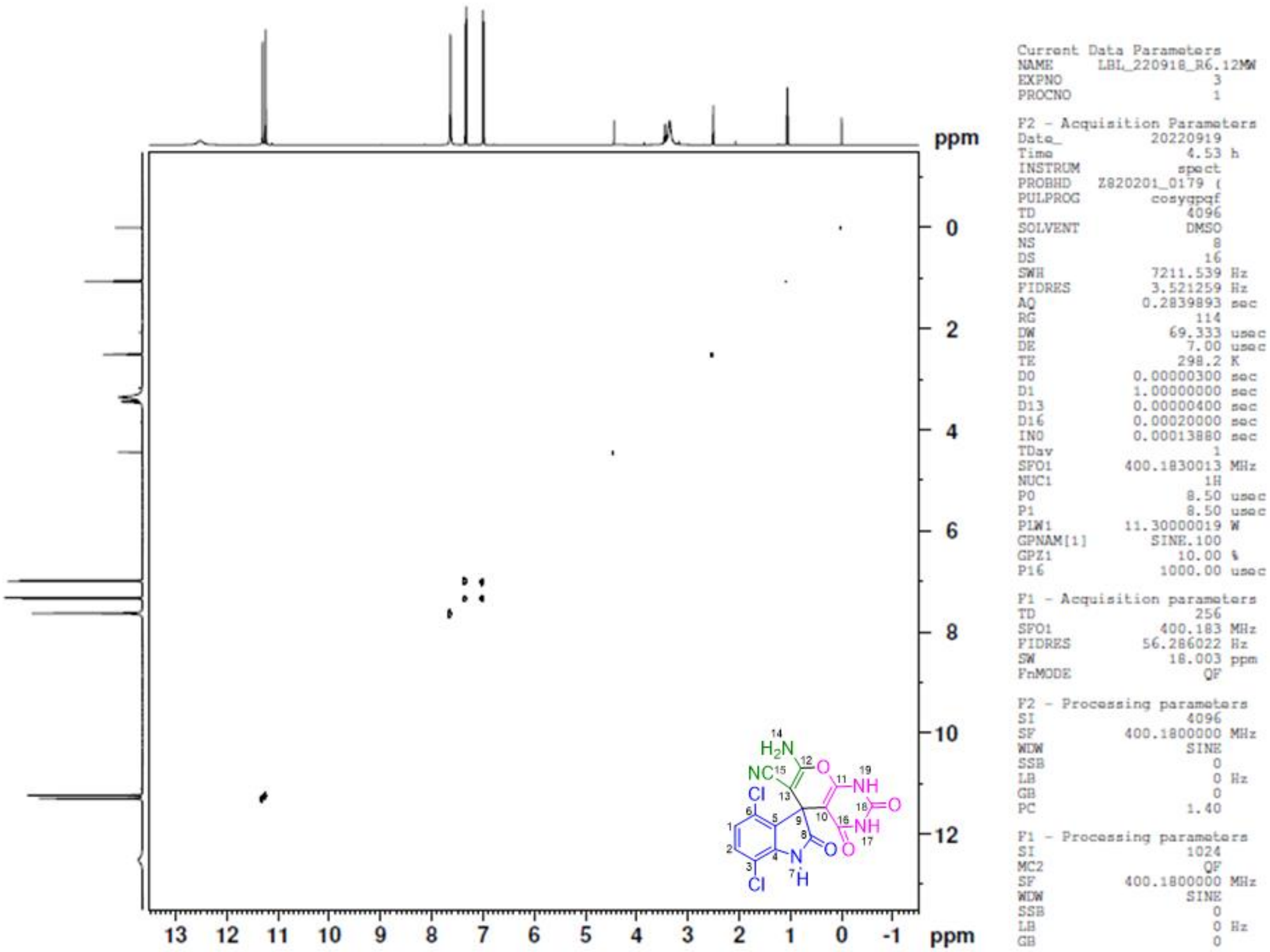

Figure S67. ^1H-^1H COSY NMR spectrum of compound **1i**.

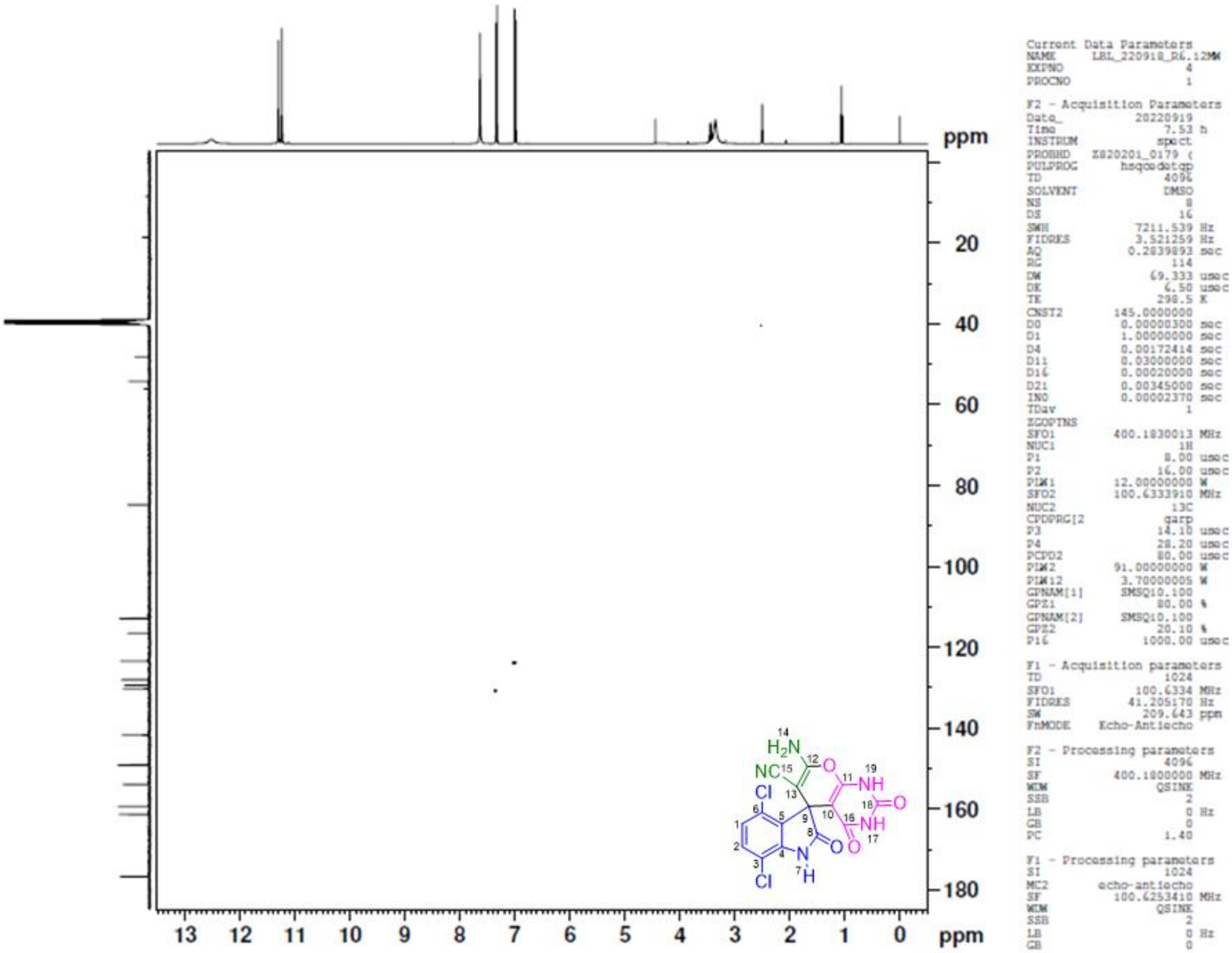

Figure S68. ^{1}H-^{13}C HSQC NMR spectrum of compound **1i**.

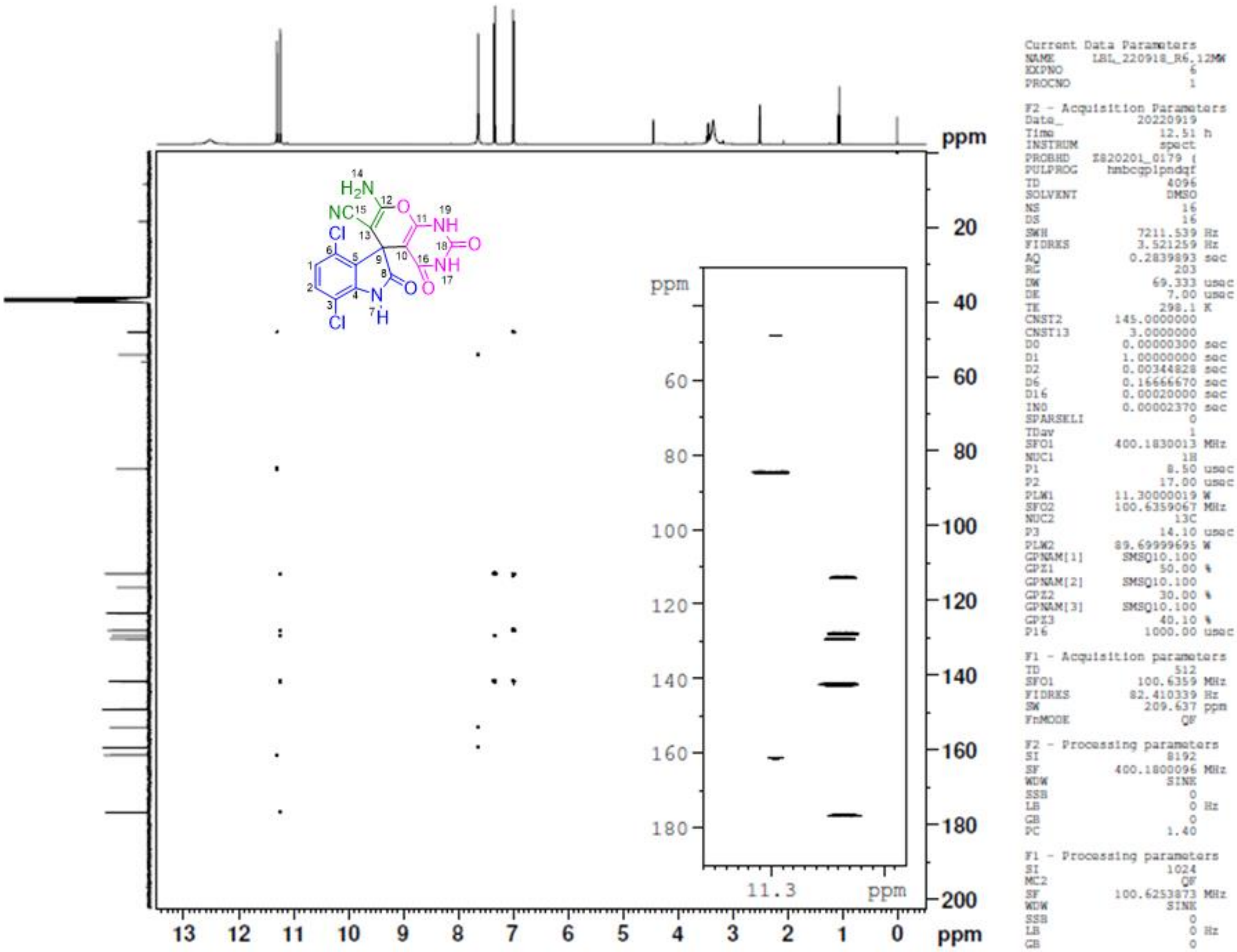

Figure S69. ^{1}H-^{13}C HMBC NMR spectrum of compound **1i** (cnst13 = 3 Hz).

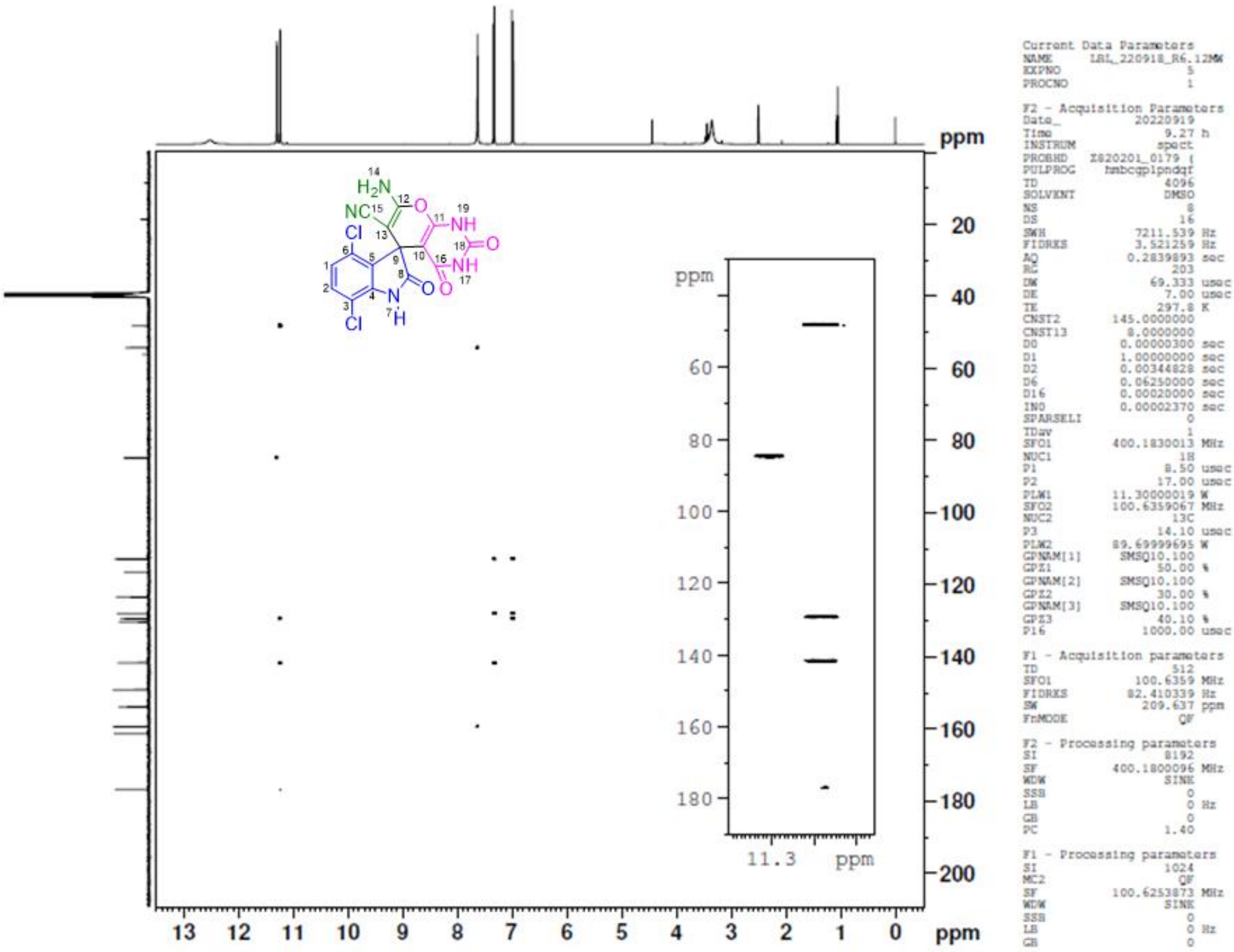

Figure S70. ^{1}H-^{13}C HMBC NMR spectrum of compound **1i** (cnst13 = 8 Hz).

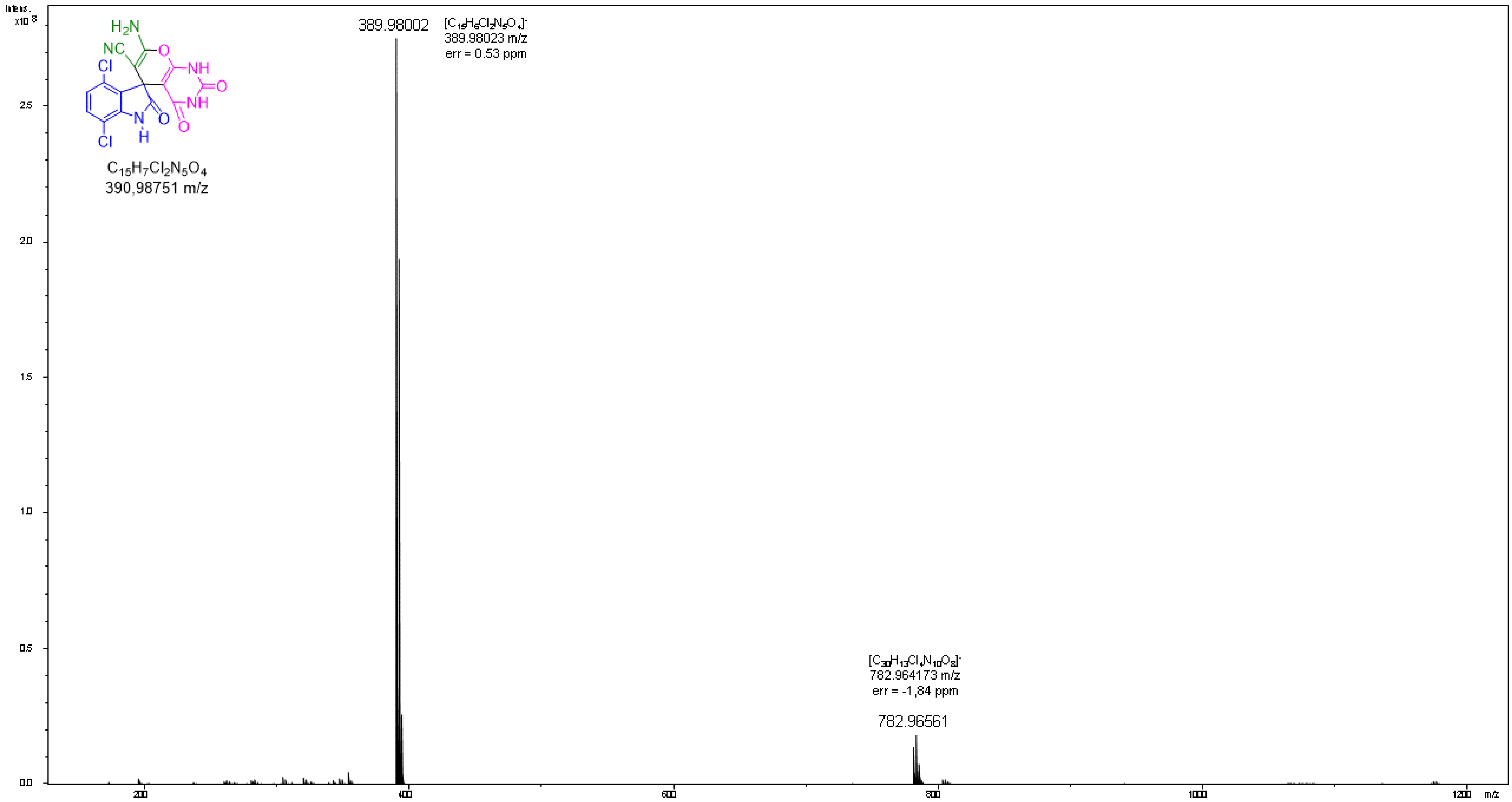

Figure S71. Mass spectrum of compound **1i**.

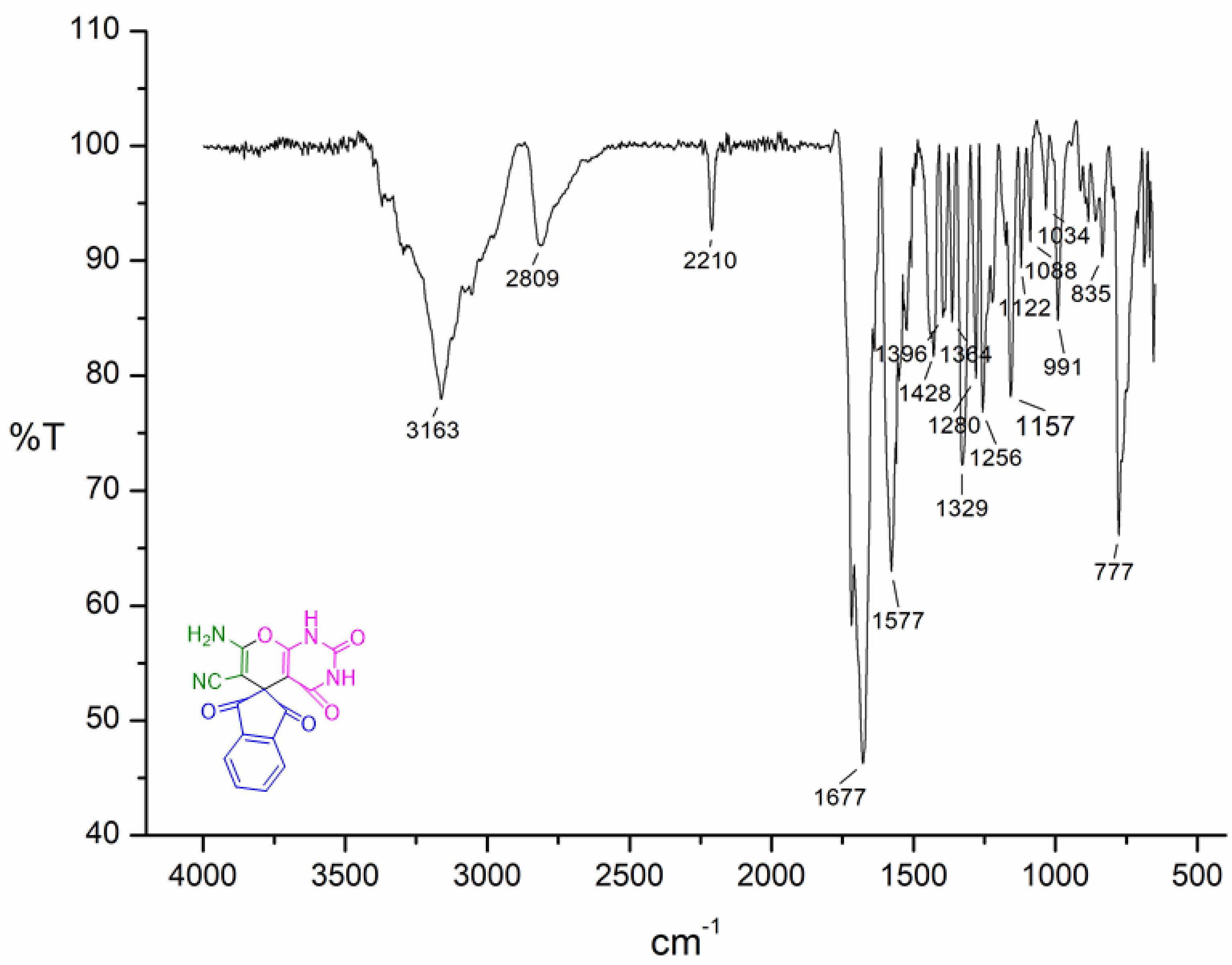

Figure S72. Infrared spectrum of compound **1j**.

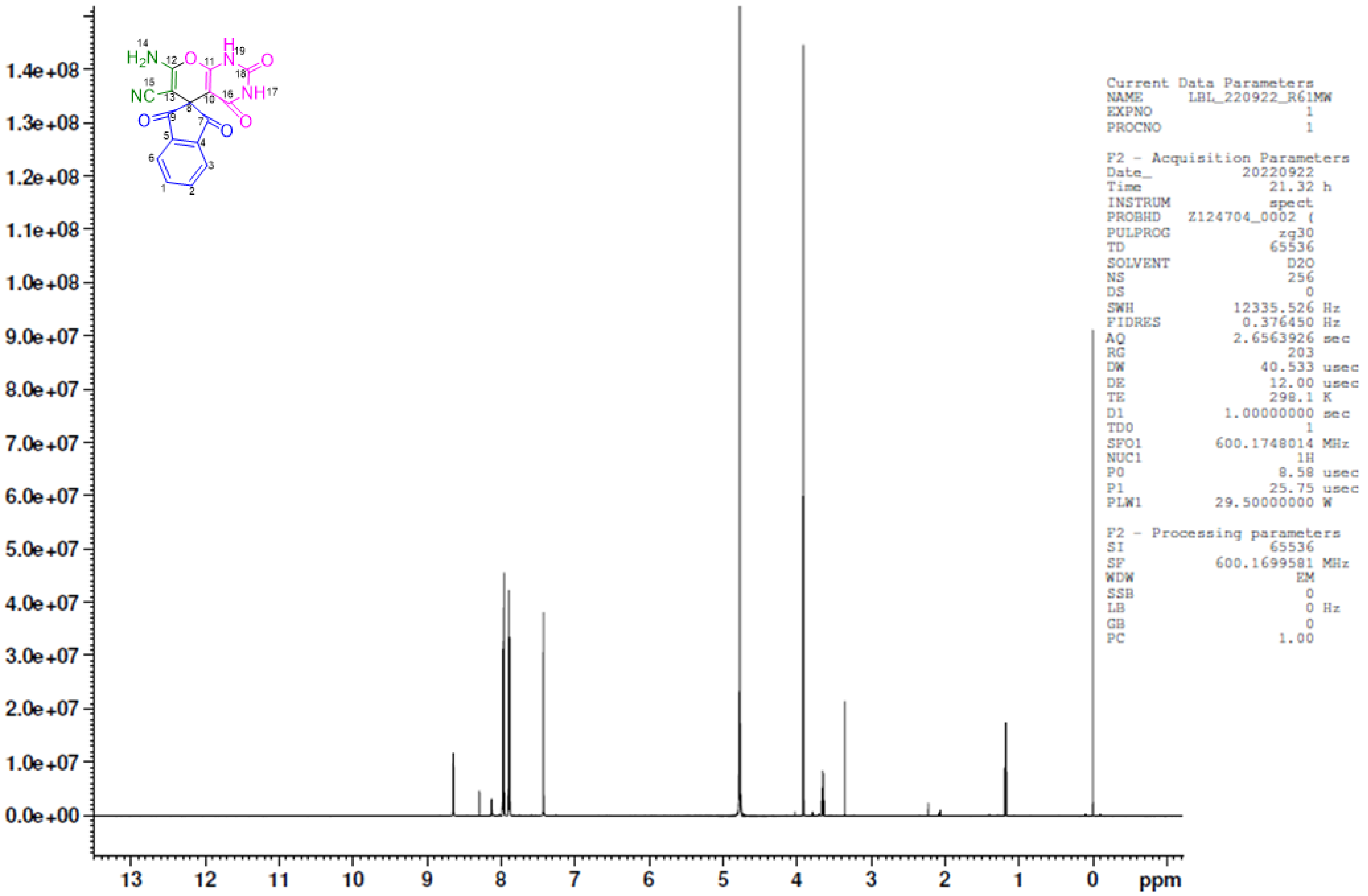

Figure S73. ^{1}H NMR spectrum of compound **1j**.

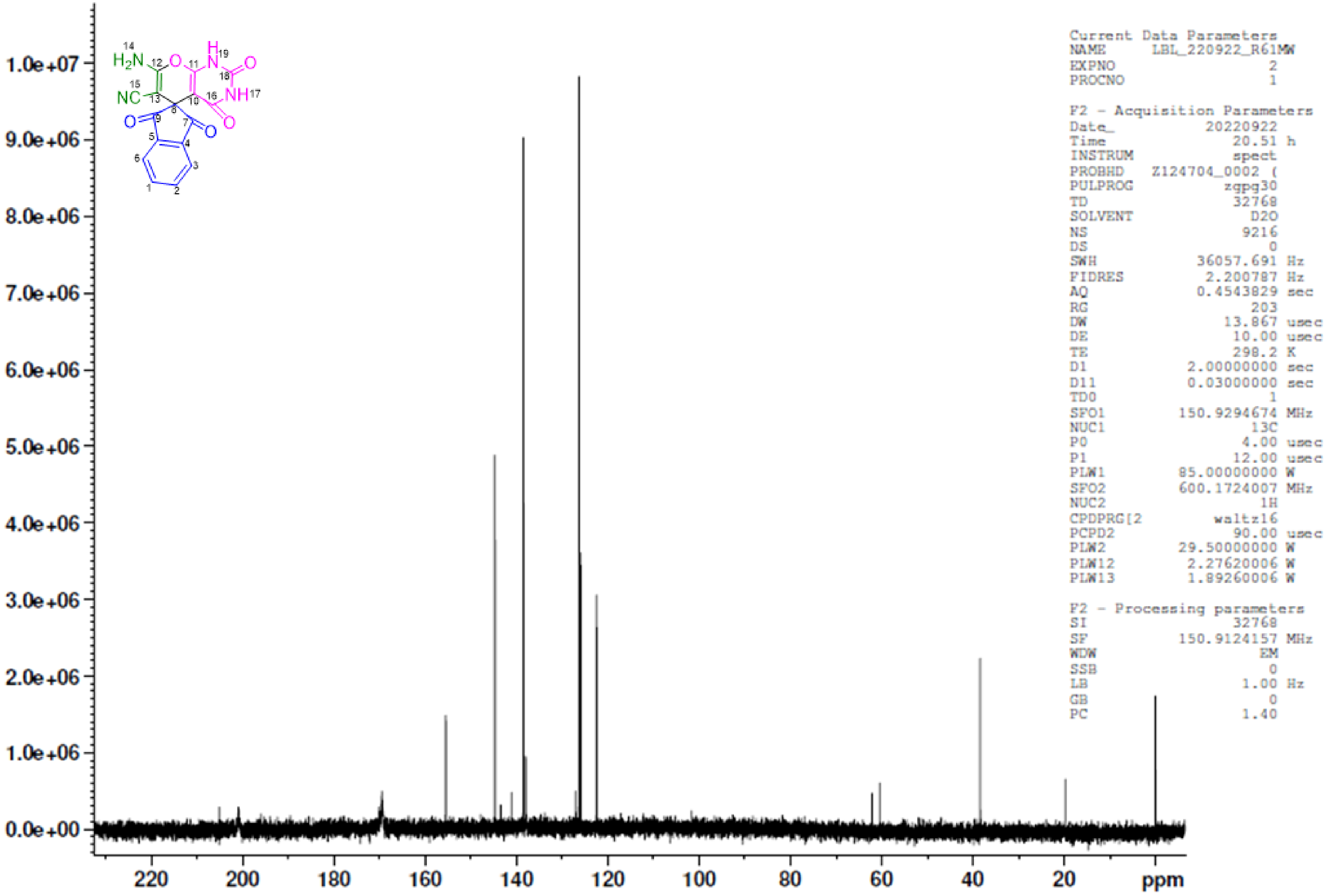

Figure S74. ^{13}C NMR spectrum of compound **1j**.

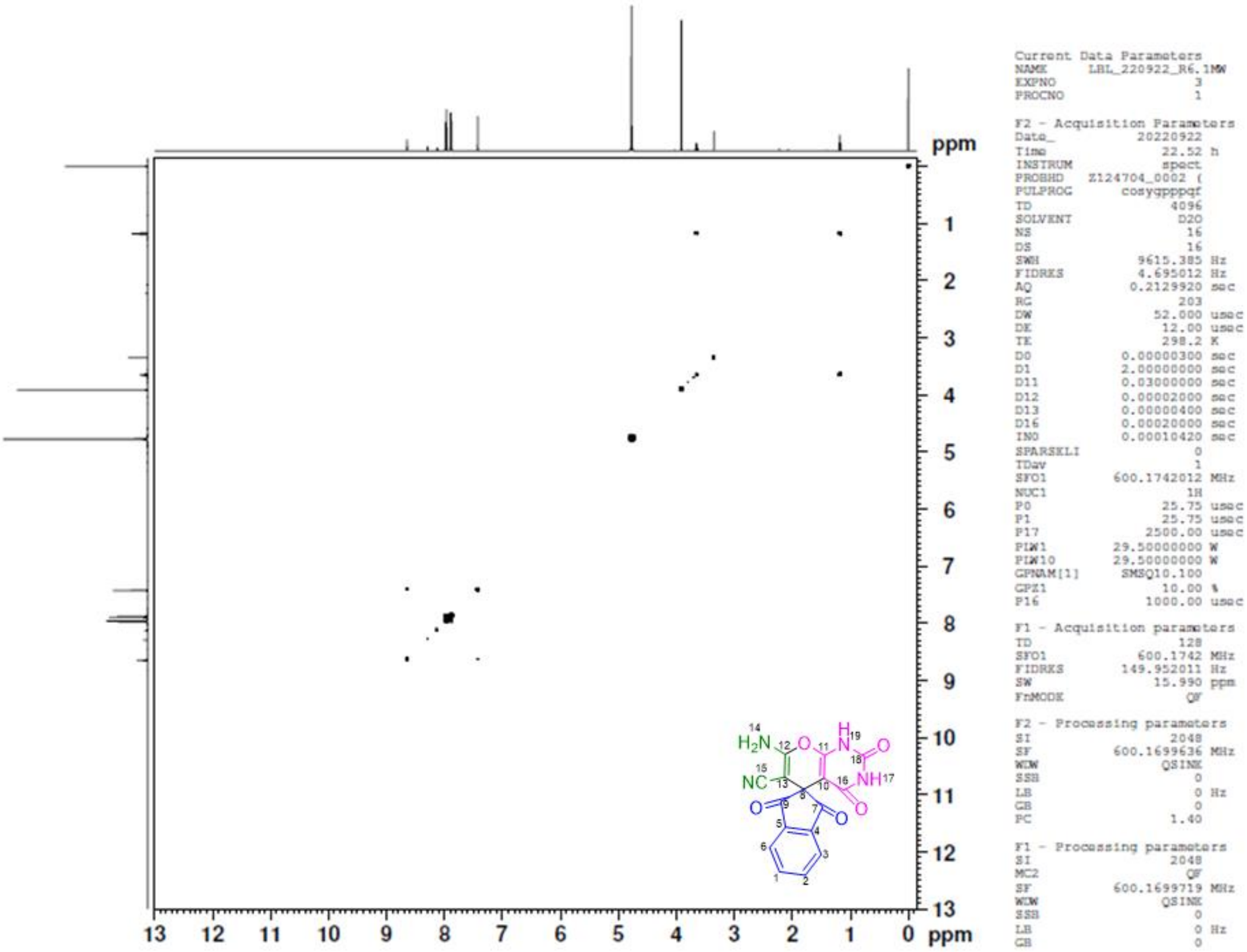

Figure S75. ^1H-^1H COSY NMR spectrum of compound **1j**.

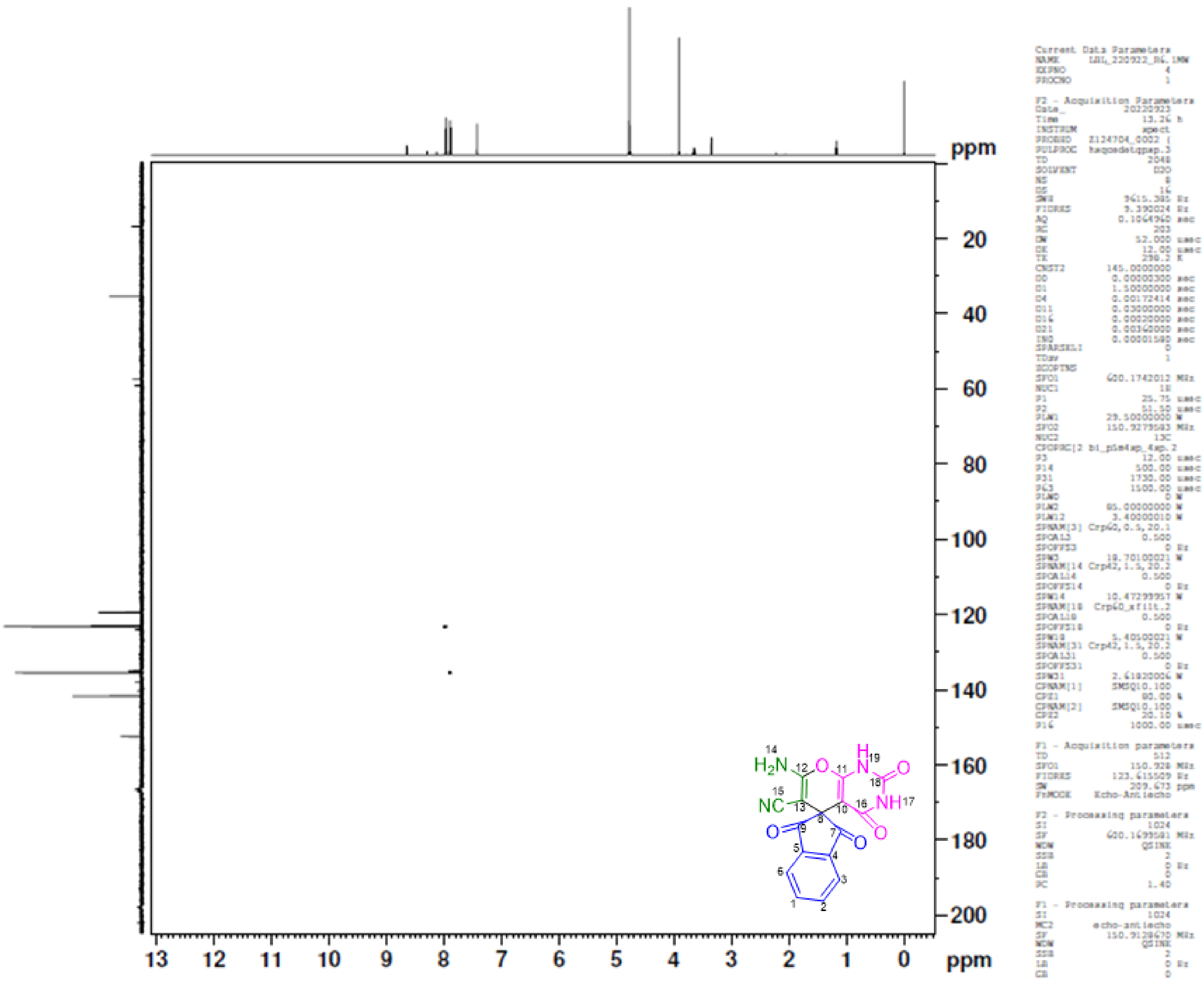

Figure S76. ^{1}H-^{13}C HSQC NMR spectrum of compound **1j**.

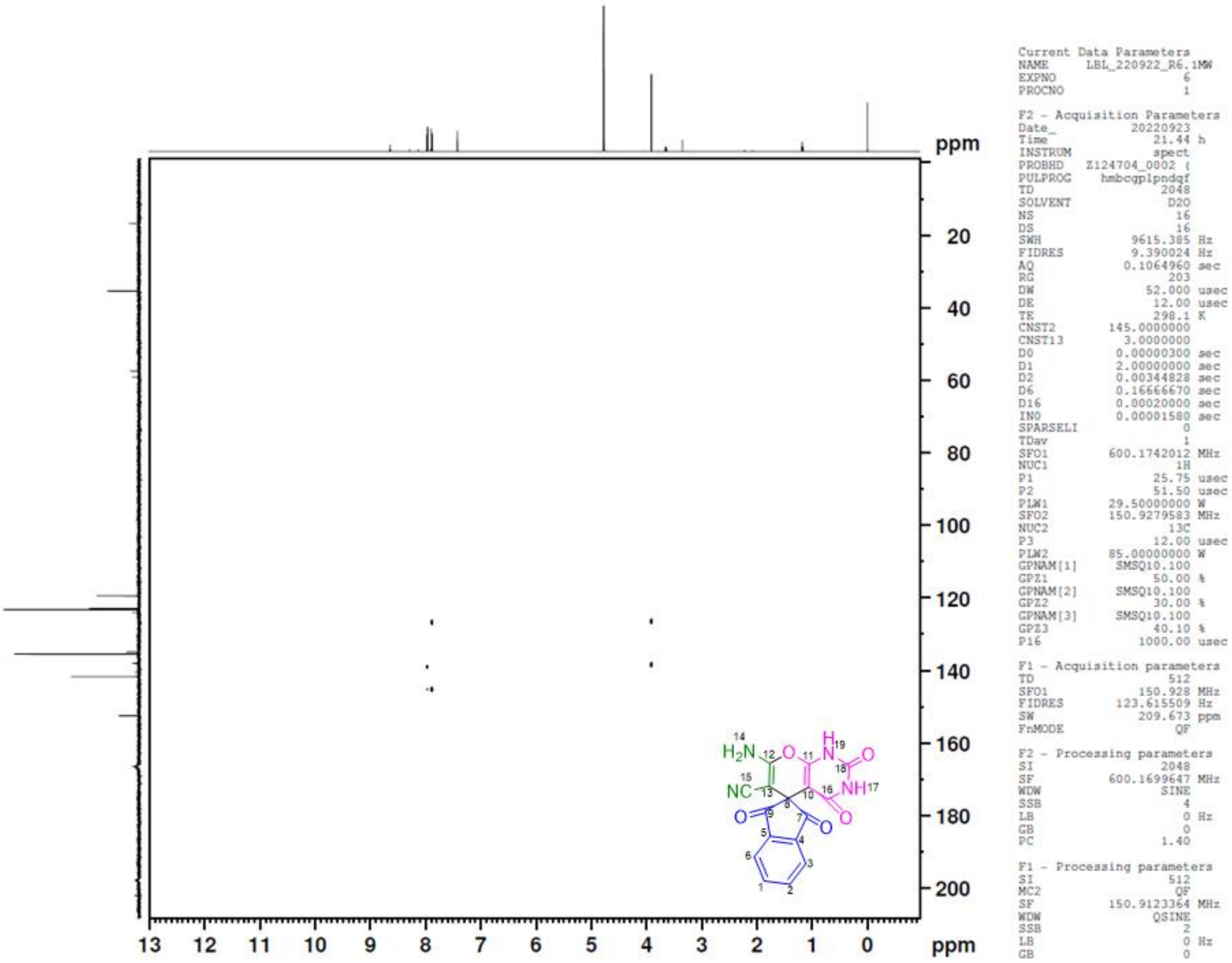

Figure S77. ^{1}H-^{13}C HMBC NMR spectrum of compound **1j** (cnst13 = 3 Hz).

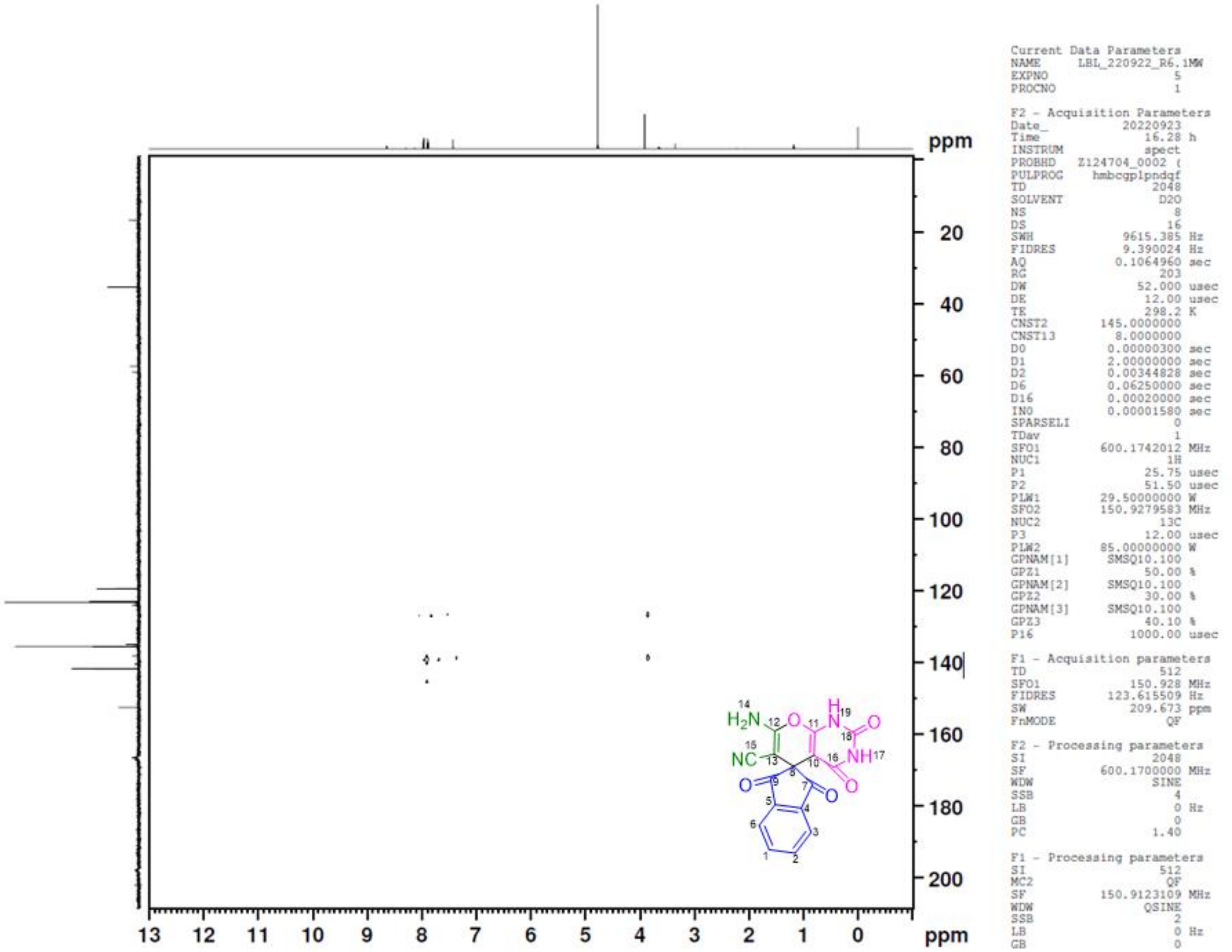

Figure S78. ^{1}H-^{13}C HMBC NMR spectrum of compound **1j** (cnst13 = 8 Hz).

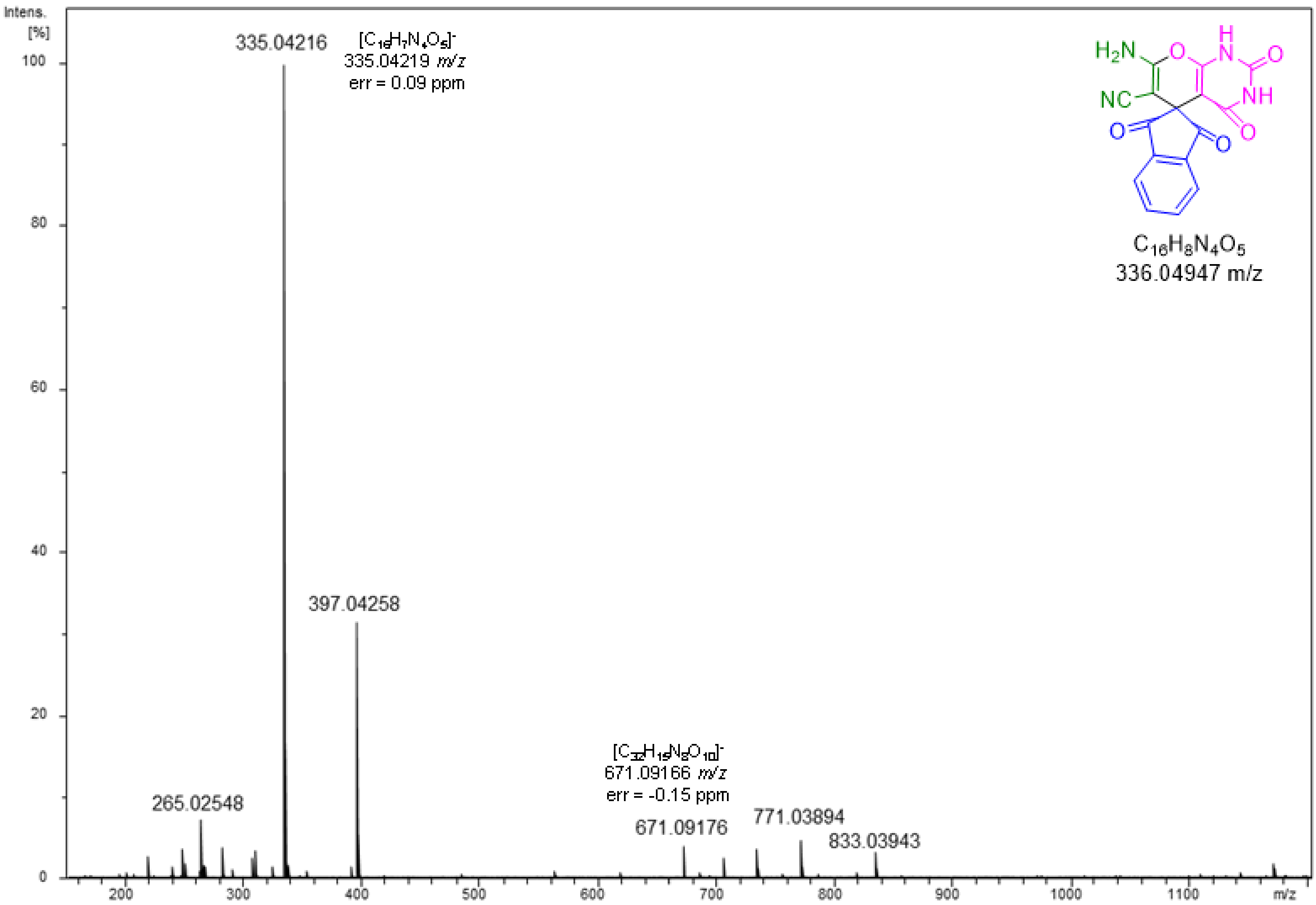

Figure S79. Mass spectrum of compound **1j**.

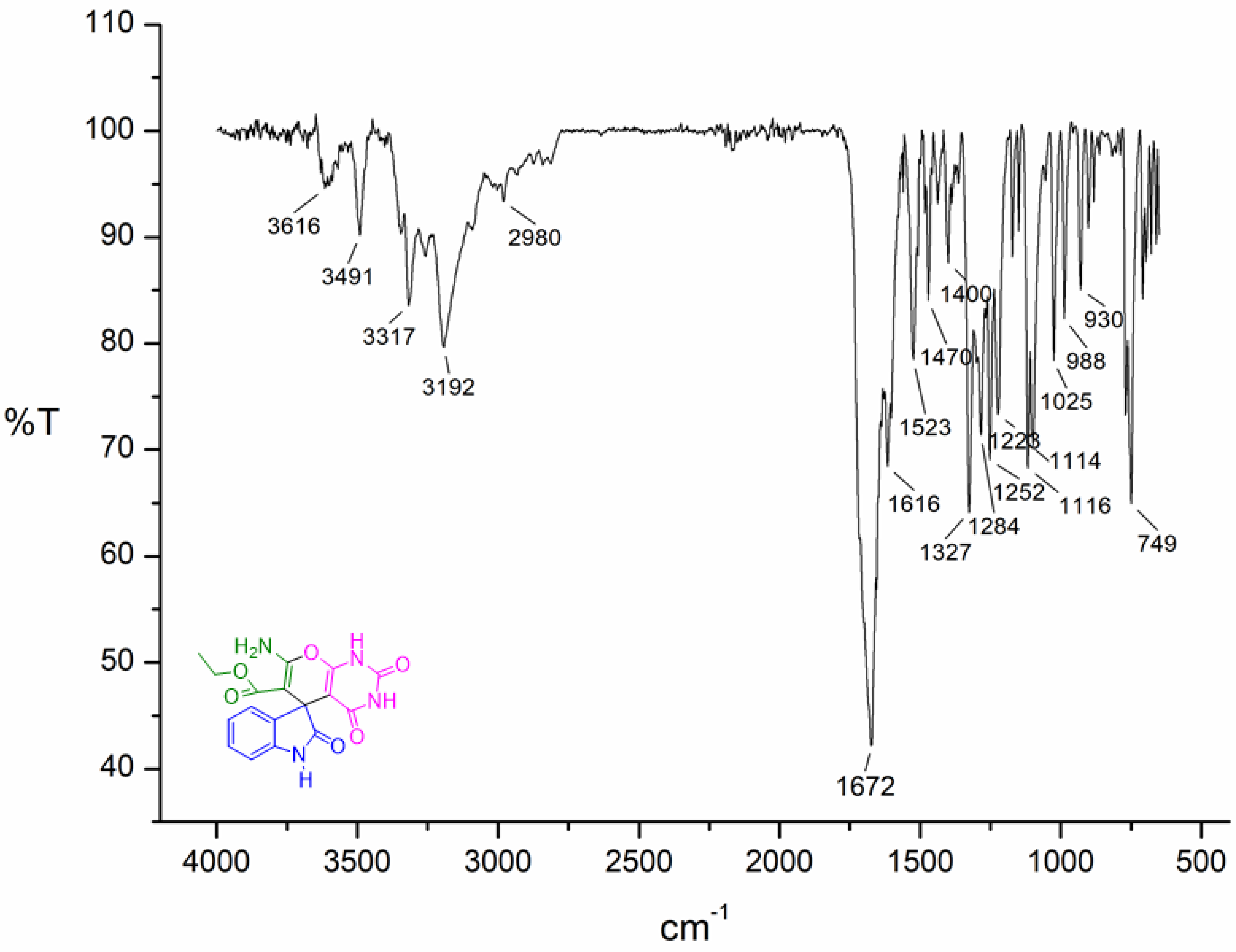

Figure S80. Infrared spectrum of compound **1k**.

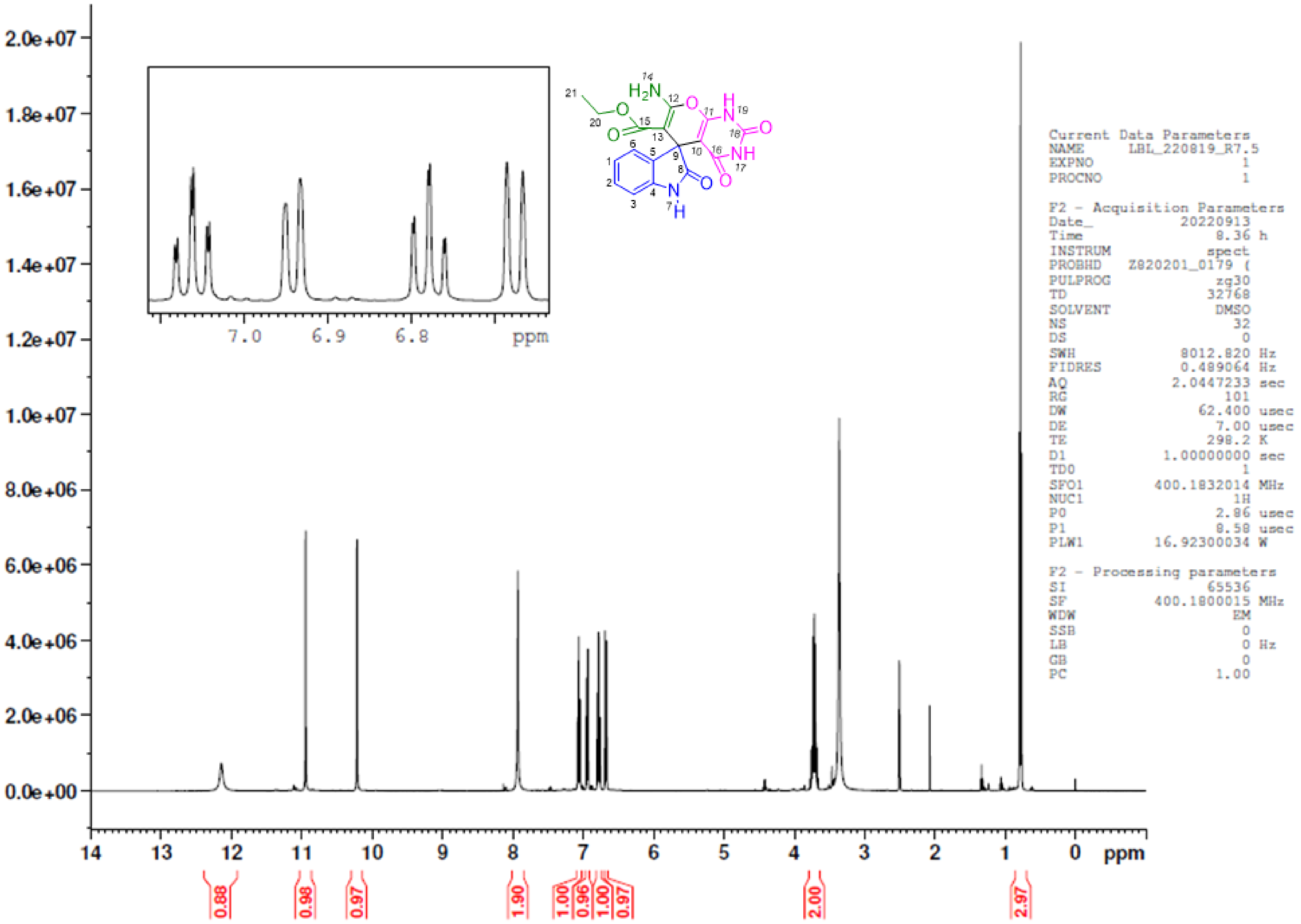

Figure S81. ^{1}H NMR spectrum of compound **1k**.

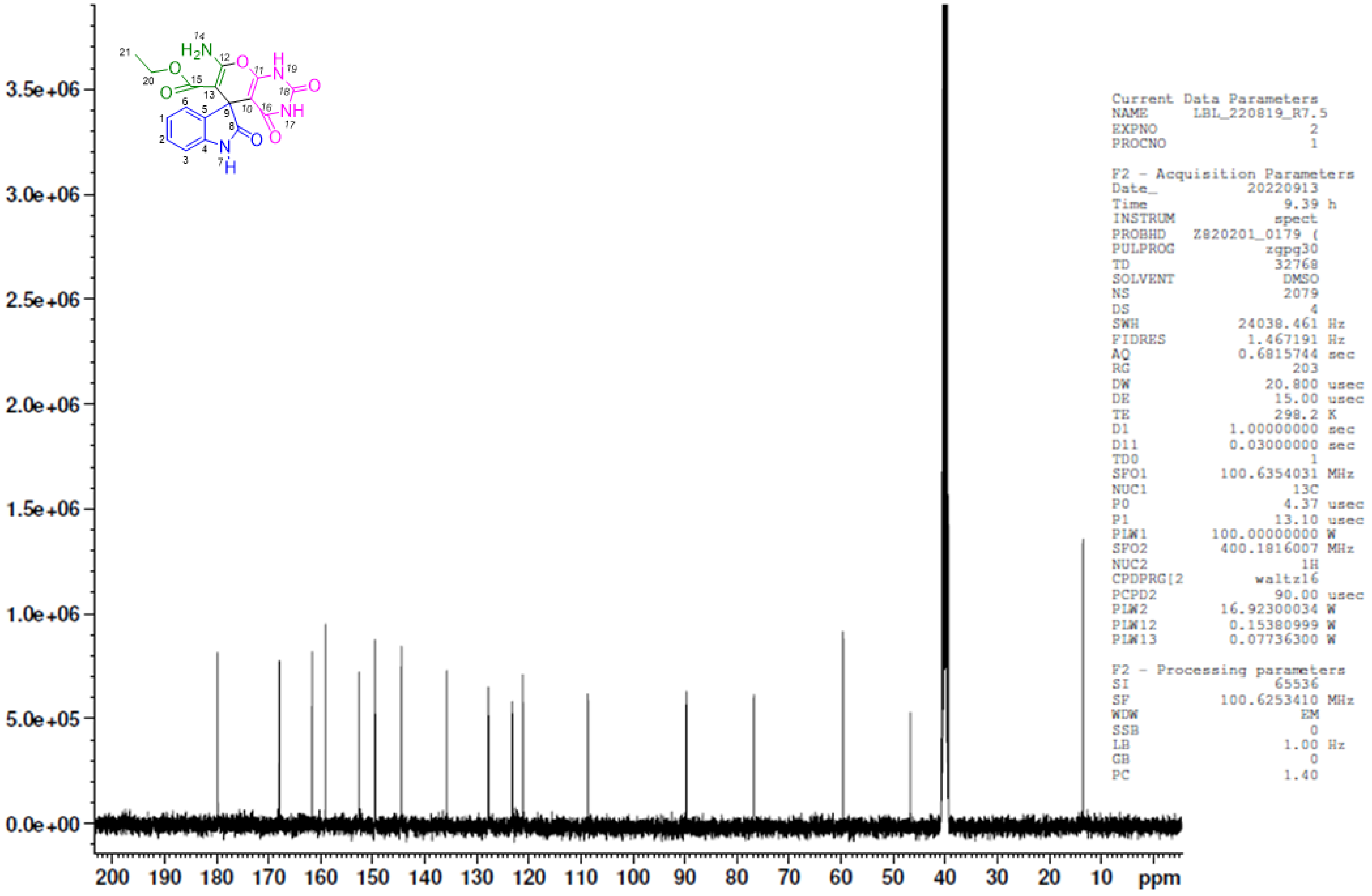

Figure S82. ^{13}C NMR spectrum of compound **1k**.

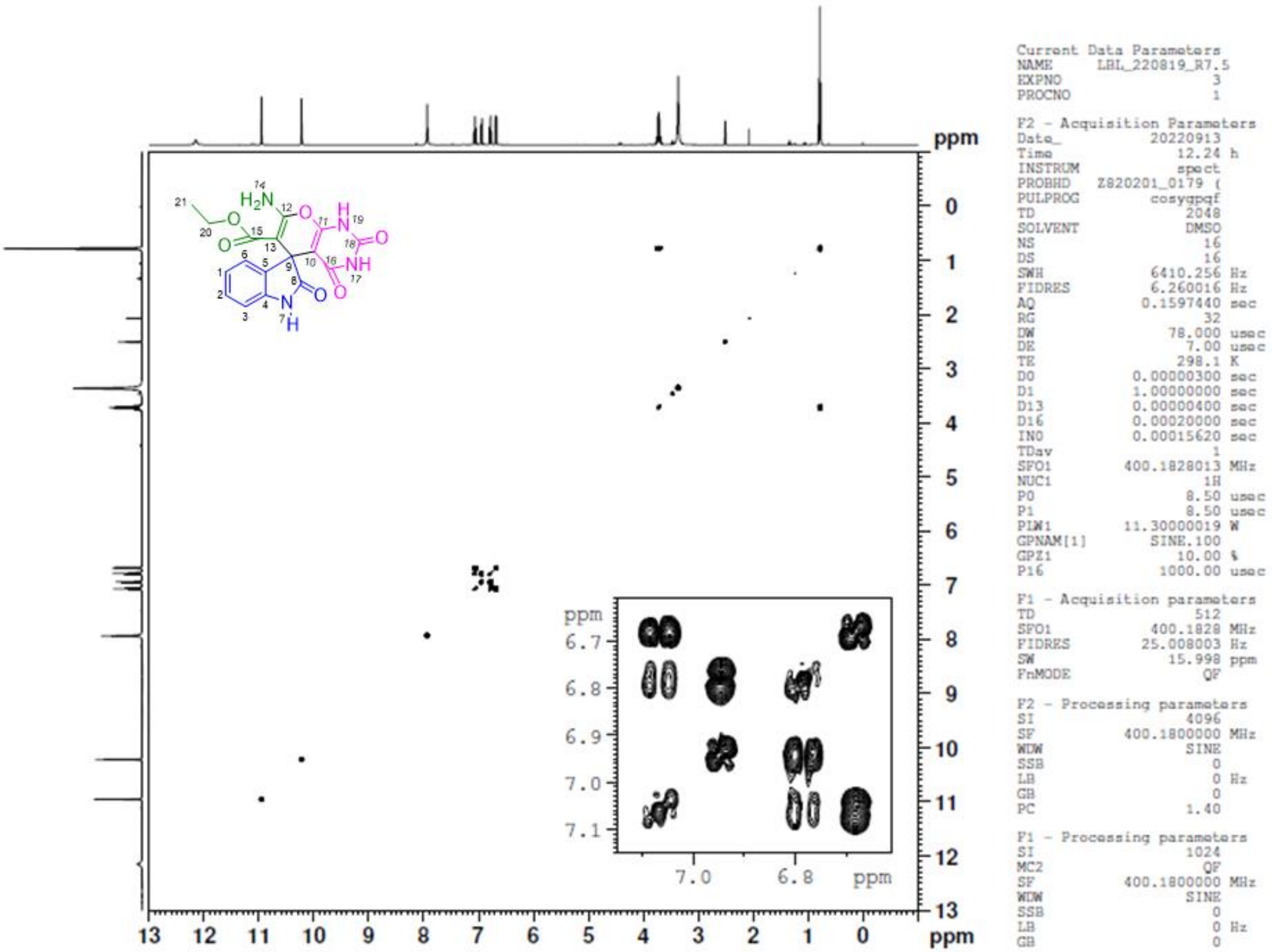

Figure S83. ^1H-^1H COSY NMR spectrum of compound **1k**.

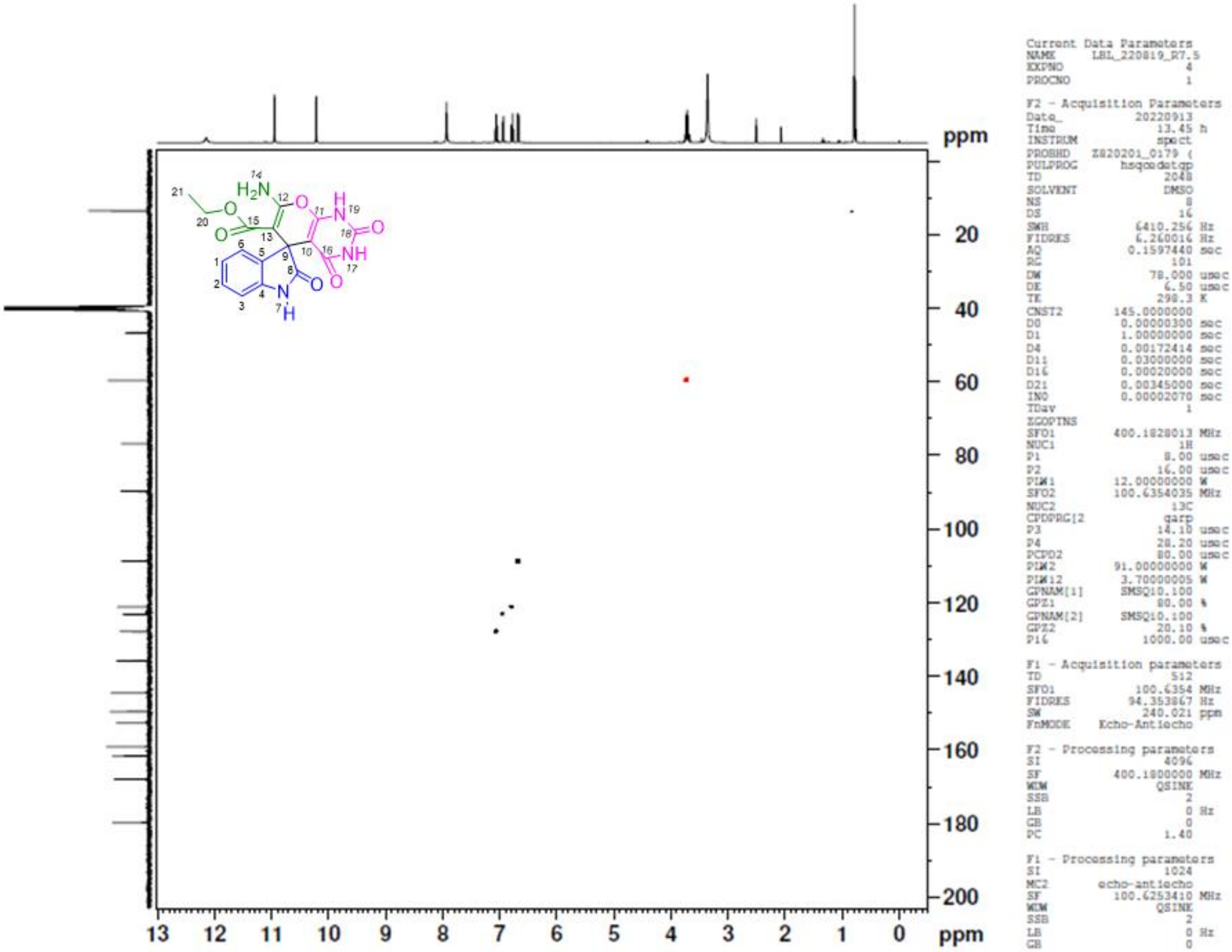

Figure S84. ^{1}H-^{13}C HSQC NMR spectrum of compound **1k**.

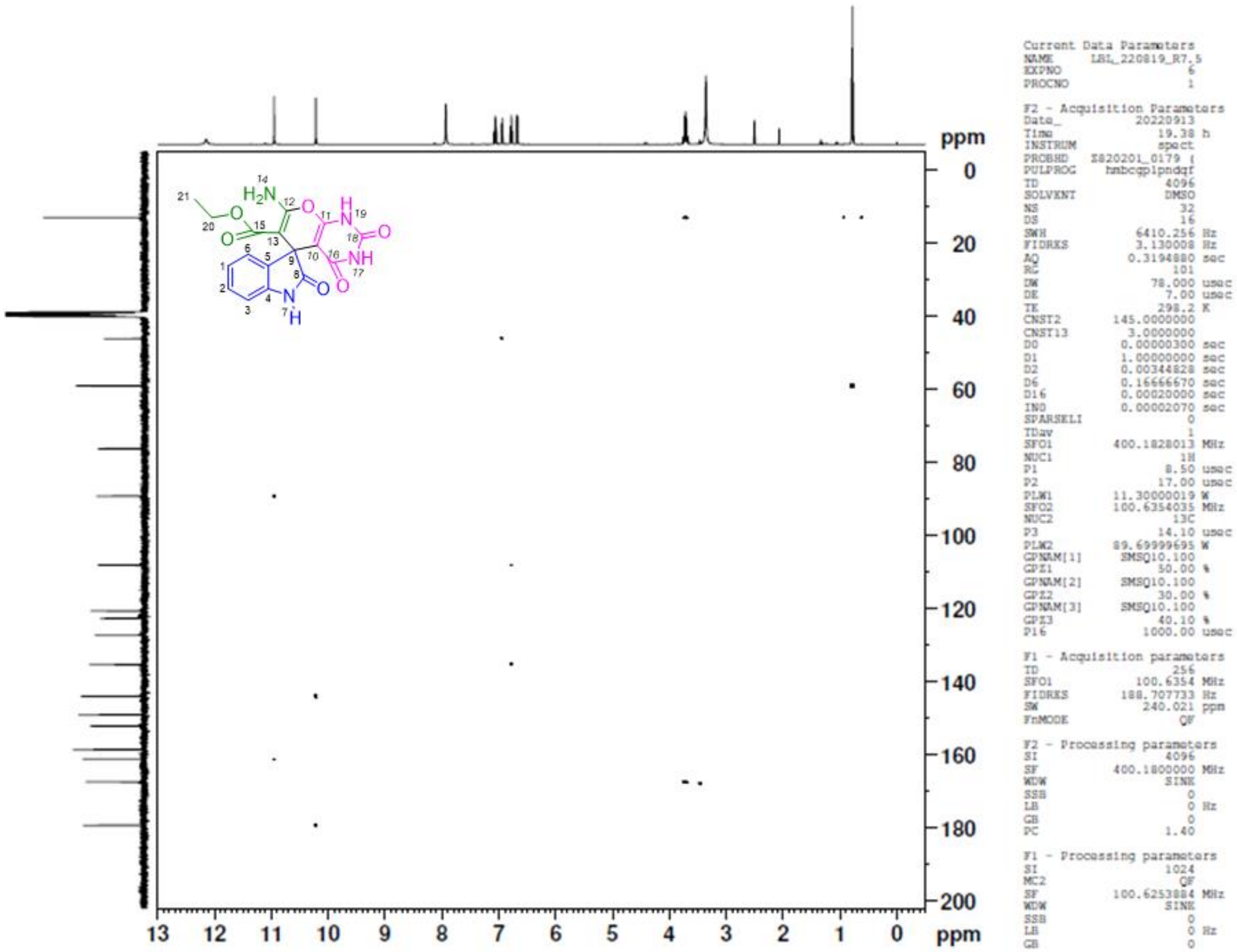

Figure S85. ^{1}H-^{13}C HMBC NMR spectrum of compound **1k** (cnst13 = 3 Hz).

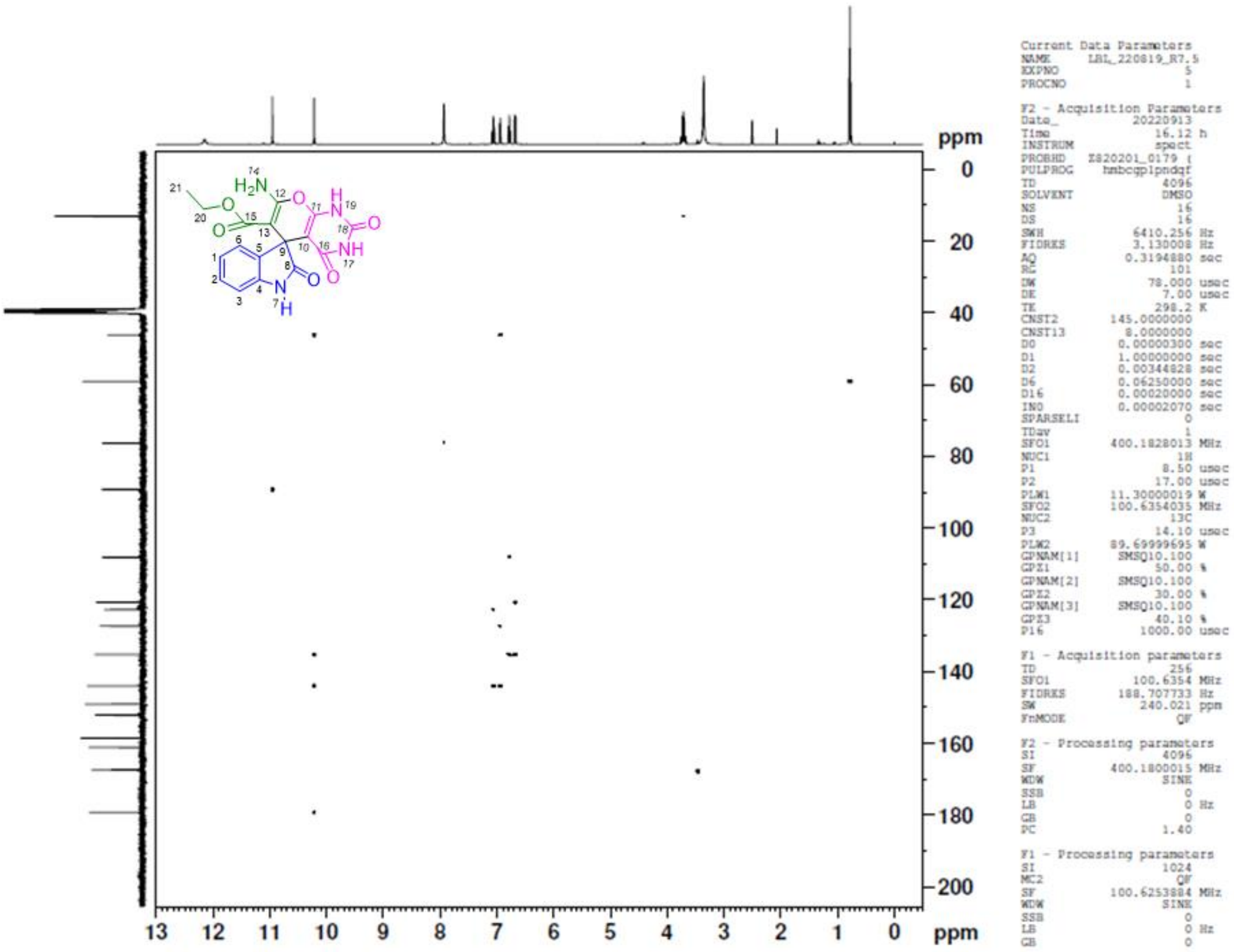

Figure S86. ^{1}H-^{13}C HMBC NMR spectrum of compound **1k** (cnst13 = 8 Hz).

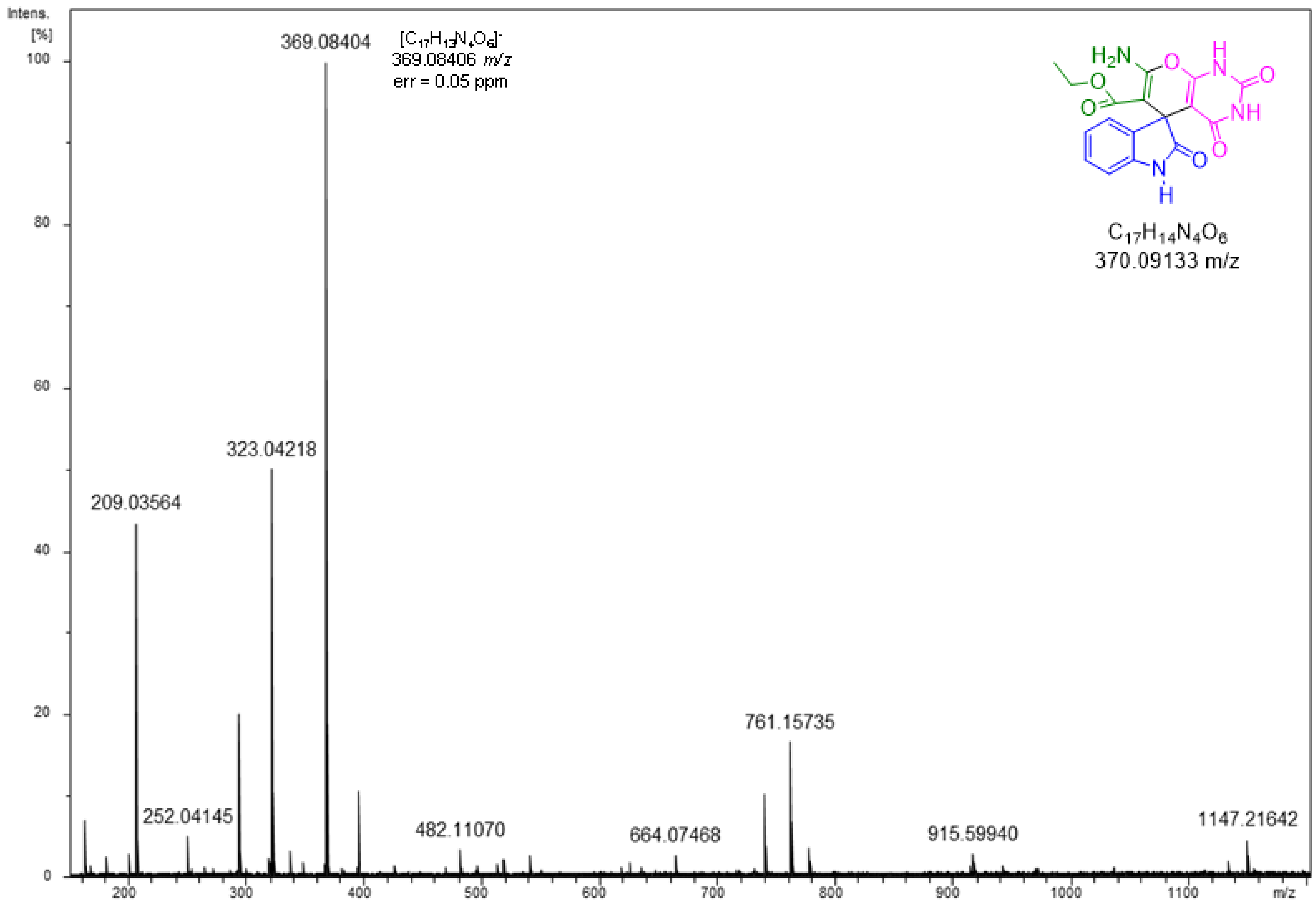

Figure S87. Mass spectrum of compound **1k**.

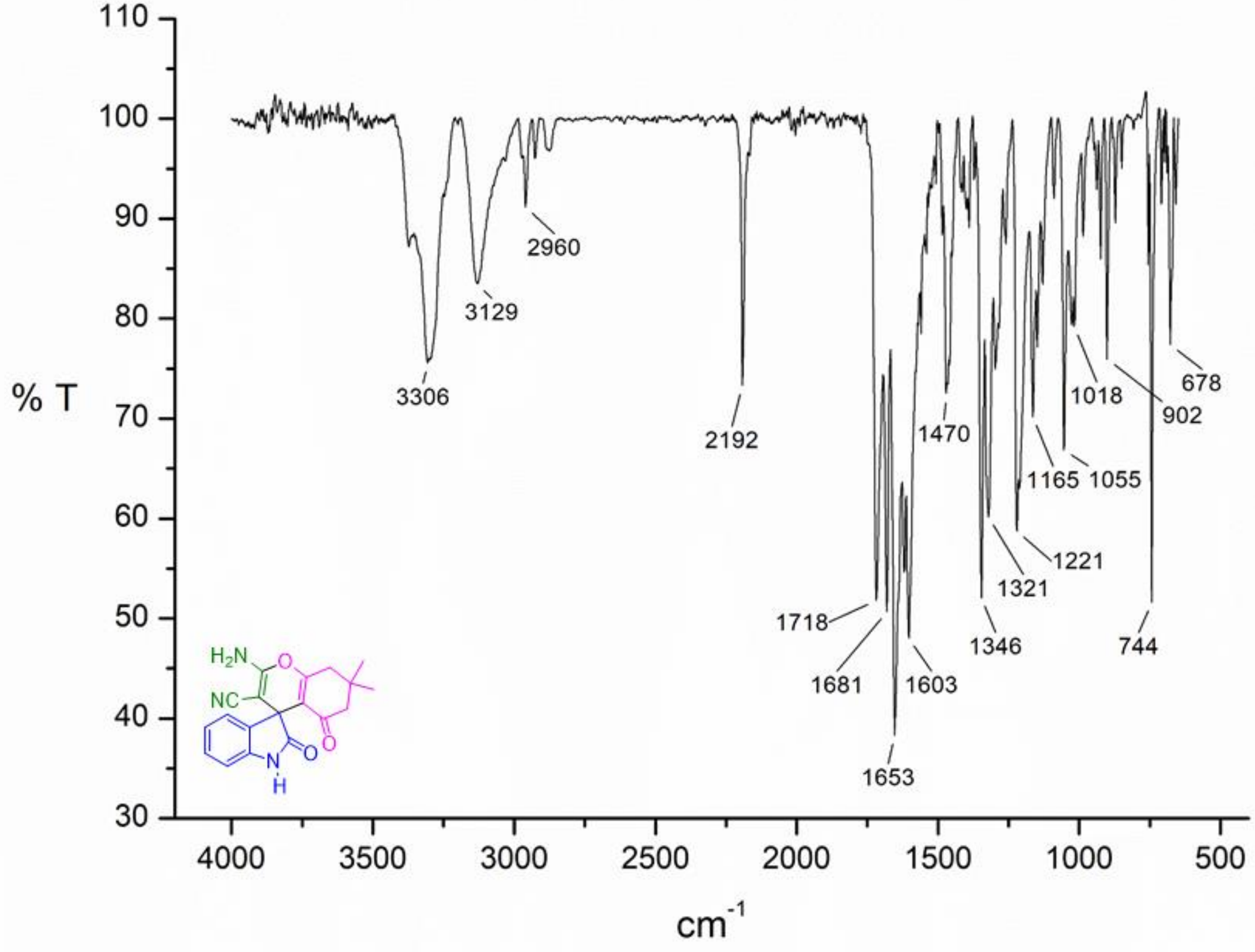

Figure S88. Infrared spectrum of compound **1l**.

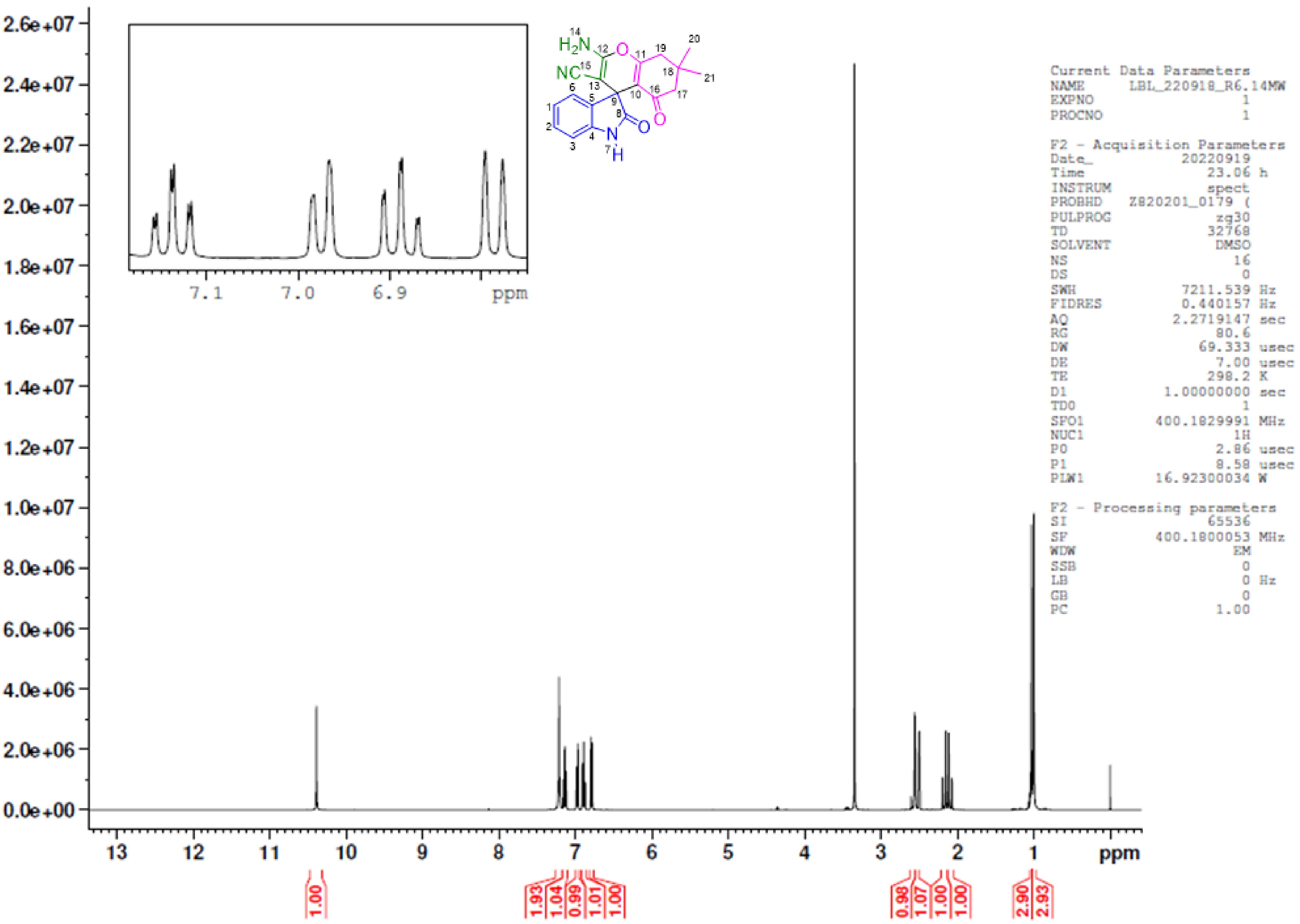

Figure S89. ^{1}H NMR spectrum of compound **1l**.

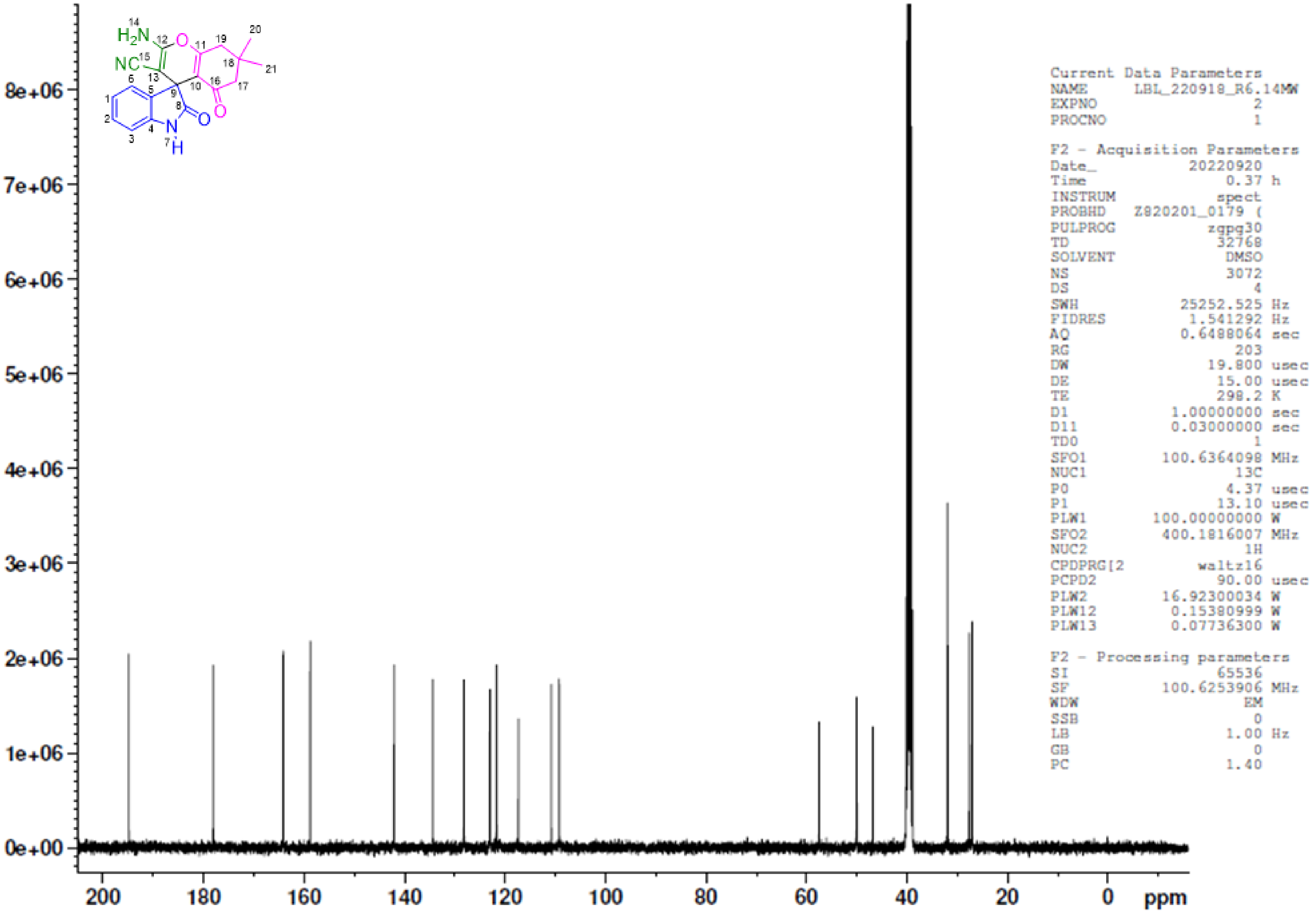

Figure S90. ^{13}C NMR spectrum of compound **1l**.

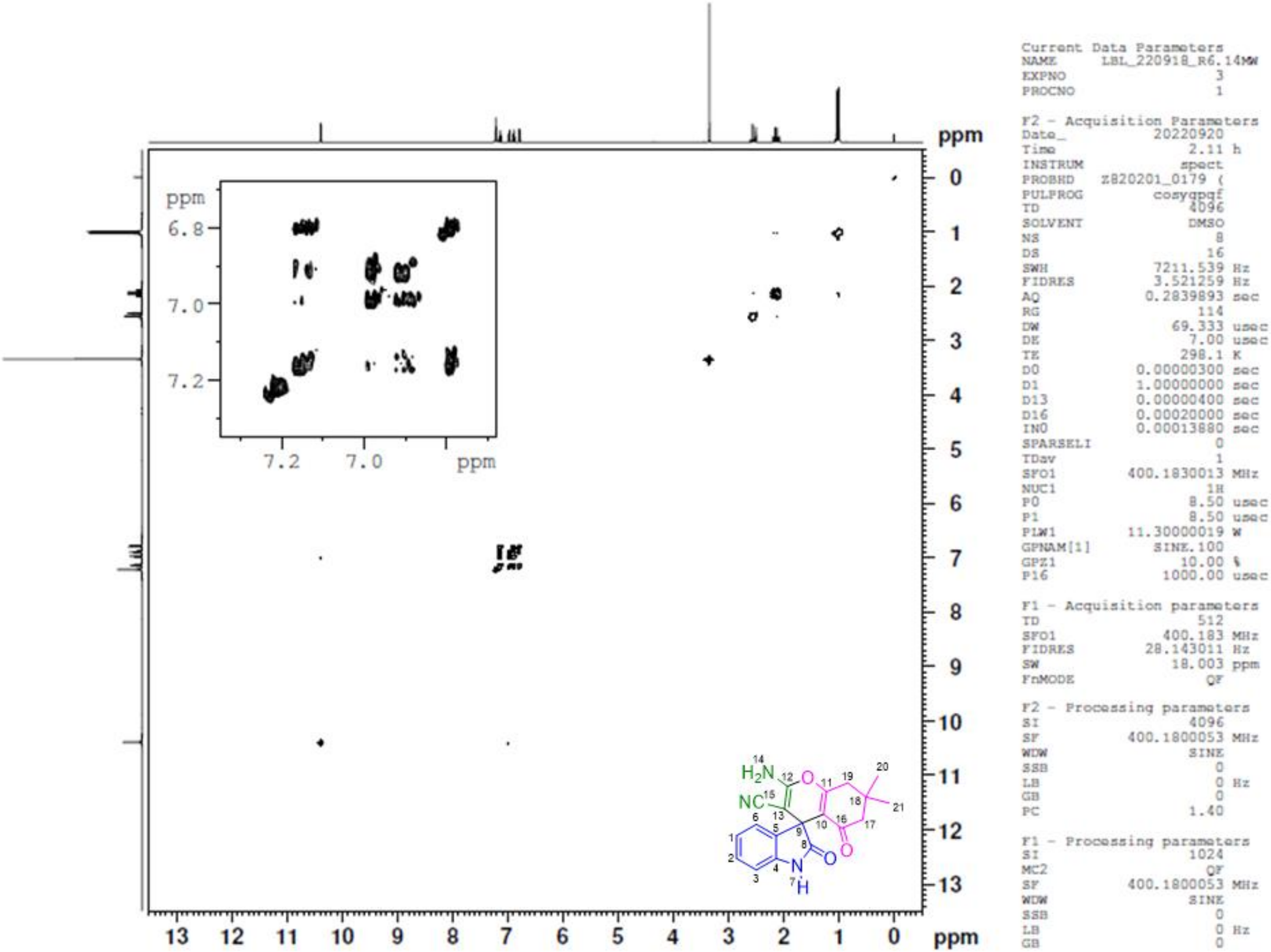

Figure S91. ^{1}H-^{1}H COSY NMR spectrum of compound **1l**.

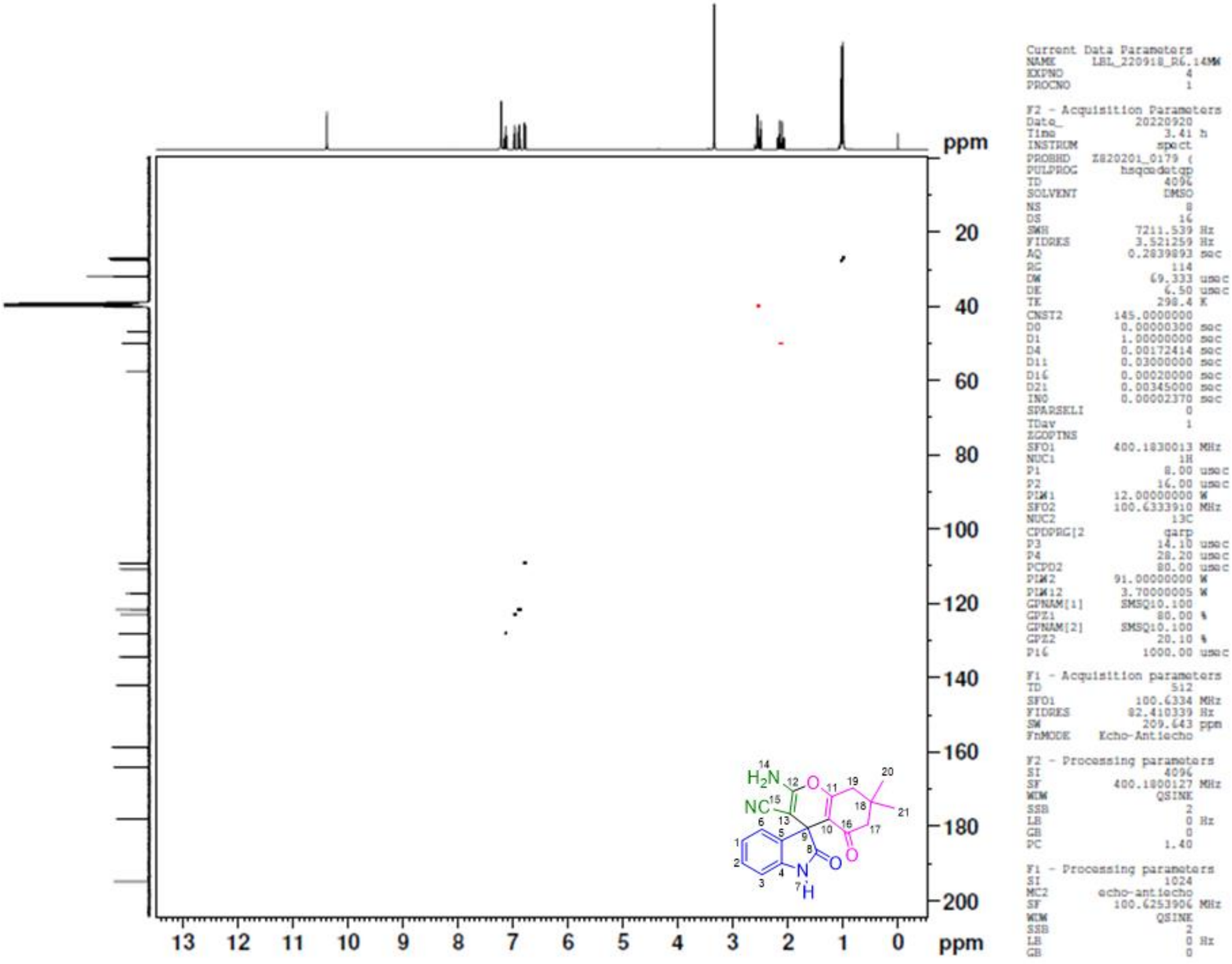

Figure S92. ^{1}H-^{13}C HSQC NMR spectrum of compound **1l**.

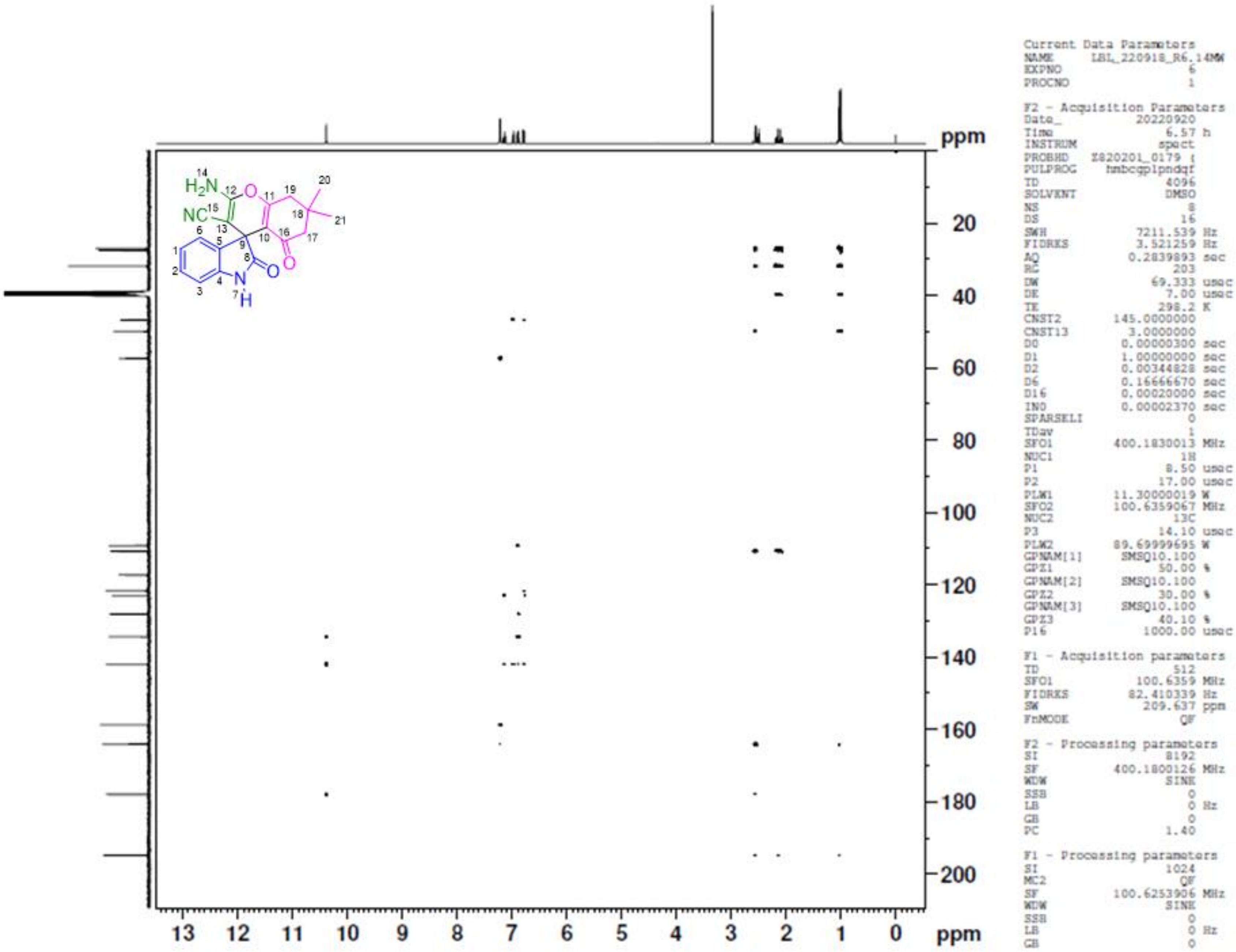

Figure S93. ^{1}H-^{13}C HMBC NMR spectrum of compound **1l** (cnst13 = 3 Hz).

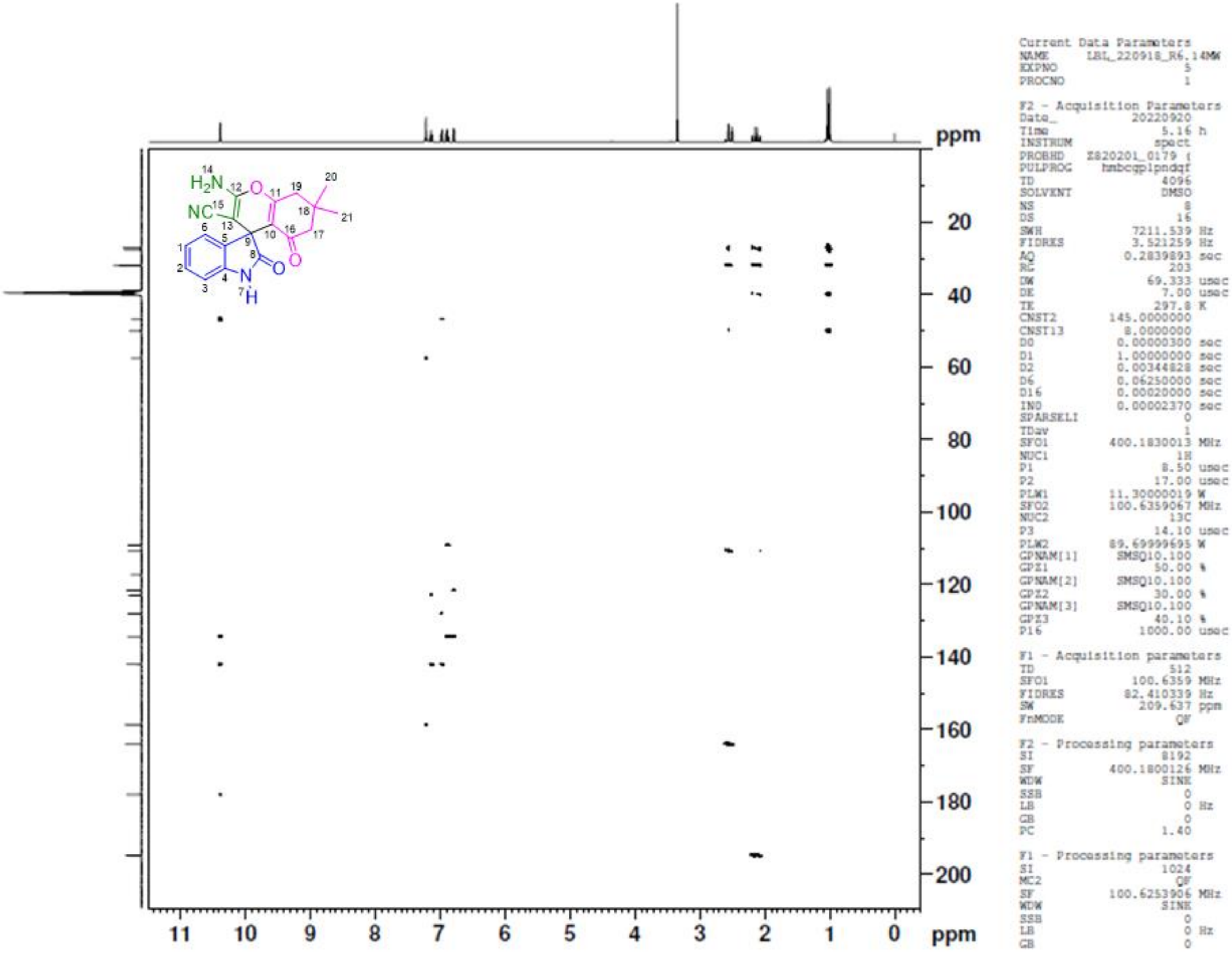

Figure S94. ^{1}H-^{13}C HMBC NMR spectrum of compound **1l** (cnst13 = 8 Hz).

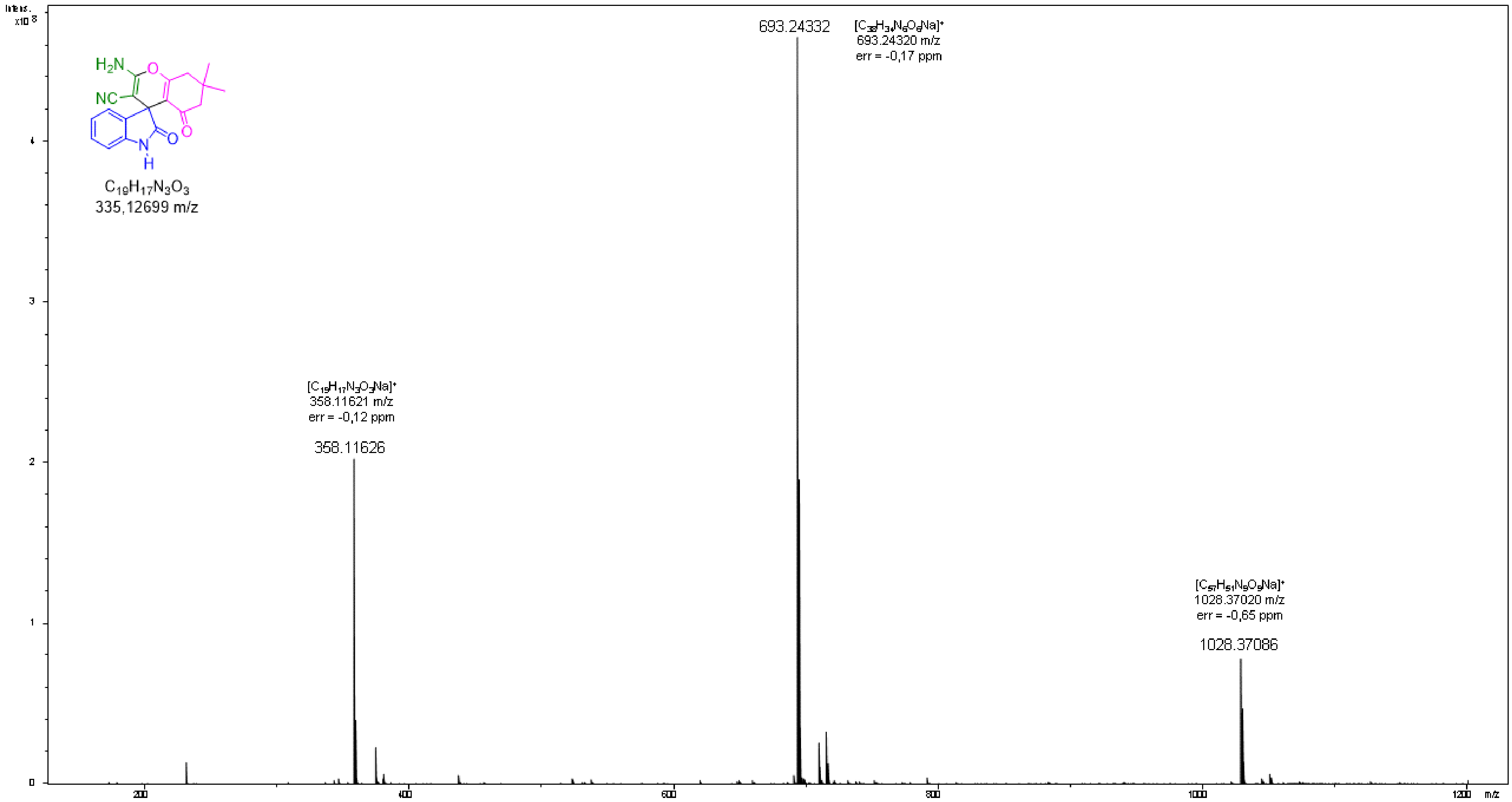

Figure S95. Mass spectrum of compound **1l**.

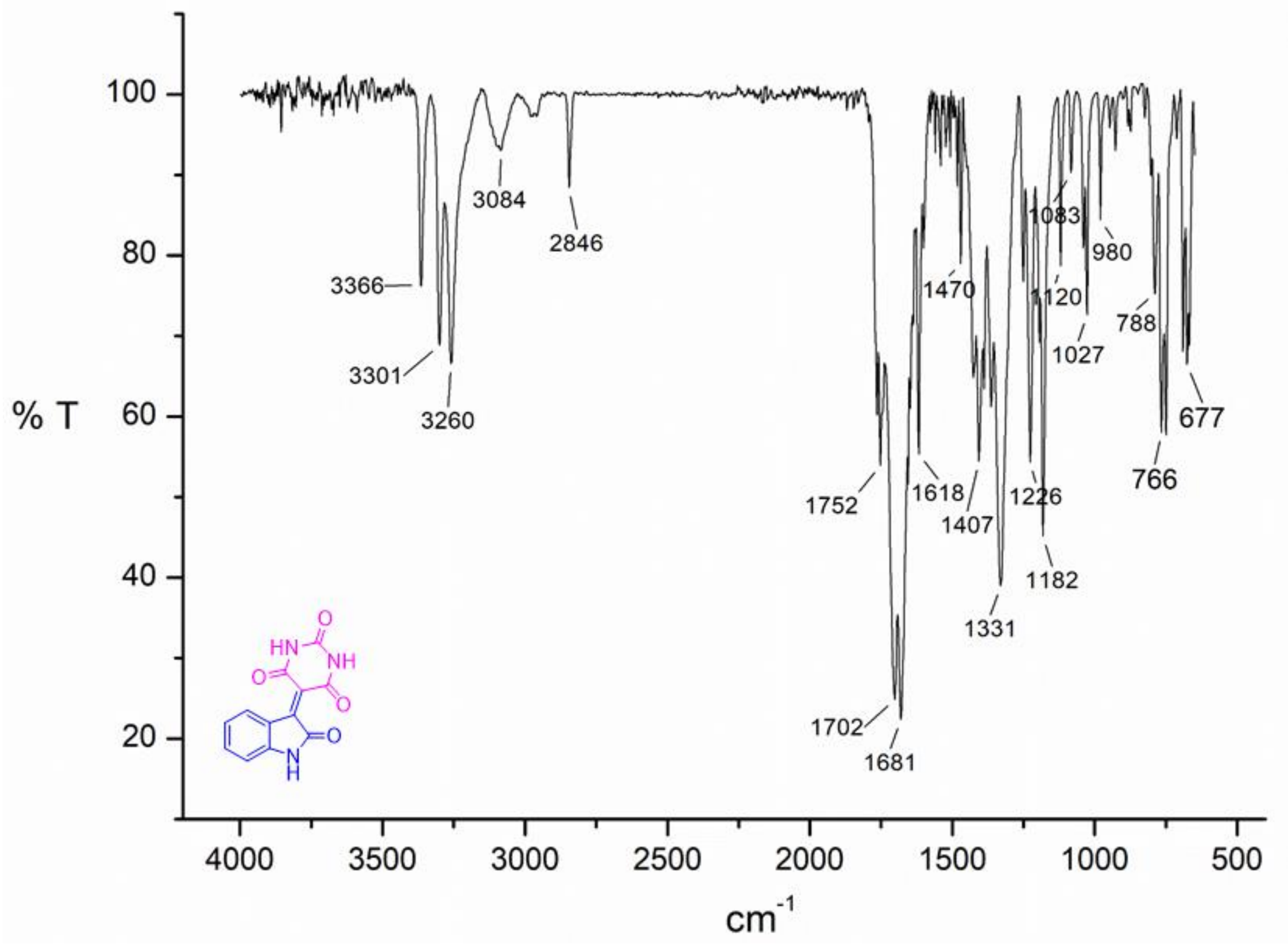

Figure S96. Infrared spectrum of compound **6**.

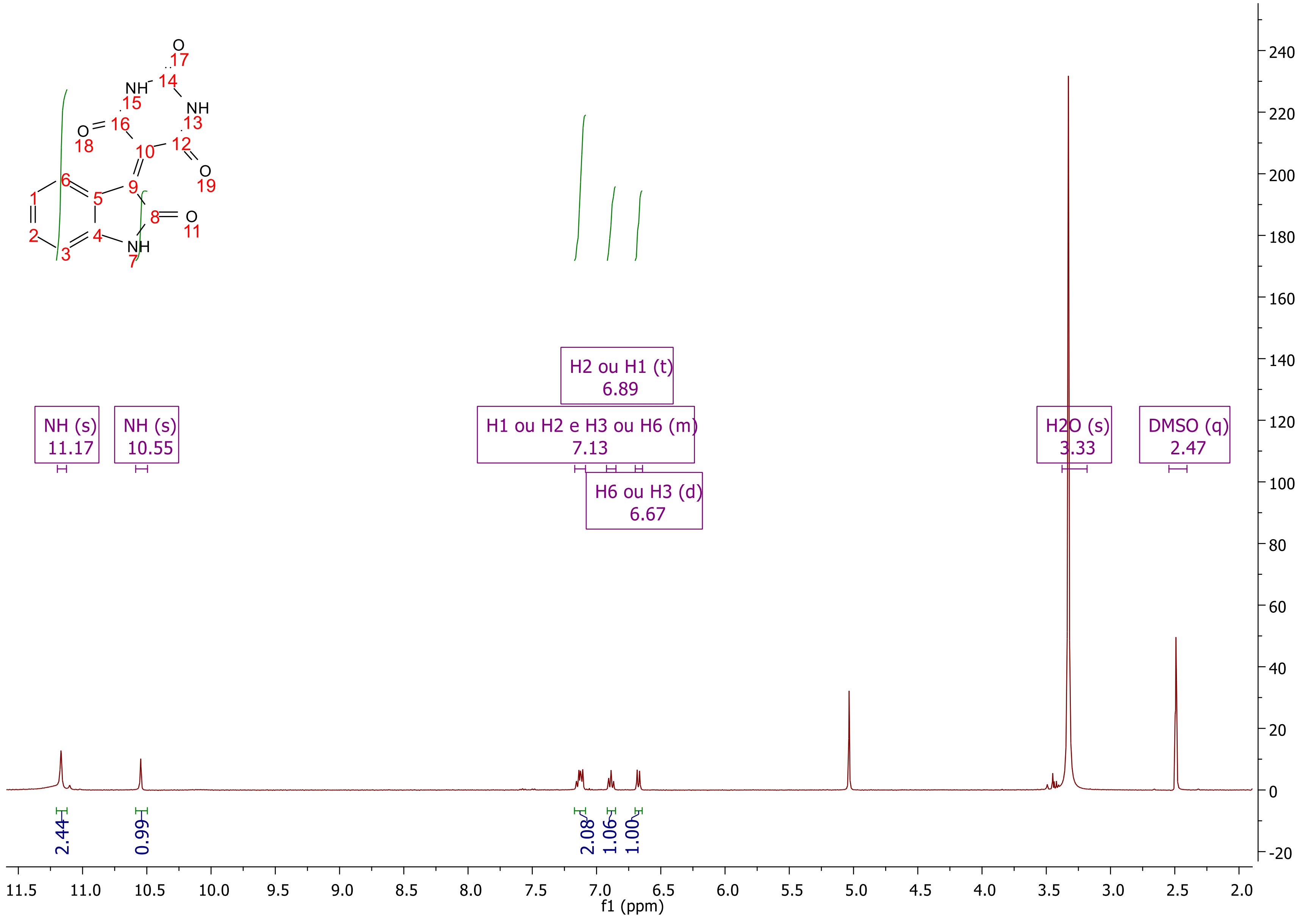

Figure S97. ^{1}H NMR spectrum of compound **6**.

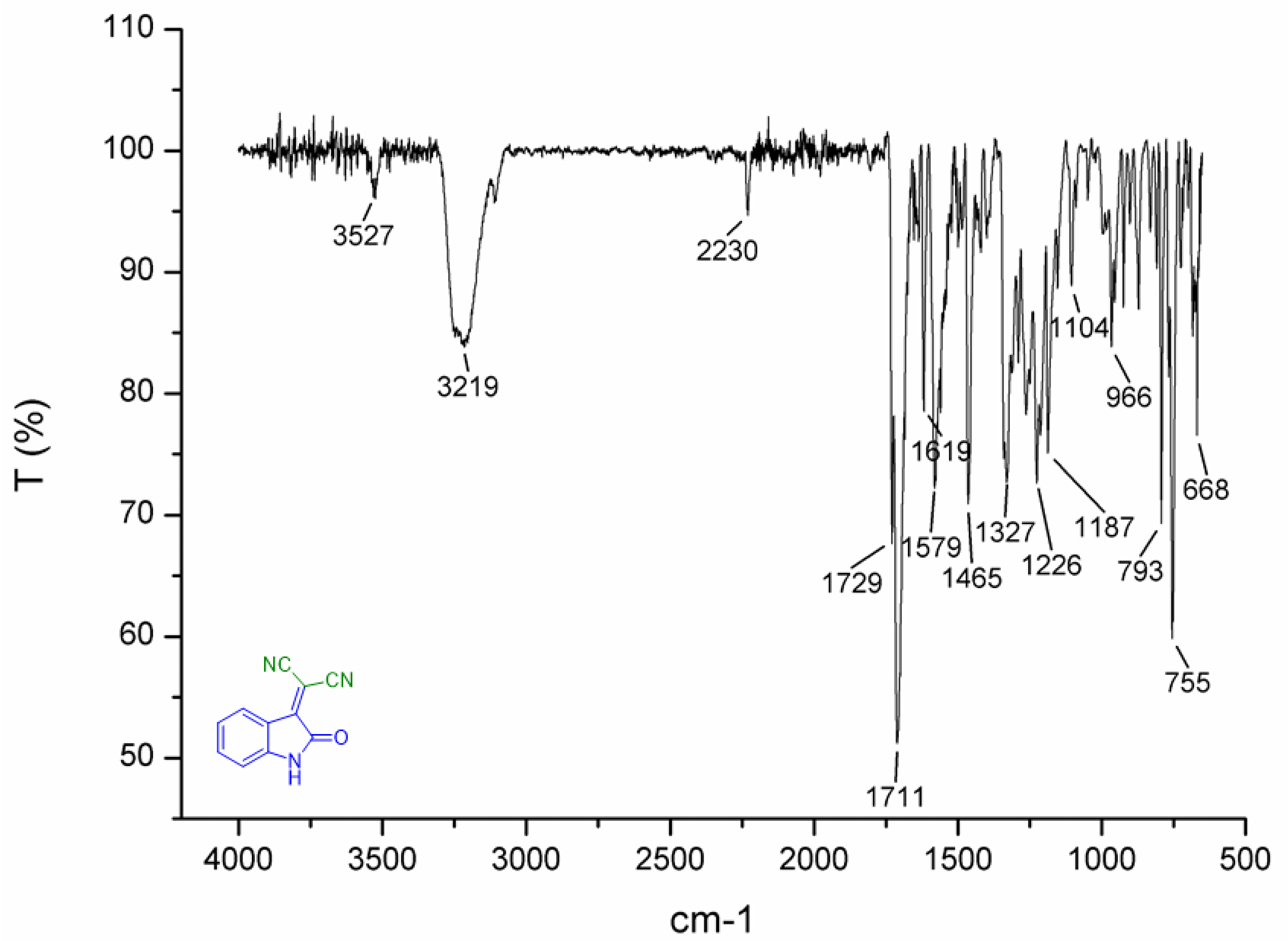

Figure S98. Infrared spectrum of compound **10**.

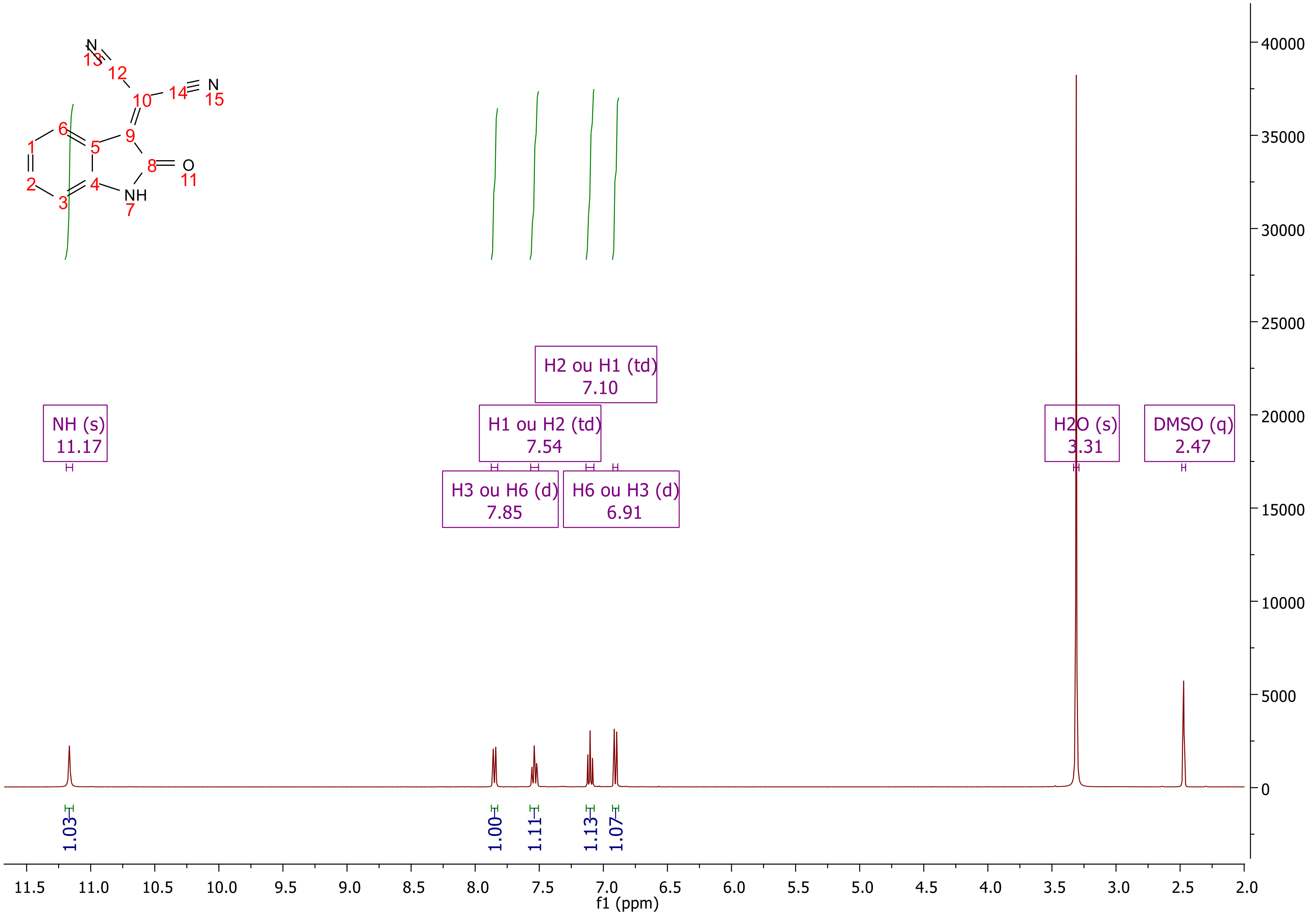

Figure S99. ^{1}H NMR spectrum of compound **10**.
